# An updated review on bluetongue virus: epidemiology, pathobiology, and advances in diagnosis and control with special reference to India

**DOI:** 10.1080/01652176.2020.1831708

**Published:** 2020-11-10

**Authors:** Mani Saminathan, Karam Pal Singh, Jaynudin Hajibhai Khorajiya, Murali Dinesh, Sobharani Vineetha, Madhulina Maity, AT Faslu Rahman, Jyoti Misri, Yashpal Singh Malik, Vivek Kumar Gupta, Raj Kumar Singh, Kuldeep Dhama

**Affiliations:** a Division of Pathology, ICAR-Indian Veterinary Research Institute, Izatnagar, Bareilly, Uttar Pradesh, India; b Animal Science Division, Indian Council of Agricultural Research, New Delhi, India; c Division of Biological Standardization, ICAR-Indian Veterinary Research Institute, Izatnagar, Bareilly, Uttar Pradesh, India; d Centre for Animal Disease Research and Diagnosis, ICAR-Indian Veterinary Research Institute, Izatnagar, Bareilly, Uttar Pradesh, India; e Director, ICAR-Indian Veterinary Research Institute, Izatnagar, Bareilly, Uttar Pradesh, India

**Keywords:** Cattle, sheep, goat, bluetongue virus, epidemiology, Indian scenario, pathogenesis, pathology, immune responses, mice model, diagnosis, vaccination, control

## Abstract

Bluetongue (BT) is an economically important, non-contagious viral disease of domestic and wild ruminants. BT is caused by BT virus (BTV) and it belongs to the genus *Orbivirus* and family Reoviridae. BTV is transmitted by *Culicoides* midges and causes clinical disease in sheep, white-tailed deer, pronghorn antelope, bighorn sheep, and subclinical manifestation in cattle, goats and camelids. BT is a World Organization for Animal Health (OIE) listed multispecies disease and causes great socio-economic losses. To date, 28 serotypes of BTV have been reported worldwide and 23 serotypes have been reported from India. Transplacental transmission (TPT) and fetal abnormalities in ruminants had been reported with cell culture adopted live-attenuated vaccine strains of BTV. However, emergence of BTV-8 in Europe during 2006, confirmed TPT of wild-type/field strains of BTV. Diagnosis of BT is more important for control of disease and to ensure BTV-free trade of animals and their products. Reverse transcription polymerase chain reaction, agar gel immunodiffusion assay and competitive enzyme-linked immunosorbent assay are found to be sensitive and OIE recommended tests for diagnosis of BTV for international trade. Control measures include mass vaccination (most effective method), serological and entomological surveillance, forming restriction zones and sentinel programs. Major hindrances with control of BT in India are the presence of multiple BTV serotypes, high density of ruminant and vector populations. A pentavalent inactivated, adjuvanted vaccine is administered currently in India to control BT. Recombinant vaccines with DIVA strategies are urgently needed to combat this disease. This review is the first to summarise the seroprevalence of BTV in India for 40 years, economic impact and pathobiology.

## 1. Introduction

Bluetongue (BT) is an infectious, non-contagious and arthropod transmitted viral disease of domestic and wild ruminants, caused by BT virus (BTV) that belongs to the genus *Orbivirus* and family Reoviridae (Mertens et al. 1989; Patel and Roy 2014; Ranjan et al. 2015). BTV is a non-enveloped virus with 10 distinct segmented double stranded RNA (dsRNA) genome surrounded by a triple layered icosahedral capsid (Grimes et al. 1998; Ratinier et al. 2011; Patel and Roy 2014). The BTV genome encodes 7 structural (VP1-VP7) and 5 non-structural (NS1-NS5) proteins (Mertens and Diprose 2004; Ratinier et al. 2011; Stewart et al. 2015). Due to its economic impact, BT is a World Organisation for Animal Health (OIE) listed multispecies disease (MacLachlan and Osburn 2006; Gunn et al. 2008; OIE 2008; Rushton and Lyons 2015). BTV infection causes severe direct economic losses due to high morbidity, mortality, stillbirths, abortions, foetal abnormalities, less birth weight in young ones, reduced milk yield and fertility rate, weight loss, early culling as well as meat and fleece losses. Indirect losses are due to trade restrictions imposed on ruminant animal movement, their germplasm and animal products, and expenditure for vaccination, diagnosis, vector control and treatment of clinically pretentious animals (MacLachlan and Osburn 2006; Gunn et al. 2008; Rushton and Lyons 2015; Pinior, Brugger, et al. 2015; Pinior, Lebl, et al. 2015; Grewar 2016; Gethmann et al. 2020). It was estimated that BTV outbreaks caused economic losses of approximately US dollars (US$) 3 billion in 1996 worldwide (Tabachnick 2004). The total cost for prevention of incursion of BTV-8 into Scotland was estimated to be approximately Euro (€) 141 million over the 5-year period between 2009 and 2013 (Gunn et al. 2008). In the US livestock industries, BTV caused losses of US $144 million annually due to trade restrictions and diagnosis for assessing BTV status (Hoar et al. 2003).

Until recently, 28 BTV serotypes have been described, based on the differences in the genome segment-2 (Seg-2) sequence and its translated protein VP2 (Chaignat et al. 2009; Maan et al. 2010, 2011; Maan, Maan, Guimera, Nomikou, Morecroft, et al. 2012; Maan, Maan, Guimera, Nomikou, Singh, et al. 2012; Maan, Maan, Guimera, Pullinger, et al. 2012; Maan, Maan, Nomikou, Guimera, et al. 2012; Maan, Maan, Nomikou, Prasad, et al. 2012; Maan, Maan, Pullinger, et al. 2012; Sperlova and Zendulkova 2011; Coetzee et al. 2012; Schulz et al. 2016; Sun et al. 2016; Bumbarov et al. 2020). The core VP7 protein of BTV is a major group-specific antigen determinant. The BTV-27 was isolated from goats in the island of Corsica, France in 2014 (Schulz et al. 2016), and BTV-28 was isolated from the contaminated live-attenuated sheeppox and lumpy skin disease vaccines in Israel (Bumbarov et al. 2020).

The BTV spread naturally to susceptible hosts by the bite of blood sucking midges of genus *Culicoides* and family Ceratopogonidae (Ander et al. 2012; Maheshwari 2012; MacLachlan and Mayo 2013; Benelli et al. 2017). Recent studies on vectors indicated that *Culicoides oxystoma* and *C. imicola* were found to be mostly responsible for transmission of BTV (Maheshwari 2012; Archana et al. 2016). Other alternative routes of spread are venereal transmission through semen (Bowen and Howard 1984; Kirschvink et al. 2009), contact and oral transmission (Menzies et al. 2008; Backx et al. 2009; Calvo-Pinilla et al. 2010), *in utero* infection by transplacental transmission (De Clercq et al. 2008; Desmecht et al. 2008; Menzies et al. 2008; Darpel et al. 2009; Coetzee et al. 2013; Rasmussen et al. 2013; MacLachlan and Osburn 2017; Saminathan et al. 2020), and mechanical vectors (Bouwknegt et al. 2010; Sperlova and Zendulkova 2011). BT outbreaks are highly seasonal, occur during the late summer and autumn. The BTV outbreaks occur throughout tropical, subtropical and temperate regions of the world, wherever competent vector population exists for dissemination of the virus (Wilson and Mellor 2009; Maheshwari 2012; MacLachlan and Mayo 2013; Ranjan et al. 2015).

BTV usually affects domestic (sheep, goats and cattle) and wild (deer, pronghorn antelope and bighorn sheep) ruminants, camelids, elephants (Robinson et al. 1967; MacLachlan 1994, 2004; Patton et al. 1994; Johnson et al. 2006; MacLachlan et al. 2008, 2009; Batten et al. 2013; Coetzee et al. 2014; Niedbalski 2015; Ranjan et al. 2015), domestic and wild carnivores (Alexander et al. 1994; Brown et al. 1996; Jauniaux et al. 2008; Falconi et al. 2011; Dubovi et al. 2013). Among ruminants, severe clinical disease is mostly seen in sheep, white-tailed deer, pronghorn antelope, desert bighorn sheep, mouflon, llamas and alpacas; whereas, cattle, goats and camelids usually show asymptomatic or sub-clinical disease (Backx et al. 2007; MacLachlan et al. 2009; Schulz et al. 2012). However, outbreaks of BTV-8 in Europe during 2006 caused clinical disease in both goats and cattle (De Clercq et al. 2008; Wilson and Mellor 2009; Coetzee et al. 2013). Clinical signs of BT are fever, serous to bloody nasal discharge, later on mucopurulent, hyperaemia and oedema of lips, face, ears and sub-maxillary region (‘monkey-face’ appearance), oral erosions and ulcers, cyanosis of tongue, lameness with coronitis, repiratory distress and muscular necrosis to culminate in debility and death (MacLachlan 1994, 2004; Backx et al. 2007; MacLachlan et al. 2008, 2009; Worwa et al. 2009; Umeshappa, Singh, Channappanavar, et al. 2011). BTV induced lesions are due to direct injure to the endothelial cells of microvasculature resulting in increased vascular permeability, haemorrhages, fluid exudations, thrombosis, and tissue infarction (Pini 1976; Mahrt and Osburn 1986; MacLachlan 1994, 2004; Darpel et al. 2007, 2009; MacLachlan et al. 2009; Drew et al. 2010).

Presently, BT is endemic in India. Among 28 serotypes of BTV, 23 serotypes (except 22, 25–28) have been reported from India based on the presence of neutralising antibodies and virus isolation (VI). So far, fifteen serotypes (BTV-1–6, 9, 10, 12, 16–18, 21, 23, and 24) were identified by VI, while 22 serotypes (BTV-1–20, 23, and 24) were reported by serological testing (Prasad et al. 1992; Sreenivasulu et al. 2004; Joardar et al. 2009; Chauhan et al. 2014; Ranjan et al. 2015; Krishnajyothi et al. 2016; Rao et al. 2016; Hemadri et al. 2017). In several states of India, BTV antibodies were detected in goat, cattle, camel, buffalo and mithun; however, clinical disease was not frequently reported in these species (Joardar et al. 2015; Maan et al. 2017; Shah et al. 2017; Karam et al. 2018).

BTV is a potent inducer of type 1 interferons (IFN-1s) including IFN-α and IFN-β (Jameson et al. 1978; Fulton and Pearson 1982; MacLachlan and Thompson 1985; Vitour et  al. 2014; Saminathan et al. 2018, 2019). The IFN-Is plays a critical role in the anti-viral innate immune responses. The plasmacytoid dendritic cells (pDCs), produce significant amount of IFN-Is. A temporal relationship between BTV replication, viremia and induction of IFN-Is were reported and interferon (IFN) peak concentration decreased a BTV titre (Foster et al. 1991; Saminathan et al. 2018). Genetically IFN-α/β gene knockout mice (Calvo-Pinilla, Rodriguez-Calvo, Anguita, et al. 2009; Calvo-Pinilla et al. 2010; Ortego et al. 2014; Marín-López et al. 2014) and blocking of IFN-α/β signaling in wild-type adult mice using monoclonal (clone: MAR1-5A3) antibodies (Sheehan et al. 2006, 2015; Smith et al. 2017; Saminathan et al. 2020) leads to susceptibility of mice to BTV infection.

Diagnosis of BT is more essential for control and eradication of disease, and to ensure safe trade of animals and their products between the countries/regions (Gould et al. 1989; Afshar 1994; Billinis et al. 2001; Dadhich 2004; Hamblin 2004; OIE 2008). The presumptive diagnosis of the disease can be made based on the clinical signs and lesions (Backx et al. 2007; Darpel et al. 2007; MacLachlan et al. 2008, 2009; Batten et al. 2013; Coetzee et al. 2014). The BTV antigen identification methods include VI either in embryonated chicken eggs (ECEs) or in cell lines, reverse-transcription polymerase chain reaction (RT-PCR), real-time RT-PCR, immunofluorescence test, sandwich enzyme-linked immunosorbent assay (s-ELISA), dot immunoperoxidase assay (DIA), virus neutralisation test, and immunohistochemistry (Afshar 1994; Billinis et al. 2001; Dadhich 2004; Hamblin 2004; OIE 2008; Ranjan et al. 2015; Rojas et al. 2019). BTV antibodies are detected by using complement fixation test (CFT), agar gel immunodiffusion (AGID), competitive ELISA (c-ELISA), indirect ELISA (i-ELISA), and serum neutralisation test (SNT) (Afshar 1994; Dadhich 2004; Hamblin 2004; OIE 2008; Ranjan et al. 2015; Rojas et al. 2019). Polyacrylamide gel electrophoresis (PAGE) was used for the identification of genome segments of BTV (Ranjan et al. 2015; Rojas et al. 2019). The RT-PCR, AGID and c-ELISA are found to be sensitive and OIE recommended test for diagnosis of BTV for international trade (Jochim 1985; OIE 2008; Maan, Maan, Belaganahalli, et al. 2012; Ranjan et al. 2015; Rojas et al. 2019). Mass vaccination, vector control, intensive serological and entomological surveillance, forming restriction zones and sentinel program are the most effective method for the control and eradication of BT (Kutzler and Weiner 2008; Roy et al. 2009; Caporale and Giovannini 2010; Zientara et al. 2010; Pandrangi 2013; Calvo-Pinilla et al. 2014; McVey and MacLachlan 2015; Feenstra and van Rijn 2017; Mayo et al. 2017; van Rijn 2019). The pentavalent inactivated adjuvanted vaccine containing BTV-1, -2, -10, -16 and -23 has been used for the control of BT in India (Reddy et al. 2010; Rao et al. 2016).

The present updated review summarizes the various aspects of BTV like history, epidemiology, Indian scenario, economic impact, species affected and reservoirs, transmission, pathobiology, immune responses, mice models for BTV infection, and advances in diagnosis, vaccination and control of this economically important viral disease.

## 2. History of BTV

The first official report of BTV infection was from the Cape Province of South Africa in the late 18^th^ century, following import of fine-wool Merino sheep from Europe (Spreull 1905). Initially, BT was called as ‘epizootic catarrh’ or ‘fever’ or ‘malarial catarrhal fever of sheep’ or ‘epizootic malignant catarrhal fever of sheep’, due to the erroneous belief that BT was caused by an intraerythrocytic parasite (Hutcheon 1902; Spreull 1905). The English translated term ‘Bluetongue’ was first introduced by Spreull (1905) and derived from the Afrikaans word ‘bloutong’ or ‘Blaauwtong’, which was used by Afrikaans farmers after observing the cyanosis of tongue in clinically affected sheep (Spreull 1905; MacLachlan et al. 2009). After observing oral lesions, Afrikaans farmers also called the BT as ‘Bekziekte’, which means ‘mouth sickness’. The disease was first reported in cattle in 1933 (Bekker et al. 1934), and the clinical signs were similar to that of foot-and-mouth disease. Hence, the disease was called as ‘pseudofoot-and-mouth disease’ or ‘sore-mouth’ or ‘seerbeck’. BTV is a filtrable virus and it was first time reported by Theiler in 1906 (Sperlova and Zendulkova 2011). BTV serotype-4 was the first BTV to be identified in South Africa in 1906 (Coetzee et al. 2012).

Before 1940s, occurrence of BT was restricted to South Africa (MacLachlan et al. 2009; Coetzee et al. 2012). The first outbreak of BTV, outside the African continent was reported in sheep from Cyprus (Eastern Mediterranean) in 1943, and BTV-3 was isolated from this outbreak (Gambles 1949; MacLachlan 2004); however, there are some indications that BT had been there since 1924. Again, BTV-4 was isolated from Cyprus in 1969. Then, BTV was spread to Israel in 1943–44 (Sperlova and Zendulkova 2011), and it was reported in Texas, USA in 1948 (Hardy and Price 1952). Mckercher et al. (1953) isolated the BTV for the first time from the United States. The initial isolate was identified as BTV serotype-10, followed by BTV-11 in 1955, BTV-17 in 1962, and BTV-13 in 1967 (Barber 1979). During 1956–57, a major epizootic occurred due to BTV-10 in Portugal and Spain (Iberian peninsula), where 1,79,000 sheep died resulting in 75% mortality rate (Lopez and Botija 1958).

Subsequently, BT was spread to Europe and then to North America, Middle East, and Asia (St George et al. 1978; MacLachlan 2004). In Germany, BTV was first time reported in late August 2006. In the Netherlands, BTV was first time reported in sheep on 17 August 2006 and little later time in goats and cattle in same year (Dercksen et al. 2007). BTV was first recorded from Greece in 1998, and subsequently, it spread to Bulgaria, Turkey, Montenegro, Serbia, Macedonia, and Kosovo in 1999 (Taylor and Mellor 1994). BTV was also reported in Sicily, Sardinia, Italy, Corsica, Mallorca, and Menorca in 2000. BTV was first time reported in Croatia in 2001 and subsequently, it spread to Albania and Bosnia in 2002 (Sperlova and Zendulkova 2011). In North America, BTV-1 was first isolated from white-tailed deer in Louisiana in 2004. BTV serotype-2 was first reported from Florida in 1982 and BTV-12 from Texas in 2008 (Johnson et al. 2006; Schirtzinger et al. 2018).

In the Indian sub-continent, BTV was first reported from Pakistan in 1959 (Sarwar 1962). The BTV-16 serotype was the first BTV isolated in Pakistan (Sarwar 1962). Subsequently, first BT outbreak was reported amongst sheep and goats in Maharashtra state of India in 1964 (Sapre 1964). In China, BTV was first isolated from Yunnan Province in 1979 (Sun et al. 2016; Yang et al. 2017). The first BTV outbreak was reported in Indonesia from Suffolk sheep imported from South Australia in 1981. The serological evidence of BTV was first reported from Malaysia in 1977 (Daniels et al. 2004; Pritchard et al. 2004). BTV serotype-26 was first time isolated from Kuwait (Maan et al. 2011). In Australia, BTV was first time reported from the Northern Territory in 1975 (St George et al. 1978). BTV serotype-5 was first time isolated in cattle from Northern Territory in Australia in 2015. In South America, BTV was first time reported from Brazil in 1978 (Sugiyama et al. 1982; Sperlova and Zendulkova 2011; McVey and MacLachlan 2015).

## 3. Structure of BTV

BT is caused by BTV belonging to the genus *Orbivirus*, family Reoviridae and subfamily Sedoreoviridae (Mertens and Diprose 2004; Sperlova and Zendulkova 2011; Stewart et al. 2015). The virus is non-enveloped, icosahedral symmetry, about 80–90 nm in diameter, and having 10 segmented genome of linear dsRNA (Verwoerd et al. 1972; Mertens and Diprose 2004; Stewart et al. 2015). The 10 viral genome segments encode 7 structural (VP1–VP7) and 5 non-structural (NS1, NS2, NS3/NS3A, NS4 and NS5) proteins (Van Dijk and Huismans 1988; Ratinier et al. 2011). Each genome segment encodes a single protein except Seg-9 and -10. The Seg-9 encodes VP6 and NS4 proteins. Likewise, Seg-10 encodes NS3 and NS3A proteins (Ratinier et al. 2011; Stewart et al. 2015). The full genome size of BTV-10 is approximately 19.2 kbp in length (Roy et al. 1990). The size of genome segments ranges from 3954 to 822 bp, in the order of decreasing molecular weight (Seg-1 to Seg-10). The non-coding region of BTV at 5′ end varies from 8 to 34 bp in length and at 3′ end varies from 24 to 116 bp in length (Mertens and Diprose 2004). The dsRNA of BTV contains 57% AU (adenine and uracil) and 43% GC (guanine and cytosine), with conserved hexanucleotides (GUUAAA at 5′ end and ACUUAC at 3′ ends of the positive strand) at the non-coding end of both 5′ and 3′ terminal sequences (Mertens et al. 1989; Mertens and Diprose 2004; Stewart et al. 2015).

The mature BTV virion contains 3 concentric capsid layers. The diffuse outer protein layer (VP2 and VP5) and transcriptionally active internal core is formed by two layers, namely intermediate or middle layer (VP7) and an icosahedral inner most sub-core (VP3 and 3 minor enzymatic proteins VP1, VP4 and VP6) (Mertens et al. 1989; Mertens and Diprose 2004). The outer most protein layer is composed of 60 trimers of VP2 (110 kDa size and encoded by Seg-2) to form triskelion motifs that are interspersed with 120 trimers of VP5 (encoded by Seg-6) (Hewat et al. 1994). The VP2 protein acts as ligand for cell receptors of mammals, which facilitates the clathrin-mediated endocytosis. The VP2 protein is a major determinant of BTV serotype (VP5 plays minor role through stearic interaction with VP2) and responsible for the stimulation of serotype-specific neutralizing antibodies and hemagglutination (Mertens et al. 1989; Hassan and Roy 1999). The VP5 protein is arranged as trimers, which form the outer layer globular motifs of BTV particle (Nason et al. 2004). The VP5 protein makes strong contact with underlying layer of VP7 and VP2 proteins. The VP5 is a viral fusion protein (59 kDa size and 526–527 amino acids) and during receptor-mediated endocytosis it aids in the entry of BTV cores into the cytoplasm of host cells (Hassan et al. 2001; Patel and Roy 2014). In contrast to VP2, VP5 protein is significantly more conserved (Mertens et al. 1989). The VP5 protein plays a minor role in eliciting antibody responses (Hassan et al. 2001). A recent study showed that VP5 has a membrane permeabilization property by interacting with host cell endosomal membrane, which assists the release of mature virion from endosomal compartment to cytoplasm (Hassan et al. 2001). Further, VP5 plays a crucial role in syncytium formation in infected host cells by ensuring pH-dependent conformational changes and by fusing to a transmembrane anchor (Patel and Roy 2014). The VP5 with N-terminal amphipathic helix is highly cytotoxic to the host cells (Hassan et al. 2001). Both VP2 and VP5 are involved in cell-attachment and entry during the early stages of infection (Verwoerd et al. 1972).

The intermediate layer of internal core is composed of 260 trimers of VP7 (38 kDa size, 349 amino acids and encoded by Seg-7), which surrounds the sub-core surface and epitopes are exposed in the outer protein layer of BTV virion (Verwoerd et al. 1972; Huismans et al. 1987; Hewat et al. 1994; Grimes et al. 1998; Mertens and Diprose 2004). The VP7 is highly conserved and represents as an immunodominant BTV-specific antigen. The VP7 protein is a major determinant of serogroup that determines the several distinct phylogenetic groups (Ranjan et al. 2015). It provides additional support and rigidity to the inner sub-core. The VP7 protein facilitates the attachment and exhibits strong infectivity of BTV to *Culicoides* midges/insect cells but is poorly infectious to mammalian cells or host (Mellor 1990; Tan et al. 2001). The VP7 antigen is commonly used in c-ELISA assay to detect anti-BTV antibodies (Mertens and Diprose 2004).

The inner most sub-core is composed of 60 dimers of highly conserved VP3 protein (100 kDa size, 901 amino acids inner and encoded by Seg-3), which are arranged in icosahedral symmetry (Loudon and Roy 1992). It binds with RNA molecules and tri-protein transcriptase complex (VP1, VP4 and VP6). The VP1 (149 kDa size and encoded by Seg-1) is present in the transcription complex and acts as RNA-dependent RNA polymerase (Roy et al. 1988). The VP1 also acts as BTV replicase that synthesizes dsRNA using oligo (A) primers from a viral positive-strand RNA template (Patel and Roy 2014). The VP4 and VP6 proteins are also present in transcription complex along with VP1. The VP4 protein (76 kDa size and encoded by Seg-4) is a RNA capping enzyme, also known as guanylyltransferase or transmethylase. The VP6 protein (36 kDa) is a ssRNA- and dsRNA-binding protein with helicase and NTPase activity (Van Dijk and Huismans 1988; Roy et al. 1990). The VP6 also displays RNA-dependent ATPase activity, unwinds the dsRNA and assists in mRNA synthesis from BTV dsRNA template (Roy et al. 1990).

The non-structural (NS) proteins of BTV are absent in the mature BTV virion and are found only in BTV infected cells (Van Dijk and Huismans 1988; Ratinier et al. 2011). The NS proteins contribute for the viral replication, maturation and release of viral progeny from the infected cells. The NS1 and NS2 proteins are the most expressed proteins in the infected cells. The most abundant NS1 protein forms tubules for translocation of progeny virus particles to cell membrane and release from the infected host cells. The NS1 protein is also involved in cytopathogenesis of BTV. The NS2 protein is a highly conserved ssRNA-binding protein and has nucleotidyl phosphatase activity and forms inclusion bodies in the cytoplasm of infected cells. The NS2 plays an important role in early morphogenesis, arrest spindle formation, and blocks host cell division. The NS3 is a highly conserved protein and has two isoforms namely, full-length NS3 and short truncated NS3A (lacks 13 amino acids at N-terminal end of methionine codon) (Van Dijk and Huismans 1988). The NS3 protein is the smallest non-structural membrane glycoprotein and forms viroporin which induces cytoplasmic membrane permeabilization and facilitates the release of virion particles from infected cells by budding mechanism (Hyatt et al. 1993). This probably operates in insect cells where no cytopathic effect (CPE) is induced by BTV (Schwartz-Cornil et al. 2008).

The NS4 protein is encoded by an open reading frame (ORF) in Seg-9 overlapping the ORF encoding VP6. The NS4 is expressed during early post-infection and localized in the cytoplasm and nucleoli of BTV infected cells (Belhouchet et al. 2011; Ratinier et al. 2011). The NS4 protein has a length of 77–79 amino acid residues and is highly conserved among several BTV serotypes/strains. The NS4 plays an important role in virus-host interaction and counteracts the antiviral response of the host. It modulates the host IFN responses by inhibiting the cellular transcription (Ratinier et al. 2011). Recently, NS5 protein was identified from overlapping ORF of Seg-10 (Stewart et al. 2015). The NS5 is assumed to play a synergistic role as BTV NS4 in viral nuclear localization (Stewart et al. 2015).

## 4. BTV serotypes

Globally, 28 distinct BTV serotypes have been reported as of now by VI and serological assays (Maan et al. 2011; Schulz et al. 2016; Bumbarov et al. 2020). Any of these serotypes have the potential to cause BT in ruminants. Variations in sequence of genome Seg-2 and its translated protein VP2 determines the serotypes and also partially by Seg-6 and its translated protein VP5. In contrast to already existing 28 serotypes of BTV, BTV-25, -26 and -27 were reported to be non-pathogenic, direct contact transmission (also BTV-28), unable to culture in *Culicoides* cell lines, found exclusively in small ruminants, and regarded as ‘atypical’ serotypes (Maan et al. 2011; Batten et al. 2013; Schulz et al. 2016; Bumbarov et al. 2020). The BTV-25 was isolated from asymptomatic goats at Toggenburg in Switzerland in 2007 (Chaignat et al. 2009). The BTV-26 was isolated from clinical samples of sheep in Kuwait (Maan et al. 2011). The BTV serotype-27 was recently isolated from asymptomatic goats in Corsica, France in 2014 (Schulz et al. 2016).

The BTV-28 was detected from contaminated vaccine batches of lumpy skin disease and sheeppox vaccine in Israel, Middle East (Bumbarov et al. 2020). The BTV-28 was found to spread by in-contact transmission and causes clinical disease. Phylogenetic analysis of Seg-2 of BTV-28 showed that it was related to BTV-4, -10, -11, -17, -20, and -24. The Seg-5 of BTV-28 is closely similar to South African BTV-4 strain, and other segments are closely related with BTV-26. Experimental infection of BTV-28 in ewes showed typical clinical signs of BT (Schulz et al. 2016; Bumbarov et al. 2020). Recently, three novel putative BTV serotypes were identified (Sun et al. 2016; Savini et al. 2017). The first novel putative BTV was isolated from samples of an Alpaca in South Africa and phylogenetic and cross-neutralization analysis revealed close similarity with BTV-15. Second putative novel BTV (BTV-X ITL2015) was detected from healthy goats in Sardinia, Italy and so far isolation was unsuccessful (Savini et al. 2017). The Seg 2 of BTV-X ITL2015 showed more identity with BTV-27 isolated from Corsica and with recently isolated BTV (XJ1407) from China. Third putative BTV serotype (XJ1407) was isolated from goats and detected in sheep in China (Sun et al. 2016).

BTV serotypes are distributed globally and it depends on the availability of susceptible host and vector *Culicoides* populations within the geographical regions (St George et al. 1978). The BTV strains within the same serotype revealed 27.4% of amino acid and 31.6% of nucleotide differences in Seg-2/VP2 (Maan et al. 2010; Maan, Maan, Nomikou, Guimera, et al. 2012). Different serotypes of BTV showed 22.2% of amino acid and 26.8% nucleotide similarity resulted in distinct identification and differentiation of BTV serotypes are difficult (Maan et al. 2010). Even within the same serotype, huge phenotypic and genotypic differences were noticed due to variations in the nucleotide sequence of BTV strains, which correlate with their geographical regions/origins known as ‘BTV topotypes’ and can be subdivided into groups based on their origin (Gould and Pritchard 1990; Bonneau et  al. 2001; Maan, Maan, Nomikou, Guimera, et al. 2012; Maan, Maan, Guimera, Nomikou, Singh, et al. 2012; Maan, Maan, Pullinger, et al. 2012; Shaw et al. 2013). Most of the genome segments of BTV can clearly segregate into ‘eastern’ [BTV from Asia (Indian subcontinent, East Asia-China, Japan, Taiwan; Southeast Asia-Indonesia), Australia, Europe, Middle East (Turkey), and Mediterranean Basin] and ‘western’ [African continent (South Africa and Nigeria), the United States (Brazil and Guatemala), Caribbean region (Jamaica and Caribbean islands)] topotypes/groups based on their geographic origin (Maan et al. 2010, 2011; Mann, Maan, Nomikou, Guimera, et al. 2012). This indicated that BTV strains have evolved over a long period of time with little genetic exchange between regions and multiple point mutations, allowing them to acquire clear regional differences (Maan et al. 2010).

BTV replication is highly error-prone due to lack of proof-reading mechanisms like other RNA viruses (Bonneau et al. 2001). The random mutation and/or reassortment of genome segments of BTV strains resulted in variability between the BTV strains (Maan et al. 2010; Maan, Maan, Nomikou, Guimera, et al. 2012; Shaw et al. 2013). This may result in emergence of novel BTV strains with increased virulence (Waldvogel et al. 1987) or increased abilities to adapt into new geographical zones or re-entry into endemic areas through anthropogenic or natural routes (Bonneau et al. 2001; Maan et al. 2010; Maan, Maan, Nomikou, Guimera, et al. 2012; Maan et al. 2015; Schirtzinger et al. 2018). Continuous screening of different serotypes and topotypes of BTV are important for epidemiological monitoring and important for the effective implementation of control and eradication strategies including vaccine matching (van Rijn 2019).

## 5. Global epidemiology of BTV

BT was first reported in the late eighteenth-century in African continent (Spreull 1905), since then, the disease has been reported in different continents/countries including South and North American continents, Australia, Europe, and Asia including the Indian subcontinent (St George et al. 1978; Wilson and Mellor 2009; Sperlova and Zendulkova 2011; MacLachlan and Mayo 2013; Ranjan et al. 2015; Rao et al. 2016; Sun et al. 2016; Bumbarov et al. 2020). At present, the disease is present in almost all continents except Antarctica (Gould and Pritchard 1990). Prevalence of various BTV serotypes worldwide are elaborated in Table 1. The complex epidemiology of BTV can be influenced by density and distribution of *Culicoides* vector population and host species composition, climatic conditions and virus strains. BT outbreaks occur in tropical, subtropical and temperate regions (lies between latitudes 35°S and 40°N) of the world that favours the breeding of competent vector species. More than one or two serotypes are recorded during an outbreak in every specific season reflecting the dynamic changes in BTV serotypes and herd immunity (Bommineni et al. 2008; Shafiq et al. 2013; Hemadri et al. 2017; Reddy et al. 2018).

**Table 1.** Prevalence of various bluetongue virus (BTV) serotypes worldwide.

Geographical distribution	Prevalent serotypes of BTV*
African continent (South Africa, Egypt, Algeria, Libya, Morocco, Tunisia, and Nigeria	1, 2, 3, 4, 5, 6, 7, 8, 9, 10, 11, 12, 13, 14, 15, 16, 17, 18, 19, 20, 22, 24
European continent (France, the Netherlands, Germany, Belgium, Spain, Portugal, Switzerland, Ireland, Luxembourg)	1, 2, 4, 6, 8, 9, 10, 11, 14, 16, 25, 27
North American continent (USA, Mexico, Canada)	1, 2, 3, 5, 6, 9, 10, 11, 12, 13, 14, 17, 18, 19, 22, 24
South American continent (Brazil, French Guiana, Argentina, Colombia, Suriname, Guyana, and Ecuador)	1, 2, 3, 4, 6, 8, 9, 10, 12, 13, 14, 17, 18, 19, 20, 21, 22, 24, 26
Central America (Guatemala) and Caribbean region (Jamaica and Caribbean islands)	1, 3, 4, 6, 8, 10, 11, 12, 13, 14, 17, 19, 22
Australian continent	1, 2, 3, 4, 5, 7, 9, 12, 15, 16, 20, 21, 23, 24
South Asia (India, Pakistan, Sri Lanka, Bangladesh, Afghanistan)	1, 2, 3, 4, 5, 6, 7, 8, 9, 10, 11, 12, 13, 14, 15, 16, 17, 18, 19, 20, 21, 23, 24
East Asia (China, Japan and Taiwan)	1, 2, 3, 4, 5, 6, 7, 8, 9, 10, 11, 12, 13, 15, 16, 20, 21, 23, 24, 28
Southeast Asia (Indonesia and Malaysia)	1, 2, 3, 5, 6, 7, 9, 12, 15, 16, 20, 21, 23
Western Asia (Turkey, Cyprus, Syria, Lebanon, Israel, Jordan, Oman, Kuwait, Saudi Arabia)	1, 2, 3, 4, 5, 6, 8, 10, 12, 15, 16, 24, 26, 28

* Reported by virus isolation and presence of neutralising antibodies.

Updated from Barber (1979);Taylor and Mellor (1994); Tabachnick (2004); Wilson and Mellor (2009); Maan et al. (2011); Sperlova and Zendulkova (2011); Coetzee et al. (2012); Sun et al. (2016); Bumbarov et al. (2020).

BT was endemic in Africa, west Asia, Europe and Indian Subcontinent countries. The BTV is endemic in South Africa and 22 out of 28 known serotypes (except BTV-20, -21, -25, -26, -27, and -28) have been reported (Coetzee et al. 2012). The BTV-1–6, -8, -10, -11 and -24 serotypes were most commonly reported and more often associated with clinical disease with high pathogenic potential in sheep, and have high epidemic potential in South Africa. Every season, BTV-9, -10, -12, -13, -16, and -19 serotypes were reported with much lower frequency and BTV-7, -15, and -18 serotypes were reported sporadically. The BTV-1–6 and -10 serotypes are more often associated with clinical disease associated with high pathogenic potential in sheep (Gerdes 2004; Coetzee et al. 2012). In north African countries, Egypt was found to have BTV-1, -4, -10, and -12; whereas, BTV-1, -2, and -4 were isolated from Libya, Algeria, Tunisia, and Morocco since 2002.

The BTV-2, -3, -4, -6, -8, -9, -11, -15, -16, and -18 serotypes have been reported from sheep flocks of Pakistan (Sarwar 1962; Akhtar et al. 1995). The seroprevalence of BTV in Pakistan was 18%–57% in sheep, 41%–51% in goat, 18% in cattle, and 29% in buffalo (Sohail et al. 2018). In China, at least 14 BTV serotypes (BTV-1–5, -7, -9, -11, -12, -15, -16, -21, -23, and -24) have been reported by serological testing and VI. The BTV-1, -2, -4, and -16 are most prevalent serotypes in China and causes clinical disease in sheep (Sun et al. 2016; Yang et al. 2017). In southeast Asia (Indonesia and Malaysia), BTV-1, -2, -3, -7, -9, -12, -16, -21, and -23 serotypes have been isolated from cattle and *Culicoides* spp. in Malaysia, BTV-1, -2, -3, -9, -16, and -23 serotypes were isolated from cattle and appeared to be endemic (Daniels et al. 2004; Pritchard et al. 2004). Among west Asian countries, BTV-2, -4, -6, -10, and -16 are endemic in Israel, and BTV-5, -8, -12, -15, and -24 are recently reported (Sperlova and Zendulkova 2011; MacLachlan and Mayo 2013). In Lebanon, BTV-1, -4, -6, -8, -16, and -24 were reported in 2011. BT has also been reported from Middle East countries like Iran, Iraq, Oman, Jordan, Saudi Arabia, Kuwait, etc (Maan et al. 2011).

A sporadic outbreak of BTV-10 was reported till 1998 in Europe (Wilson and Mellor 2009). Eleven different serotypes BTV-1, -2, -4, -6, -8, -9, -11, -14, -16, -25, and -27 has been reported to be prevalent in Europe since 1998 (Wilson and Mellor 2009). Europe (Germany and the Netherlands) faced severe BTV-8 outbreaks in sheep, cattle and goats during 2006, and later in France during 2015 (Dercksen et al. 2007; Wilson and Mellor 2009; Conraths et al. 2009; Schulz et al. 2016). During 2008, BTV-6 was reported in the Netherlands and Germany, and BTV-11 from Belgium (Wilson and Mellor 2009). In Germany, BTV had not been reported before 2006. The BTV-8 outbreak caused more than 24,000 cases from August 2006 to August 2008 and most of the cases were reported in 2007 (20,635 cases) and 1,070 cases in 2008 (Conraths et al. 2009). In 2006, BTV-8 was diagnosed on 571 cattle farms, 309 sheep flocks, 6 red deer, 3 mouflons, and 1 roe deer in Germany. During 2006, cattle (48,364 exposed to BTV/1,131 infected/72 died), sheep (9,781 exposed/590 infected/221 died) and goats (56 exposed) were affected in Germany (Conraths et al. 2009). During 2007, BT was detected on 12,638 cattle farms, 23 other individual bovines, 7,790 sheep flocks, 115 goat herds, 34 fallow deer, 11 mouflons, 10 red deer, and 3 roe deer. In 2007, cattle (1,317,111 exposed to BTV/26,772 infected), sheep (503,282 exposed/32,116 infected) and goats (3,346 exposed/209 infected) were affected in Germany (Conraths et al. 2009).

In Australia, twelve BTV serotypes (BTV-1–3, -5, -7, -9, -12, -15, -16, -20, -21, and -23) have been recorded in Australia (Firth et al. 2017). Phylogenetic analyses showed most of the Australian serotypes are eastern topotypes thought to have migrated from Asia (St George et al. 1978; Maan, Maan, Nomikou, Guimera, et al. 2012; Maan, Maan, Nomikou, Prasad, et al. 2012; Krishnajyothi et al. 2016; Firth et al. 2017). Despite the presence of BTV from many years (1975 onwards) and several serotypes in Australia, still no established clinical BT disease outbreaks have been reported in commercial sheep populations due to very limited and/or sporadic availability of vector (*C. brevitarsis*) in south Australia, where the majority of the sheep grazing areas are located (Firth et al. 2017).

Serological evidence suggested the existence of BTV in the majority of the Southand North American continents, except Alaska, southern parts of Pampas and Patagonia (Tabachnick 2004; Sperlova and Zendulkova 2011; MacLachlan and Mayo 2013). In South American continent, BTV prevalence was reported in various countries like BTV-1, -2, -3, -4, -6, -9, -12, -14, -17, -18, -19, -20, -21, -22, and -26 from Brazil; BTV-4 from Argentina; BTV-12, -14, and -17 from Colombia; BTV-6, -14, and -17 from Suriname; BTV-1, -2, -6, -10, -12, -13, -17, and -24 from French Guiana; BTV-9, -13, and -18 from Ecuador; and BTV-14 and -17 from Guyana (Wilson et al. 2008; Sperlova and Zendulkova 2011; MacLachlan and Mayo 2013; da Silva et al. 2018).

In North American continent, especially in the United States and Mexico BTV-1, -2, -10, -11, -12, -13, and -17 were reported to be endemic (Johnson et al. 2006). In Florida, BTV-2, -3, -5, -6, -9, -12, -14, -18, -19, -22, and -24 were isolated (Johnson et al. 2006; Schirtzinger et al. 2018). The BTV-11 has been isolated from cattle with mild clinical disease in the British Columbia, Canada. *Culicoides sonorensis* is considered as the principal vector of BTV in North America, especially in the United States and *C. insignis* also plays role in southeastern the United States. In Central America and Caribbean Basin, BTV-1, -3, -4, -6, -8, -12, and -17 serotypes are endemic; however, other serotypes BTV-10, -11, -13, -14, -19, and -22 are also reported (Schirtzinger et al. 2018). *Culicoides insignis* is considered as main vector for BTV in Caribbean basin, Central and South America.

## 6. BTV scenario in India

BT causes great economic impact on the livestock sector of the Indian subcontinent. The Indian subcontinent lies between 8.4°N and 37.6°N, and 68.7°E and 97.25°E (Maheshwari 2012; Rao et al. 2016). BT is endemic in India and BTV outbreaks were mostly reported in crossbreeds and exotic breeds of sheep. However, in south India most of the outbreaks were reported in native breeds of sheep. India is one of the major sources of BTV in Asia due to the vast animal populations. Since the first report of BT from India in 1964, 23 serotypes (except 22, 25–28) of BTV have been reported from India by serological assays and/or VI. So far, fifteen serotypes (BTV-1–6, 9, 10, 12, 16-18, 21, 23, and 24) were identified by VI, while 22 serotypes (BTV-1–20, 23, and 24) were reported by serological testing. Most of the serotypes were isolated from south Indian states (Mehrotra et al. 1989, 1995, 1996; Prasad et al. 1992, 1994; Sreenivasulu et al. 1999, 2004; Biswas et al. 2010; Maan, Maan, Guimera, Pullinger, et al. 2012; Maan, Maan, Nomikou, Guimera, et al. 2012; Maan, Maan, Nomikou, Prasad, et al. 2012; Maan, Maan, Pullinger, et al. 2012; Maan et al. 2017; Susmitha et al. 2012; Chauhan et al. 2014; Ranjan et al. 2015; Krishnajyothi et al. 2016; Rao et al. 2016; Hemadri et al. 2017). BT outbreaks in different parts of India are coincident with density and distribution of ruminant population, *Culicoides* vectors, rainfall and climatic conditions (Rao et al. 2016). Prevalence of various BTV serotypes in India are elaborated in Table 2.

**Table 2.** Prevalence of bluetongue virus (BTV) serotypes in different states of India.

Indian States	BTV serotypes based on the virus isolation	BTV serotypes based on the neutralizing antibodies
Andhra Pradesh	1, 2, 4, 9, 10, 12, 16, 21, 24	2, 4, 6, 7, 8, 9, 12, 13, 14, 17, 18, 19
Tamil Nadu	1, 2, 3, 16, 18, 23	1, 3, 4, 5, 6, 7, 11, 12, 13, 14, 15, 16, 17, 19, 20
Karnataka	1, 2, 5, 16, 18, 23	1, 2, 4, 12, 16, 17, 20
Maharashtra	1, 2, 3, 4, 9, 16, 17, 18, 23	1, 2, 3, 4, 5, 6, 7, 8, 9, 10, 12, 13, 16, 17
Gujarat^#^	1, 6, 16	1, 2, 3, 4, 5, 6, 8, 9, 10, 11, 12, 13, 14, 15, 16, 17, 18, 19, 20, 23, 24
Haryana	1, 4	1, 2, 3, 5, 8, 9, 10, 11, 12, 13, 15, 16, 17, 20
Madhya Pradesh	1, 18, 23	2, 7, 9, 10, 18, 23
Uttar Pradesh	1, 9, 18, 23	23
Uttarakhand	1, 23	—
Himachal Pradesh	3, 4, 9, 16, 17	1, 4, 5, 15, 17
Jammu and Kashmir	18, 23	1, 2, 8, 12, 16
Rajasthan	1	1
West Bengal*	3, 15, 21	—
Odisha	1	—

Updated from Mehrotra et al. (1989, 1995, 1996); Prasad et al. (1992, 1994); Sreenivasulu et al. (1999, 2004); ^#^Chandel et al. (2005); Chauhan et al. (2005); Bommineni et al. (2008); Biswas et al. (2010); Tembhurne et al. (2010); Maan, Maan, Guimera, Nomikou, Singh, et al. (2012); Maan, Maan, Guimera, Pullinger, et al. (2012); Maan, Maan, Nomikou, Guimera, et al. (2012); Maan, Maan, Nomikou, Prasad, et al. (2012); Maan, Maan, Pullinger, et al. (2012); Maan et al. (2015); Minakshi et al. (2012); Susmitha et al. (2012); Shafiq et al. (2013); Chauhan et al. (2014); Ranjan et al. (2015); Krishnajyothi et al. (2016); Rao, Reddy, and Hegde (2012); Rao, Reddy, Meena, et al. (2012); Rao et al. (2016); Dalal et al. (2017); Hemadri et al. (2017); Reddy et al. (2018); *Joardar et al. (2009) serotyped BTV-15 by serum neutralisation test, but the serotyping has now been refuted (Rao et al. 2016).

### 6.1. Climatic conditions of India

The tropic of cancer (TOC) divides India into two zones namely subtropical and tropical zones. The subtropical zones include northern parts of India namely, Rajasthan (TOC passes only through Banswara district), Uttar Pradesh, Bihar, Haryana, Punjab, Uttarakhand, Himachal Pradesh, Jammu and Kashmir, and Union Territories like Delhi and Chandigarh. Further, the most highest and massive Himalayan Mountains prevents the influx of frigid katabatic winds from the northern Central Asia and icy Tibetan Plateau (De et al. 2005; Rao et al. 2016). Hence, the climatic conditions in most of the northern parts of India are harsh like very hot (up to 50 °C) during summer (first week of April to last week of June) and more cold (up to 0 °C) during winter (last week of October to first week of March) than other regions of the globe with similar latitudes. The Himalayan Mountains in India range from the parts of Jammu and Kashmir, Himachal Pradesh, Uttarakhand, Sikkim, Arunachal Pradesh, Nagaland, Manipur, Mizoram, Tripura, and Meghalaya States (De et al. 2005; Rao et al. 2016).

The tropical zone includes southern parts of India namely, Tamil Nadu, Kerala, Andhra Pradesh, Karnataka, Telangana, Goa, and Puducherry (Union Territory). The southern parts of India are peninsula (surrounded by water bodies on three sides namely, Arabian Sea in the west, Bay of Bengal in the east and Indian Ocean in the south) and the Torrid Zone (lies close to the equator), which are the zone of dry (interior regions) or humid (coastal regions) and hottest during summer, and dry winters than the rest of the country (De et al. 2005; Rao et al. 2016). Hence, climatic conditions in most of the southern parts of India are very hot (32–50 °C) during summer (first week of March to last week of May), and during winter (last week of October to last week of February), 10–16 °C in the night and 21–30 °C in the daytime except in Tamil Nadu, where the northeast monsoon brings bout of rains, hence temperature fluctuates. However, maritime coastal winds and long mountain ranges of Western and Eastern Ghats play a major role in relatively invariant temperature pattern (Rao et al. 2016; Benelli et al. 2017).

Overall, India has a tropical climate with hot and humid, which depends on monsoon rainfall (De et al. 2005; Rao et al. 2016). Monsoon starts in southern region during late May and/or early June and proceeds to northern region. Thus, June to October are the months of rain bearing southwest monsoon that benefits all parts of India, which is favourable for *Culicoides* and occurrence of BT (Ranjan et al. 2015; Rao et al. 2016; Reddy et al. 2016). The northeast monsoon occurs from October to late March, which contributes to significant rainfall in southern states of India, which favours the *Culicoides* vectors to transmit the BTV (Rao et al. 2016).

### 6.2. Livestock population in India

Livestock rearing is the main source of income in India. India has more than 223 million sheep and goat population. According to 20^th^ livestock census (2019), India has 192.49 million cattle, 109.85 million buffalo, 74.26 million sheep, and 148.88 million goat population. The sheep population is not uniform in entire India. Among the different states of India, Telangana (19.1 million), Andhra Pradesh (17.6 million), Karnataka (11.1 million), Rajasthan (7.9 million), and Tamil Nadu (4.5 million) have the largest sheep population; whereas, Jammu and Kashmir (3.2 million), Maharashtra (2.7 million), Gujarat (1.8 million), Odisha (1.3 million), and Uttar Pradesh (1.0 million) have the lowest sheep population (DAHD&F 2019).

### 6.3. Factors determining outbreaks of BT in India

The disproportionate sheep density in different regions leads to disparity in occurrence of BT frequency in India. Both sheep density and conducive climate for *Culicoides* are required for the BTV outbreak, which was obviously correlated with least outbreaks in Kerala and Odisha having suitable climate but less densely populated sheep. Likewise, Rajasthan has high sheep population but dry climatic conditions unfavorable for the BTV occurrences (Maheshwari 2012; Rao et al. 2016; Benelli et al. 2017). Breed susceptibility to BTV infection are less reported in India (Ranjan et al. 2015; Rao et al. 2016). Wool type sheep breeds favour the outbreak of BT in India. Fine wool/hairy breeds are more susceptible than carpet/coarse wool breeds due to the fact that vector bites are common in fine wool breeds (Rao et al. 2016). Hairy type Nellore breed is highly susceptible than Deccani breed in northern Andhra Pradesh (Susmitha et al. 2012; Rao et al. 2016). Meat purpose sheep breeds namely, Vembur and Mecheri are less susceptible than Ramnad white (meat purpose) and Trichy black (wool purpose) breeds in Tamil Nadu (Prasad et al. 1992; Ilango 2006; Rao et al. 2016; Reddy et al. 2016). The differences in BT susceptibility among these breeds may be due to genetic as well as hair coat differences (Prasad et al. 1992; Ilango 2006; Rao et al. 2016).

In India, BT is widely spread in Andhra Pradesh, Telangana, Tamil Nadu, Karnataka, Gujarat, Jammu and Kashmir, and Haryana, and many outbreaks of BTV with severe clinical manifestations were reported since 1964. The occurrence of BT in sheep is more severe in south Indian states and is less severe in north Indian states (Ilango 2006; Rao et al. 2016; Reddy et al. 2016). The clinical manifestations of BT in sheep in north India was slightly different from south India. The main differences in the clinical manifestations are swelling of face and lips were less obvious, though mucocutaneous borders are very sensitive to touch and bled easily upon handling. The classical signs of BT like cyanosis of the tongue and reddening of the coronary band are rarely seen in either north or south Indian sheep breeds.

### 6.4. Seroprevalence of BTV in India

**Figure 1.**
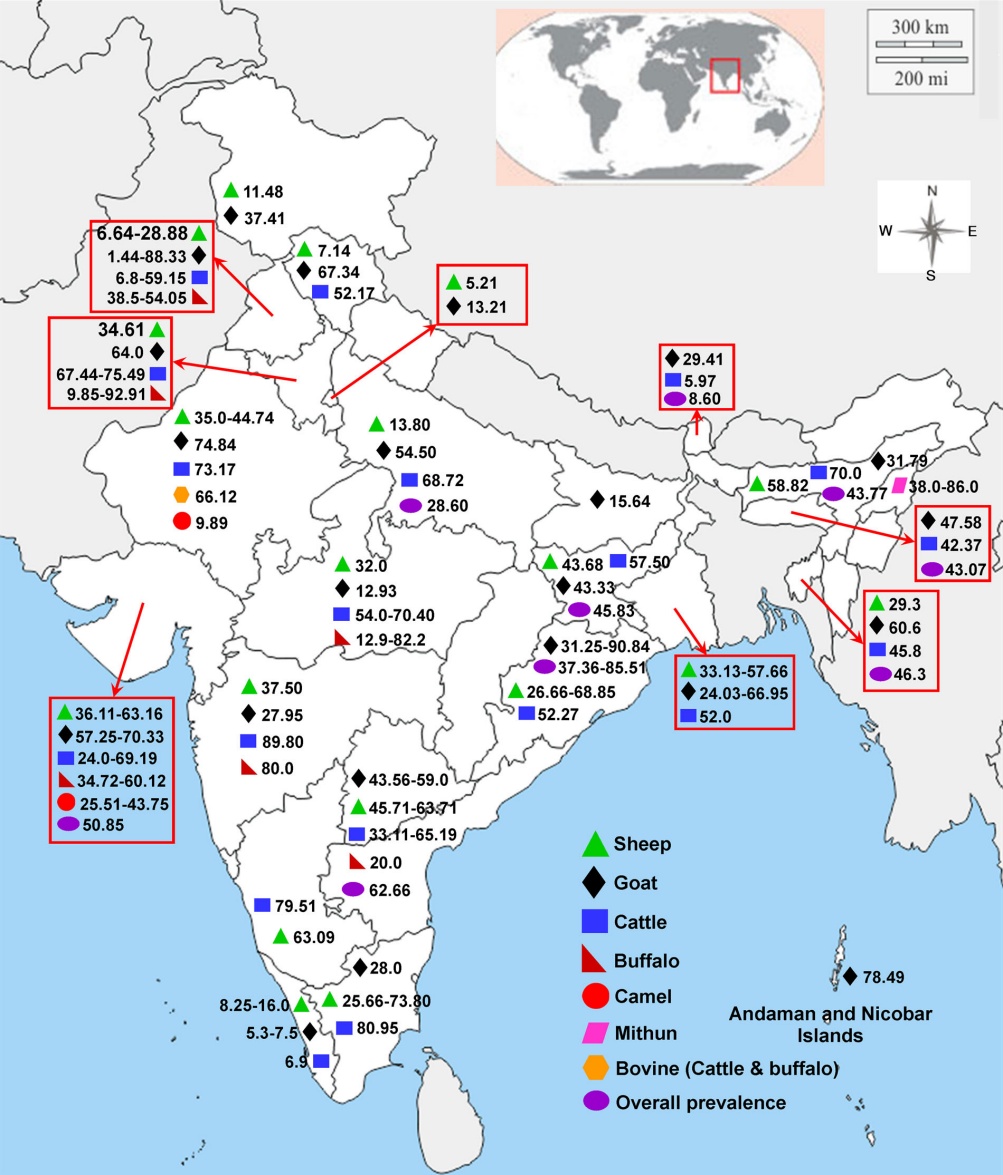
Seroprevalence of bluetongue virus in different States of India among various species of animals. The seroprevalence data was expressed as percentage. Seroprevalence data for Telangana and Andhra Pradesh States were mentioned together. The references for mentioned data were available in Table 3.

The seroprevalence survey of BTV reported highest prevalence in Andhra Pradesh, among southern states as compared to Karnataka and Tamil Nadu (Ilango 2006; Susmitha et al. 2012; Rao et al. 2016). Wide serological screening of BTV has been carried out in different states of India over a period of time (1980–2019) among various species of animals elaborated in Table 3 and Figure 1.

**Table 3.** Details of seroprevalence of bluetongue virus in different States or Union Territories of India over a period of time among various species of animals. Andaman and Nicobar Island (UT)

Sampling period	States or Union Territories of India	Seroprevalence (%)	Total no. of samples collected/no. of samples tested positive	Animal species	Diagnostic test	References
1980	Punjab	6.64	346/23	Sheep	AGPT	Sodhi et al. (1981)
		1.44	140/2	Goat		
1980	Punjab	6.8	528/36	Cattle	AGPT	Sharma (1981)
		0	133/0	Buffalo		
1987	Punjab	38.5	NA	Buffalo	NA	Oberoi et al. (1988)
2015	Punjab	8.7	92/8	Bovine (cattle and buffalo	c-ELISA	Sharma et al. (2016)
1981	Maharashtra	37.5	NA	Sheep	NA	Harbola et al. (1982)
2007	Maharashtra	27.95	651/182	Goat	c-ELISA	Ingle et al. (2008)
2011	Maharashtra	89.80	206/185	Cattle	c-ELISA	Raut et al. (2013)
		80.00	40/32	Buffalo		
1982	Gujarat	13.4	NA	Buffalo	NA	Tongaonkar et al. (1983)
		15.6	NA	Cattle		
1998	Gujarat	9.33	150/14	Camel	AGID	Chandel and Kher (1999)
1998–1999	Gujarat	18.75	48/9	Aborted Cattle	AGID	Chandel et al. (2001)
		9.59	73/7	Clinically healthy cattle		
		29.63	27/8	Aborted buffalo		
		13.95	43/6	Clinically healthy Buffalo		
		8.12	160/13	Clinically healthy sheep		
		36.84	38/14	Aborted goats		
		20.0	50/10	Clinically healthy goats		
		43.75	48/21	Clinically healthy Camels		
		18.06	487/88	Overall seroprevalence		
1999	Gujarat	63.16	NA	Sheep	c-ELISA	Hinsu et al. (2000)
2001–2002	Gujarat	12.5	176/22	Camel	AGID	Chandel et al. (2003)
		19.3	176/34		c-ELISA	
1993–2003	Gujarat	24.66	908/224	Sheep	AGPT	Chandel et al. (2004, 2005)
		29.15	199/58	Goat		
		24.0	150/36	Cattle		
		34.72	216/75	Buffalo		
		9.33	150/14	Camel		
		25.07	1623/407	Overall seroprevalence		
2003	Gujarat	36.11	NA	Sheep	c-ELISA	Chauhan et al. (2004)
		70.33		Goats		
		69.19		Cattle		
		58.33		Buffalo		
		38.34		Camel		
		50.85	1410/717	Overall seroprevalence		
		22.69	NA	Sheep	AGID	
		50.92		Goats		
		48.82		Cattle		
		39.29		Buffalo		
		26.69		Camel		
		34.96	1410/493	Overall seroprevalence		
2003	Gujarat	26.69	326/87	Camel	AGID	Harshad et al. (2004)
		38.34	326/125		c-ELISA	
2004	Gujarat	39.29	168/66	Buffalo	AGID	Chauhan et al. (2005)
		58.33	168/98		c-ELISA	
2006	Gujarat	35.26	173/61	Buffalo	AGID	Patel et al. (2007)
		39.88	173/69		CCIE	
		60.12	173/104		c-ELISA	
2012–2013	Gujarat	57.25	510/292	Goat	c-ELISA	Bhagat et al. (2014)
		39.61	510/202		i-ELISA	
		26.47	510/140		AGID	
2016	Gujarat	39.26	382/150	Sheep	c-ELISA	Patel et al. (2017)
		34.03	382/130		i-ELISA	
2016	Gujarat	15.57	533/83	Camel	AGID	Shah et al. (2017)
		25.51	533/136		c-ELISA	
1978–1984	Rajasthan	35.0	400/140	Sheep	AGPT	Sharma et al. (1985)
NA	Rajasthan	36.0	NA	Sheep	AGPT	Dubey et al. (1987)
		74.84	NA	Goat		
		66.12	NA	Bovine		
NA	Rajasthan	9.89	182/18	Camel	AGID	Malik et al. (2002)
2003–2004	Rajasthan	36.02	483/174	Sheep	c-ELISA	Sonawane et al. (2008)
		74.84	485/363	Goats		
1990	Haryana	9.85	477/47	Buffalo	AGID	Jain et al. (1992)
		3.91	537/21	Cattle		
		4.66	257/12	Bovine cow		
		6.25	96/6	Bovine bulls		
		1.61	186/3	Bovine calf		
		11.92	285/34	Adult she-buffalo		
		13.48	89/12	Buffalo bulls		
		0.97	103/1	Buffalo calves (< 1 year)		
		13.66	183/25	Buffalo	DIA	
		10.31	223/33	Cattle		
1994	Haryana	34.61	NA	Sheep	c-ELISA	Naresh and Prasad (1995)
		64.0		Goat		
		67.44		Cattle		
	Himachal Pradesh	7.14	NA	Sheep		
		67.34		Goat		
		52.17		Cattle		
	Punjab	28.88	NA	Sheep		
		88.33		Goat		
		59.15		Cattle		
		54.05		Buffalo		
2014	Haryana	75.49	408/308	Cattle	c-ELISA	Maan et al. (2017)
		92.91	395/367	Buffalo		
November 2007 to March 2008	Jammu province	37.41	139/52	Goat	i-ELISA	Singh et al. (2009)
		11.48	61/7	Sheep		
2007	Madhya Pradesh	70.4	277/195	Cattle	c-ELISA	Kumari et al. (2010)
		82.2	118/97	Buffalo		
2014	Madhya Pradesh	32.0	50/16	Sheep	c-ELISA	Varsha (2015)
		12.93	348/45	Goat		
		32.76	235/77	Cattle	i-ELISA	
		54.0	100/54	Cattle	c-ELISA	
		12.9	124/16	Buffalo		
September and November 2012	Uttar Pradesh	13.8	58/8	Sheep	c-ELISA	Bitew et al. (2013)
		54.5	33/18	Goat		
		28.6	91/26	Overall seroprevalence		
October 2001 to July 2002	Kerala	8.25	109/9	Sheep	dot ELISA and c-ELISA	Ravishankar et al. (2005)
		5.3	901/48	Goat		
2012	Kerala	16	50/8	Sheep	c-ELISA	Arun et al. (2014)
		7.5	40/3	Goat		
		6.9	82/5	Cattle		
NA	Tamil Nadu	73.80	NA	Sheep	c-ELISA	Malmurugan et al. (2008)
November 2004 to January 2005	Tamil Nadu	28.0	150/42	Sheep and Goat	AGPT	Ramesh et al. (2009)
June 2007 to May 2009	Tamil Nadu	25.66	600/154	Sheep	i-ELISA	Selvaraju and Balasubramaniam (2013)
1998	Andhra Pradesh and Telangana	45.71	976	Sheep	c-ELISA	Sreenivasulu and Rao (1999)
		43.56		Goat		
		33.11		Cattle		
		20.0		Buffalo		
August to December 2014	Andhra Pradesh	63.71	350/223	Sheep	c- ELISA	Didugu et al. (2015)
		59.0	100/59	Goat		
		62.66	450/282	Overall seroprevalence		
2001	Andhra Pradesh	65.19	NA	Cattle	c-ELISA	Dayakar et al. (2001)
	Karnataka	79.51				
	Tamil Nadu	80.95				
2013–2014	Jharkhand	43.68	190/83	Sheep	i-ELISA	Tigga et al. (2015)
		43.33	210/91	Goat		
		57.50	80/46	Cattle		
		45.83	480/220	Overall seroprevalence		
February 2013 to January 2016	Middle Indo-Gangetic plains of Bihar	15.64	504/79	Goat	c-ELISA	Kumar et al. (2018)
2013	Odisha	26.66	120/32	Sheep	i-ELISA	Joardar et al. (2014)
		31.25	112/35	Goat		
		52.27	132/69	Cattle		
		37.36	364/136	Overall seroprevalence		
October 2015 to April 2016	Odisha	60.36	217/131	Sheep	i-ELISA	Hota et al. (2017)
October 2011 to March 2012	Odisha	68.85	122/84	Sheep	i-ELISA	Pany et al. (2018)
		90.84	382/347	Goat		
		85.51	504/431	Overall seroprevalence		
2006	West Bengal	34.47	NA	Sheep	i-ELISA	Chakrabarti et al. (2007)
		24.03		Goat		
Winter months of 2008	West Bengal	47.0	1202/565	Goat	i-ELISA	De et al. (2009)
2010	West Bengal	66.95	115/77	Goat	i-ELISA	Panda et al. (2011)
		57.66	137/79	Sheep		
		52.0	50/26	Cattle		
July to December, from 2010 to 2013	West Bengal	33.13	504/167	Sheep	i-ELISA	Halder et al. (2016)
		30.24	1005/304	Goat		
2016	Sikkim	5.97	134/8	Cattle	i-ELISA	Ramudamu et al. (2017)
		29.41	17/5	Goat		
		8.60	151/13	Overall seroprevalence		
2011	Assam	58.82	68/40	Sheep	i-ELISA	Joardar et al. (2013)
		31.79	195/62	Goat		
		70.00	50/35	Cattle		
		43.77	313/137	Overall seroprevalence		
2015	Tripura	43.88	136/59	Goat	i-ELISA	Joardar et al. (2016)
		42.37	59/25	Cattle		
		43.07	195/13	Overall seroprevalence		
2014–2017	Tripura	47.58	1240/590	Goat	i-ELISA	De et al. (2019)
2005	Nagaland	86.0	172/148	Mithun	c-ELISA	Rajkhowa et al. (2008)
2014	Nagaland	38.0	50/19	Mithun	i-ELISA	Joardar et al. (2015)
2016	Meghalaya	29.3	147/43	Sheep	i-ELISA	Nongdhar et al. (2017)
		60.6	188/114	Goat		
		45.8	367/168	Cattle		
		46.30	702/325	Overall seroprevalence		
August 2017–February 2018	Meghalaya	60.20	598/360	Goat	c-ELISA	Karam et al. (2018)
2005–2006	Delhi (UT)	5.21	192/10	Sheep	AGID	Audarya et al. (2014)
2005–2006	Delhi (UT)	13.21	469/62	Goat	AGID	Audarya et al. (2015)
2016		78.49	186/146	Goat	i-ELISA	Inbaraj et al. (2019)
1982	Haryana, Uttar Pradesh, Rajasthan and Andhra Pradesh	3.0	NA	Goat	NA	Bandopadhyay and Mullick (1983)
		3.7	NA	Cattle		
1983	Maharashtra, Andhra Pradesh, Karnataka, Jammu and Kashmir and Himachal Pradesh	16.4–61.1	NA	Sheep	NA	Mehrotra and Shukla (1984)
1986	Haryana and Rajasthan	29.2	356/104	Sheep	AGPT	Prasad et al. (1987)
		3.48	460/16	Cattle		
		0	45/0	Goat		
		0	60/0	Buffalo		
		0	128/0	Camel		
		0	128/0	Horse		
2006	Uttar Pradesh, Jammu and Kashmir, Maharashtra, Rajasthan and Gujarat	37.9	516/196	Sheep	AGID	Bhanuprakash et al. (2008)
		56.8	516/293		i‐ELISA	
		53.5	516/276		c‐ELISA	
2013–2014	Rajasthan, Uttar Pradesh, and Karnataka	73.08	416/304	Cattle	c-ELISA	Ayanur et al. (2016)
		53.30	160/87	Sheep		

AGPT, agar gel precipitation test; AGID, agar gel immunodiffusion assay; c-ELISA, competitive enzyme linked immunosorbent assay; DIA, dot immuno-binding assay; i-ELISA, indirect enzyme linked immunosorbent assay; NA, data not available.

### 6.5. Isolation of BTVs from India

Bhambani and Singh (1968) isolated the BTV from an outbreak in government livestock farm in sheep of Uttar Pradesh and experimentally reproduced the disease in sheep; however, isolated BTV serotype did not describe. The BTV-2 and BTV-16 were first isolated from Maharashtra in 1973 (Prasad et al. 1994), then BTV-2 was isolated from Tamil Nadu in 1982 (Maan, Maan, Nomikou, Guimera, et al. 2012) and later from Tirunelveli district, Tamil Nadu in 2003 (Maan, Maan, Guimera, Pullinger, et al. 2012). The BTV-3 and BTV-9 were first isolated from outbreaks during 1973 from Himachal Pradesh and subsequently BTV-16 (Uppal and Vasudevan 1980). Kulkarni and Kulkarni (1984) isolated BTV-9 and BTV-18 using embryonated chicken eggs from Maharashtra in 1981. Jain et al. (1986) isolated BTV-1 in Rambouillet sheep from Central Sheep Breeding Farm (CSBF), Hisar, Haryana, India. The BTV-3 (from Tamil Nadu), -9 (from Uttar Pradesh), -16, -18 [isolated from sheep during severe BT outbreak in Rahuri, Maharashtra State in January 1988 and later it was confirmed as BTV-23 by sequencing of Seg-2 (Tembhurne et al. 2010; Maan, Maan, Guimera, Nomikou, Singh, et al. 2012)] and -23 (isolated in goats from Chakrata Block, Dehradun district, Uttarakhand in the first week of May 1995) serotypes were isolated from sheep in Maharashtra, Madhya Pradesh, Uttar Pradesh, Tamil Nadu, and Jammu and Kashmir; and however, year of isolation are not available (Mehrotra et al. 1989, 1995, 1996). Subsequently, BTV-3 was isolated in 2003 (Maan, Maan, Guimera, Nomikou, Morecroft, et al. 2012); however, species and place of isolation are not available.

Prasad et al. (1994) isolated the BTV-1 from sheep blood at Avikanagar in Rajasthan state in 1992 and partial sequencing of Seg-2/VP2 showed close similarity with Australian BTV-1 isolates (Dahiya et al. 2004; Maan, Maan, Nomikou, Prasad, et al. 2012). The BTV-1 was also isolated from Chennai, Tamil Nadu and Sirsa, Haryana, and sequencing of VP2 showed close similarity with Australian BTV-1 isolates (Dahiya et al. 2004); however, species and year of isolation are not available. Deshmukh and Gujar (1999) isolated BTV-1 from Maharashtra. Sreenivasulu et al. (1999) isolated the BTV-2 from outbreaks between 1991 and 1995 in native sheep of Andhra Pradesh and partial sequencing of Seg-2/VP2 showed close similarity (85.2%) with Taiwan isolate (Balumahendiran et al. 2009). The BTV-18 was isolated from sheep blood during BT outbreak in Bengaluru, Karnataka in 1998 and initially seotyped as BTV-18, later it was confirmed as BTV-23 by sequencing of Seg-2 (Tembhurne et al. 2010).

Bommineni et al. (2008) isolated and serotyped by SNT as BTV-2 (later RT-PCR analyses of BTV-2 were found to be BTV-9), -9 and -15 (later found to be BTV-10) serotypes from severe outbreaks during 2003 in native sheep in Andhra Pradesh. Subsequently, BTV-9 was isolated from sheep of Andhra Pradesh during 2007 (Rao, Reddy, and Hegde 2012) and 2008 (Rao, Reddy, Meena, et al. 2012). Two BTV-10 isolates are available from Andhra Pradesh, first, BTV-10 isolated from sheep blood in Nalgonda district of Andhra Pradesh during August 2003 (Gollapalli et al. 2012) and second, BTV-10 isolated in 2004 (Maan, Maan, Pullinger, et al. 2012). Joardar et al. (2009) isolated and serotyped by SNT as BTV-15 and BTV-21 from sheep in West Bengal (eastern India). Still, the presence of BTV-15 in India is based on SNT only, but the serotyping using Seg-2 sequencing has now been refuted (Rao et al. 2016). Biswas et al. (2010) isolated the BTV-1 from blood of goats suffering from peste des petits ruminants (PPR) in Mathura district, Uttar Pradesh during 2008 using BHK-21 cell culture and revealed close relatedness to Australian BTV-1 isolates in phylogenetic analysis of Seg-2. Minakshi et al. (2012) isolated BTV-16 from in-contact goats in Chennai, Tamil Nadu where clinical disease occurred in sheep; however, year of virus isolation are not available. Subsequently, Ranjan et al. (2016) isolated BTV-16 from blood of sheep affected with BTV infection from Karnataka in 2009. The sequence analysis of Seg-2/VP2 (7 bp) showed close similarity with several eastern BTV-16 viruses from India, Israel, Japan, Greece and Cyprus. The BTV-21 was first time isolated from sheep in Andhra Pradesh during 2005 and Seg-2 analysis showed close similarity with BTV-21 isolates from Japan (Susmitha et al. 2012). Chauhan et al. (2014) isolated BTV serotype-1 from aborted and stillbirth goat foetuses from Gujarat. Rao et al. (2015) isolated BTV-12 from blood of BTV affected nomadic sheep flocks of Adilabad district in Andhra Pradesh between 2010 and 2011. Subsequently, BTV-12 was isolated from sheep in Gurugram, Haryana (Dalal et al. 2017); however, year of isolation is not available.

Recently, BTV-24 was isolated from blood of sheep during BT outbreaks in 2010 in Medak district, Telangana and analysis of Seg-2 showed close similarity with western isolates of BTV-24. This indicated entry of exotic serotype into Australasian region (Krishnajyothi et al. 2016). More recently, BTV-5 was isolated for the first time in India from outbreaks in sheep during 2010–2011 in Karnataka and BTV-2 was also isolated from these outbreaks. Analysis of Seg-2 of BTV-5 showed close identity with BTV-5 from South Africa, indicating the virus was derived from western topotype, in contrast BTV-2 belongs to an eastern topotype (Hemadri et al. 2017). Several BTV serotypes were isolated from the same flock of different animals and/or even from the same animals, which indicated frequent circulation of several serotypes of BTV in one geographical region (Bommineni et al. 2008; Shafiq et al. 2013; Hemadri et al. 2017; Reddy et al. 2018). Reddy et al. (2018) isolated the BTV-4 from blood of sheep during BTV outbreaks between 2007 and 2013 in Andhra Pradesh and Telangana States and analysis of Seg-2 revealed close similarity with BTV-4 from China and belong to Australasian (eastern) topotype of BTV-4.

### 6.6. Complete/full genome sequences of BTVs from India

Full genome sequencing of BTV-2 (isolated from Tamil Nadu in 1982) showed nine genome segments belong to eastern topotype and Seg-5/NS1 belong to western topotype, indicating reassortment (Maan, Maan, Nomikou, Guimera, et al. 2012). Sequencing of BTV-2 (isolated from Tirunelveli district, Tamil Nadu in 2003) showed western topotype (Maan, Maan, Guimera, Pullinger, et al. 2012). Sequencing of BTV-2 (isolated in 1994) showed Seg-5 (belong to BTV-3 and BTV-23 isolates from South Africa) and Seg-9/VP6 (belong to BTV-10 isolate from the United States) showed close similarity with western topotypes. The Seg-2/VP2 (belong to eastern BTV-2 strains) and Seg-6/VP5 (Indian BTV-1 strains) showed close similarity with eastern topotype, indicating reassortment between its outer-capsid (VP2 and VP5) proteins (Maan et al. 2015). Sequencing of BTV-3 (isolated from 2003) showed nine genome segments belong to eastern topotype [Seg-2 and Seg-6 showed similarity with Japanese (eastern) isolates of BTV-3] and Seg-5 belong to western topotype, indicating incursion of western BTV strains. The reassortment between eastern and western field strains in India resulted in enhanced virulence of BTV outbreaks in indigenous sheep breeds in India (Maan, Maan, Guimera, Nomikou, Morecroft, et al. 2012).

Sequencing of BTV-9 isolated from sheep of Andhra Pradesh during 2008 showed Seg-2 and Seg-6 sequences belong to eastern topotype (close similarity with Mediterranean and European BTV-9 isolates) and Seg-5 belong to western topotype (similar to South African BTV-3) (Rao, Reddy, Meena, et al. 2012). Sequencing of BTV-9 isolated from sheep of Andhra Pradesh during 2007 showed Seg-2 and Seg-5 belong to eastern topotype (BTV-9 viruses from India and Europe), and Seg-6 showed similarity with BTV-5, which indicates classification of BTV-9 as a new serotype rather than as a topotype (Rao, Reddy, and Hegde 2012). Sequencing of BTV-10 isolated from Andhra Pradesh in 2004 (Maan, Maan, Pullinger, et al. 2012) and isolated from blood of sheep in Nalgonda district of Andhra Pradesh during August 2003 (Bommineni et al. 2008; Gollapalli et al. 2012) showed all genome segments belong to western topotype (BTV-10 vaccine strain from the United States), indicating the introduction of western vaccine strains into India. Sequencing of BTV-23 isolated from sheep in Rahuri, Maharashtra State in January 1988 showed the majority of the genome segments belong to eastern topotype. The Seg-5 showed close similarity with BTV-2, -3, and -9 from India (major western topotypes) and indicates reassortment (Maan, Maan, Guimera, Nomikou, Singh, et al. 2012).

Sequencing of Seg-1, -3, -4, -8, and -10 of BTV-12 (isolated from sheep of Adilabad district in Andhra Pradesh) belong to eastern topotype showed similarity with BTV isolates from India, Asia and Australia and Seg-9 showed similarity with BTV isolates from China and Taiwan (Southeast Asia). The Seg-5 belong to western topotype and Seg-7 belong to western topotype 1, which was not reported from India earlier, indicating entry of a new western topotype into India. The Seg-2 and Seg-6 are closely related with BTV12 isolates (Rao et al. 2015). Squencing of genome Seg-2 and Seg-6 of Indian BTV-16 and BTV-21 revealed Seg-6 of BTV-21 similarity with BTV-16 isolates. The BTV-21 reassorted by acquiring Seg-6 from BTV-16 isolate and significantly diverged from original BTV-21 strain (Shafiq et al. 2013). Full genome sequencing has been carried out for BTV-1 (eastern toptotype) isolated from sheep blood in Avikanagar, Rajasthan in 1992 (Maan, Maan, Nomikou, Prasad, et al. 2012); BTV-16 from goat in Chennai, Tamil Nadu (Minakshi et al. 2012); and BTV-12 isolated from sheep in Gurugram, Haryana, belonged to eastern topotype of BTV (Dalal et al. 2017).

### 6.7. Economic impact of BTV in India

The occurrence of BT in 2005 caused greatest direct annual economic losses to Indian sheep industry, accounting to approximately 231 million rupees (60.8%) among all diseases (Singh and Prasad 2009; Ranjan et al. 2015; Krishnajyothi et al. 2016; Rao et al. 2016). A study published in 2009, assessed the economic losses due to important diseases of sheep in India between 1991 and 2005, and BT was found to cause higher economic devastation than PPR, sheep and goat pox, FMD, and enterotoxemia (Singh and Prasad 2009). A total of 258 severe and repeated outbreaks of BT were recorded from Tamil Nadu during 1986 and 1995 (Sreenivasulu et al. 2004). BT caused a huge mortality of 3,00,000 deaths of small ruminants in Tamil Nadu during the monsoon season of 1997–98 (Ilango 2006). An outbreak of BT was reported in goats from Chakrata Block, Dehradun district, Uttarakhand in the first week of May 1995, where more than 60 goats died. The block had 80,000 sheep and goat population (Mehrotra et al. 1995). In India, between 1997 and 2005, endemic circulation of different BTV serotypes resulted in more than 2000 outbreaks in sheep, involving 0.4 million cases and around 64,000 deaths, making it the top viral cause of disease in sheep. In Andhra Pradesh, 880 outbreaks of BTV in sheep were reported in 2005 with 2,72,415 morbidity and 62,938 mortality with 23.1% of case fatality rate (Susmitha et al. 2012).

## 7. Global economic impact of BTV

BTV infection causes severe (direct) economic losses due to high morbidity, mortality, stillbirths, abortions, foetal abnormalities, less birth weight in young ones, reduced milk yield and fertility rate, weight loss, early culling, postponed gestations, absence of gestations, costs for restocking, meat and fleece losses. Indirect (expenditure and revenue losses) losses are due to trade restrictions imposed on ruminant animal movement, germplasm (semen and embryos) and other animal products, and expenditure for mass vaccination, diagnosis, surveillance, vector control and treatment of clinically pretentious animals (Tabachnick 2004; MacLachlan and Osburn 2006; Wilson and Mellor 2009; Caporale and Giovannini 2010; Sperlova and Zendulkova 2011; Rushton and Lyons 2015; Grewar 2016; Gethmann et al. 2020). It was estimated that BTV outbreaks caused economic losses of approximately US$3 billion in 1996 worldwide (Tabachnick 2004). The economic analysis for prevention of incursion of BTV-8 into Scotland revealed a total cost of approximately Euro (€) 141 million over the 5-year period (Gunn et al. 2008). The economic impact of BTV-8 outbreak in the US beef industry was US$95 billion in 2014. In the US livestock industries, losses due to trade restrictions and diagnosis for BTV status have been estimated as $144 million annually (Hoar et al. 2003).

The BTV-8 epidemics in Europe have caused great economic losses than any other previous single BTV serotype outbreaks (Wilson and Mellor 2009). The BTV-8 outbreaks in France and the Netherlands caused economic losses of US$1.4 billion and US$85 million, respectively during 2007. The losses are mainly due to the trade restrictions imposed during BTV outbreak time. The direct losses due to mortality, weight loss, reduced milk production, diagnosis, and treatment in the Netherlands and Scotland were estimated as £30 million per year (Gunn et al. 2008; Rushton and Lyons 2015). The overall economic losses (due to production losses and cost for diagnosis, treatment and control measures) in livestock industry ranged from €40.9 to 41.3 million in the Netherlands. The highest economic losses have occurred on sheep breeding farms (€12.6 million) and dairy export firms (€12.6 million), and dairy firms (€11.3 million). The production losses are 52.8 to 55.2% of the total net losses in the Netherlands. The production losses (due to mortality, early culling, stillbirths, abortions, postponed gestations, decreased milk production, weight loss, less birth weights, and decreased fertility of rams) in the Netherlands were estimated to be €32.4, 164–175, 12.2, 3.6, 2.6, and 6.6 million per year in 2006, 2007, 2008, 2009, 2011, and 2012, respectively (Wilson and Mellor 2009; Rushton and Lyons 2015).

The BTV-8 epidemic in the Netherlands caused direct losses of €28–32 million including €25 million in cattle and €3.5 million in sheep in 2006 and increased to €44 million in cattle and €5.5 million in sheep in 2007 (total loss €49.5 million). In contrast, the financial loss was €9 million in 2006 in Germany (Velthuis et al. 2010; Gethmann et al. 2020; Rushton and Lyons 2015). In the Netherlands, the cost for indoor-housing for sheep and goats was €18 million in 2006 (Velthuis et al. 2010). The total economic losses due to BTV-8 outbreak in Switzerland have been estimated as €12.2 million and €3.6 million for 2008 and 2009, respectively (Hasler et al. 2012). In contrast, the losses in cattle in Germany were €6.9 million and €21 million in 2008 and 2009, respectively; even though, cattle population is eight times higher in Germany than Switzerland (Gethmann et al. 2020). In Austria, the estimated total loss for BTV-8 surveillance and vaccination programmes were €22.8 million during 2005–2013 (Pinior, Lebl, et al. 2015). In the same period, cost of €96.6 million was reported for surveillance and vaccination in Germany due to six times more cattle population in Germany than Austria (Gethmann et al. 2020). The estimated cost for vector monitoring was €1.42 million in Austria and €94,000 in Switzerland during 2006–2010 (Pinior, Brugger, et al. 2015) and €1.2 million in Germany during 2007–2008 (Gethmann et al. 2020).

In the Netherlands, cattle density was more per farm (159.9/farm) than Germany (101.7 cattle/farm); however, cattle population is three times higher in Germany than the Netherlands (Gethmann et al. 2020). The BTV-8 epidemic in Germany caused a severe economic impact on the livestock industry (Gethmann et al. 2020). In the dairy sector, total direct loss from each BTV infected animal ranged from €119 to 136, depending on the milk price. Most of the losses are due to expenditure for restocking of elite animals (€99/animal), treatment (€26/animal) and production losses (€24 for reduced milk production and €18 for calf sales) in Germany. In the beef sector, average direct loss from each BTV infected animal was €27 due to prolonged fattening period. In sheep, average direct loss from each BTV infected animal was €74 in Germany. Most of the losses are due to reduced revenues for lamb sales (€59/infected ewe) and expenditure for veterinary treatment, especially after abortions (€10/animal). In Germany, economic loss due to morbidity was €11–71 million in 2006 and €13–308 million during 2007 in cattle (Gethmann et al. 2020).

The net total losses due to control and prevention of BTV-8 epidemic in Germany over the period of 13 years (2006–2018) ranged from €157 to 203 million (average €180.4 ± 6.0 million). This economic loss in Germany includes direct losses of average €48.3 million (27%) and indirect losses of €132.1 million (73%). This study did not include the losses caused by trade restrictions between August 2006 and September 2007 within Germany, because of 20 and 150 km restriction zones are frequently changed within short intervals (days or weeks). This resulted in an underestimation of losses in 2006 and 2007 (Gethmann et al. 2020).

Most of the indirect losses in Germany are due to the expenditure of €106.5 million (59% of the net total cost) for disease control programs including vaccination (€88.6 million including €74 million for cattle and €14 million for sheep), insecticide treatment (€18.0 million including €16.9 million for cattle and €1.1 million for sheep), export losses (€14.9 million, out of which €12.3 million solely of cattle), BTV surveillance and monitoring (€7.9 million including €1.2 million for vector monitoring in 2007 and 2008), and administration (€2.8 million). Most of the direct economic losses in Germany occurred in the cattle sector amounting €37.4 million (21% of net total cost) and in the sheep sector caused €10.9 million (6% of net total cost). The direct economic losses were €39.8 million (€29.7 million in cattle and €10.1 million in sheep) during 2007 in Germany. The animal compensation fund was paid to farmers in Germany during 2007 as €1500–1900 for each cattle (€17.3 million for 10,240 cattle) and €120–170 for each sheep (€4.2 million for 33,233 sheep), including rendering costs (Gethmann et al. 2020).

The annual economic losses in Germany were €66.8 million (37% of net total cost) and €59.1 million (32% of net total cost) in 2008 and 2007, respectively. After 2008, the economic losses were gradually decreased from €27.0 million in 2009 to €74,000 in 2014 due to the cost for voluntary vaccination borne by the farmers, so the number of vaccinations decreased. However, economic losses were started to increase again in 2015 and reached to €1.5 million in 2018 due to financial incentives were given to farmers to motivate and participate in voluntary vaccination for cattle, so that the vaccination costs started to increase. Most of the total annual economic losses in Germany are due to vaccination amounting €51.3 million (including €44.5 million for cattle, 25% of the total cost), €17.3 million for cattle (10%) and €7.9 million for cattle (5%) in 2008, 2009 and 2010, respectively. The losses due to monitoring and surveillance measures were €1.5 million in 2013–2015 and in 2015, costs increased due to the voluntary vaccination program (Gethmann et al. 2020).

In the Netherlands, during BTV-8 outbreak milk production was decreased to 5.4 kg/day for a period of 10.5 days and total milk production decrease was 51 to 56 kg per infected cow (Velthuis et al. 2010; Santman-Berends et al. 2011). Another study reported the economic losses due to reduced milk production in the Netherlands were estimated to range between €3 and 94 per cow (average €48/cow) (Van Schaik et al. 2008). The BTV-8 epidemic in France during 2007 caused a mean loss of 1.2%–3.4% (111–249 kg) of their total annual milk yield in cows (Nusinovici et al. 2013). Gethmann et al. (2020) reported the reduced milk production as of about 100 kg per infected cow in Germany.

The consequences of reproduction in BTV infected cows are increased incidence of return-to-service (RTS), short gestations, abortions, prolonged calving interval, and reduced fertility rate. The BTV-8 infected cows showed five times more likely to RTS (reduced fertility) within 56 days after the first insemination compared to non-infected cows in the Netherlands (Santman-Berends et al. 2010). The time between first and last insemination in BTV infected cows was 101.6 days. In addition, BTV-exposed farms showed 8%–21% of RTS in France than non-exposed farms (Nusinovici et al. 2012a). BTV infected ewes showed 15.7% increased incidence of abortions and reduced fertility rate from 59%–75% to 30% (number of pregnant ewes/number of ewes presented to the ram) in Belgium. In cows, calving interval was prolonged by about 80 days (Rushton and Lyons 2015). The BTV-8 infection in cows during early stage of gestation (before 90 days of pregnancy) resulted in increased (15%) incidence of RTS after calving and at 90 days of gestation resulted in no RTS. Further, BTV-8 infection in cows after third month of gestation resulted in 3.1%–6.7% increased incidence of RTS after calving. The BTV-8 infection in cows from third month of gestation resulted in increased (1.9%) incidence of short gestations (Nusinovici et al. 2012b).

## 8. Transmission

### 8.1. Culicoides midges

**Figure 2.**
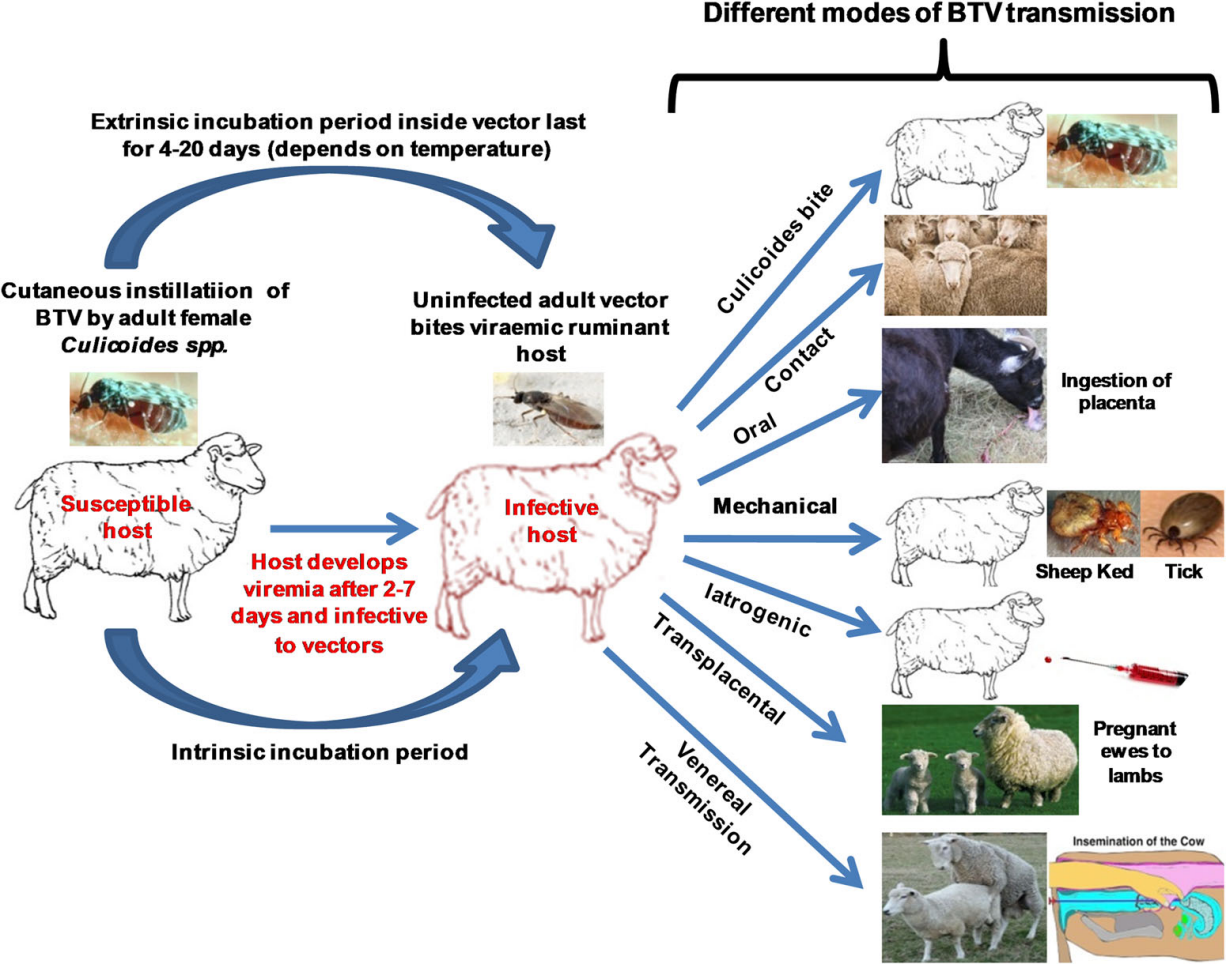
Various routes of transmission of bluetongue virus. Adult *Culicoides* spp. bites the infected ruminants, suck blood, virus replicate in vectors, and transmit to other susceptible animals by bite. The virus also transmitted to other susceptible animals by various routes like contact, oral, transplacental, venereal, mechanical, and iatrogenic transmission.

The ability of a vector to transmit a pathogen (vector competence) depends on several intrinsic and extrinsic factors. The factors are temperature, extrinsic incubation period (EIP), lifespan of vector, daily vector bite rate (frequency of feeding), daily vector survival rate, number of female vectors per host, host preference, host-to-vector transmission rate, and vector competence. The daily temperature fluctuations can lead to higher infection/transmission rates in insect vectors compared to constant temperature. BTV is transmitted by several species of haematophagus bittng midges belonging to the genus *Culicoides*, order Diptera and family Ceratopogonidae (Figure 2). The ambient temperature, environmental humidity in air, overall seasonal rainfall, wind speed, and swampiness during late summer and late autumn provide congenial conditions for distribution, survival and breeding of *Culicoides* vectors, which were directly coincided with transmission of BTV and outbreaks of BT (Mullens et al. 1995; Mellor 1990; Tabachnick 2004; Venter et al. 2004; Ilango 2006; MacLachlan and Mayo 2013; Benelli et al. 2017). Out of 1400 species of *Culicoides*, only about 30 are known to transmit BTV (Ander et al. 2012; Maheshwari 2012; Archana et al. 2016). Till date, 63 species of *Culicoides* were identified from different geographical regions of India (Maheshwari 2012). Seven *Culicoides* species namely *Culicoides actoni*, *C. brevitarsis*, *C. dumdum*, *C. fulvus*, *C. imicola* (*C. minutus*), *C. oxystoma*, and *C. peregrines* were the most predominant vectors for transmission of BTV in India (Jain et al. 1986; Ilango et al. 2006; Maheswari 2012; Archana 2016). *Culicoides* spp. responsible for transmission of BTV in different regions include *C. clavipalpis, C. anopheles* and *C. imicola* in West Bengal, *C. anopheles* and *C. actoni* in Uttar Pradesh*, C. simiklis* in Uttarakhand, and *C. orientalis* and *C. oxystoma* in Gujarat (Sreenivasulu et al. 2004).

*Culicoides* are tiny (1–3 mm) midges and obligatory blood feeder on mammals and birds (Ander et al. 2012). The midges are most frequent habitant of warm, humid and swampy areas, and animal shed, as they are rich in organic matter, fed by *Culicoides*. They are mostly active during night starting from just before the sunset till just after the sunrise in the morning. The BTV-infected *Culicoides* have the ability to fly over the short distances (up to 5 km) and/or they can be passively transported to long distances (up to 100 km) depending on the wind speed because of their small size, especially over the water bodies, which has been implicated as important source for emergence of new BTV outbreaks (Sellers et al. 1978, 1979).

Global warming is responsible for the longer survival of midges and increased the length of transmission of BTV (Tweedle and Mellor 2002). The adult midges usually live for about 20 days; however, depending on ambient conditions they can live for more than 90 days (Mellor 1990). Only female midges are infective and blood feeding. Usually, *Culicoides* vectors get infected by feeding on the BTV-infected hosts and remain persistently infected for their entire life span (Mellor 1990).

### 8.2. Transplacental transmission

Transplacental transmission (TPT) is recently identified as high epidemiological importance for BTV (Figure 2). TPT of BTV had been described with vaccination of pregnant animals with live-attenuated vaccine strains of BTV. The TPT by field or wild-type strains of BTV-8 in cattle, sheep and goats was first reported in Northern Europe in 2006 (De Clercq et al. 2008; Desmecht et al. 2008; Menzies et al. 2008; Backx et al. 2009; Worwa et al. 2009).

### 8.3. Venereal transmission

Various studies demonstrated the presence of BTV RNA from semen of viraemic bulls and rams mixed with red blood cells (RBCs) (Figure 2) (Bowen and Howard 1984; Wilson et al. 2008; Kirschvink et al. 2009). The extent of transmission to susceptible cows and ewes has not been demonstrated clearly (Bowen and Howard 1984). Transfer of pre-implanted embryos from infected donors resulted in absence of BTV transmission to healthy recipient cows (Venter et al. 2011). Hence, the transmission of BTV through infected semen and embryos are less important and this puzzling aspect needs to be investigated in detail (Hare et al. 1988; Al Ahmad et al. 2011; Venter et al. 2011).

### 8.4. Contact and oral transmission

Horizontal transmission has been reported in densely populated areas (Figure 2) and suspected commonly in white-tailed deer (Thomas and Trainer 1970) and cattle (Menzies et al. 2008). The contact transmission was experimentally confirmed with novel serotypes namely, BTV-25 (Chaignat et al. 2009), BTV-26 (Batten et al. 2013), BTV-27 (Schulz et al. 2016), and BTV-28 (Bumbarov et al. 2020). Oral transmission of BTV was reported in calves fed with infected colostrum (Menzies et al. 2008; Backx et al. 2009) and in carnivores due to ingestion of infected placenta and/or aborted foetuses (Alexander et al. 1994; Jauniaux et al. 2008; Maclachlan et al. 2009; Dubovi et al. 2013). Experimentally, oral transmission of BTV-8 was confirmed in IFN-Is receptor-deficient mice (Calvo-Pinilla et al. 2010). Oral transmission of BTV-2 was reported in control sheep that were housed near to the experimentally infected animal shed (Rasmussen et al. 2013).

### 8.5. Mechanical transmission

Some arthropods like sheep ked (*Melophagus ovinus*), mosquitoes and ticks can act as mechanical vectors for BTV (Figure 2); however, they are less important in disease transmission (Stott et al. 1985; Bouwknegt et al. 2010). BT is not a contagious disease, but BTV can be transmitted mechanically (iatrogenic) by contaminated surgical equipments and needles containing infected blood (Wilson and Mellor 2009; Sperlova and Zendulkova 2011).

## 9. Overwintering mechanism of BTV

Overwintering is the process by which persistence of BTV for an extended period, as no adult vector activity and replication of virus in the midges cease in the several winter months of the year in many temperate regions, during which the transmission of virus is almost completely interrupted. However, BT outbreaks often reoccur several months after the ‘silent’ period, which may be longer than the normal period of viraemia in ruminants or the lifespan of adult vectors (Takamatsu et al. 2003; Wilson et al. 2008; Mayo et al. 2016). In recent years, the global distribution of BTV has been tremendously altered by re-introduction of either infected adult midges or viraemic vertebrate hosts resulting in emerging and/or reemerging of BTV outbreaks into new unaffected temperate regions of the world by overwintering mechanism (Wilson et al. 2008; MacLachlan et al. 2009; Mayo et al. 2016). One of the assumed overwintering mechanisms was survival of BTV in adult *Culicoides* vectors. During winter season, usually adult *Culicoides* midges do not survive, but larvae of *Culicoides* has the ability to survive. However, no studies proved the survival and persistence of BTV in larvae of *Culicoides* (Mellor 1990; Takamatsu et al. 2003; van der Sluijs et al. 2016).

The TPT is important for viral epidemiology because TPT may result in the birth of immune-tolerant and persistently infected foetuses (De Clercq et al. 2008; Desmecht et al. 2008; Menzies et al. 2008; Backx et al. 2009; Worwa et al. 2009; Savini et al. 2014). A puzzling aspect of spread of BTV-8 in northern Europe is overwintering mechanism (Menzies et al. 2008). Animals that had recovered from BT between the autumn (end of midge season) and beginning of spring were exported from the Netherlands to Northern Ireland in January 2008. In February 2008, these cows calved and calves were positive for BTV and acted as carriers of BTV or source of infection to other animals by spreading the disease through midges, starting a new cycle of infection. Hence, it is believed that BTV overwinters in transplacentally infected calves. The viremia of *in utero* infected young-ones differs from prolonged viremia of adult ruminants. These findings suggested that special attention should be given to newborn animals during control and eradication program by screening of BTV in newborn animals.

Viremia in survived calves was up to 160 days after birth (Darpel et al. 2009) and in lambs 3 to 7 days after birth (Worwa et al. 2009). Viremia in lambs was reported to be highly variable and noticed from 10 days to maximum of 65–72 days born from ewes infected with BTV-2 during mid stage of gestation (Rasmussen et al. 2013; Savini et al. 2014). Except few reports of isolation of BTV from *in utero* infected calves (Menzies et al. 2008; De Clercq et al. 2008), isolation was not successfully demonstrated. The BTV-RNA load was found to be high in infected young-ones and demonstrated from blood and spleen of foetuses by qPCR (Chauhan et al. 2014; Saminathan et al. 2020); however, contribution of BTV positive young-ones to the epidemiology of BTV in vector free zones are not clarified. Still, there is neither evidence that vertically infected lambs can further transmit BTV by *Culicoides* vectors, nor that they can produce long-term antibodies.

Previous experiments described that BTV was isolated from resident immune cells of skin of sheep after completing the viraemic phase. The persistent BTV infection in ovine γδ T-lymphocytes played a vital role in overwintering mechanism. The CD8^+^ T lymphocytes, IL-2-dependent T-cells and null cells of bovine and ovine became persistently infected *in vitro* during the viraemia and virus replication (Whetter et al. 1989; Stott et al. 1990; Takamatsu et al. 2003). These persistently infected T-cells with BTV grow continuously in cultures without producing any cytopathic effects (CPEs). The conversion of persistent and silent infection of BTV in T-cells of animals to more clinical form of disease in other susceptible hosts would occur when midges feed on the persistently infected animals.

## 10. Species affected and reservoirs

In general, sheep, white-tailed deer, pronghorn antelope, bighorn sheep, American and European bison, mouflon, alpaca, llama, and yak have been reported as the most sensitive species to BTV infection (Robinson et al. 1967; Johnson et al. 2006; Darpel et al. 2007; Henrich et al. 2007; MacLachlan et al. 2008, 2009; Mauroy et al. 2008; Meyer et al. 2009; Falconi et al. 2011; Niedbalski 2015). Cattle, goat, camelid, and deer species (belong to subfamily Cervinae; Old World deer) showed subclinical BT, although these animals are susceptible to infection and BTV antibodies were also detected (MacLachlan et al. 2008, 2009; Batten et al. 2013; Coetzee et al. 2014). Viraemic period in cattle was longer than in sheep (MacLachlan 1994; Tweedle and Mellor 2002). Hence, cattle are considered as reservoir or maintenance or amplifying host for BTV due to prolonged and persistent viremia, which enhances the spread of BTV by many *Culicoides* spp. that preferentially, feeds cattle. Therefore, cattle play an important role in epidemiology of BTV (Barratt-Boyes and MacLachlan 1994). Prevalence of BTV antibodies and asymptomatic infection has been reported in domestic camels (*Camelus dromedarius*) in several countries including India, Middle East and North Africa (Malik et al. 2002). Experimentally infected camels with BTV-1 did not show clinical signs of BT for 75 days post infection (dpi). However, BTV infected camels developed lower levels of viraemia at 7 dpi, BTV-1 was isolated from blood, and produced neutralising antibodies against BTV-1 (Batten et al. 2011). These findings suggested that camels act as a reservoir for BTV and play an important role in the epidemiology and transmission of BTV over long distances and across borders (Batten et al. 2011). In the United States, abortions and stillbirth pups were reported in pregnant dogs vaccinated with contaminated vaccine or eaten carcass from infected animals (Alexander et al. 1994; Brown et al. 1996; Jauniaux et al. 2008; MacLachlan et al. 2009; Falconi et al. 2011; Dubovi et al. 2013).

### 10.1. BTV infection in wild animals

In wild animals, BT is endemic in most parts of Africa, North America, Europe and Indian subcontinent (Gerdes 2004; Falconi et al. 2011; Niedbalski 2015). Wild ruminants act as reservoir or maintenance host for BTV infection due to long-lasting viraemia, long-term carrier state and vector maintenance, and play an important role in its transmission (Niedbalski 2015). The existence of interconnected domestic and wildlife cycles could be responsible for the maintenance of BTV. BTV infection in susceptible wildlife species can result in variable outcome of infection from asymptomatic infection to fatal and sudden death (Falconi et al. 2011; Niedbalski 2015). Among wildlife, llama (*Lama glama*) (Meyer et al. 2009), alpaca (*Vicugna pacos*) (Ortega et al. 2010), white-tailed deer (*Odocoileus virginianus*) (Thomas and Trainer 1970; Howerth and Tyler 1988; Johnson et al. 2006), pronghorn antelope (*Antilocapra americana*), wild sheep such as mouflon (*Ovis aries musimon*) and bighorn sheep (*Ovis canadensis*), captive yak (*Bos grunniens grunniens*) (Mauroy et al. 2008), American bison (*Bison bison*), and European bison (*Bison bonasus*) are more susceptible to BTV infection and causes fatal clinical disease with high morbidity rate reported to be as high as 100% and case fatality rate up to 80%–90%, as that of domestic sheep (Robinson et al. 1967; Fernandez-Pacheco et al. 2008; Falconi et al. 2011; Niedbalski 2015). The clinical signs in BTV infected white-tailed deer are excessive salivation, oedematous swelling of head and neck, blood-stained nasal discharge, bloody diarrhea, widespread haemorrhages throughout the body, erosion and ulceration of hard palate, dental pad, gingiva, tongue, forestomachs and abomasum, coronitis, and laminitis with sloughing of hooves (Howerth and Tyler 1988; Thomas and Trainer 1970; Johnson et al. 2006). However, wild camelids showed prevalence of BTV antibodies and were found asymptomatically susceptible to BTV infection under natural conditions (Henrich et al. 2007; Meyer et al. 2009). The experimentally infected South American camelids (alpaca and llama) with BTV-8 showed mild clinical signs and indicates that camelids are resistant to BT and play negligible role in the epidemiology of BTV (Schulz et al. 2012).

Patton et al. (1994) conducted an experimental study in adult black-tailed deer (*Odocoileus hemionus columbianus*) and fawns by inoculating BTV-10 or -17. They reported BTV antibodies were detected in serum up to 692 dpi by SNT, AGID and c-ELISA assays. Under natural or experimental conditions, the clinical disease may occur in wapiti (*Cervus elaphus canadensis*), musk deer (*Moschus moschiferus*), ox (*Bos taurus primigenius*), axis deer (*Axis axis*), sika deer (*Cervus nippon*), fellow deer (*Dama dama*), Spanish ibex (*Capra pyrenaica*), and African buffalo (*Syncerus caffer*) (Falconi et al. 2011; Ruiz-Fons et al. 2014; Niedbalski 2015).

Natural infection of BT was also reported in African antelopes, elephants (Mushi et al. 1990) and wildebeest (*Connochaetes* spp.). However, in mountain gazelle (*Gazella gazella*), blesbock (*Damaliscus pygargus phillipsi*), red deer (*Cervus elaphus*), roe deer (*Capreolus capreolus*), and Eurasian elk (*Alces alces*) clinical signs are absent under natural or experimental conditions (Lopez-Olvera et al. 2010). BTV-8 RNA was detected in *Alpine chamois* from Switzerland. This animal was found at high altitudes and far from the domestic outbreaks, which suggested that the virus could spread into/through the Alps (Casaubon et al. 2013).

Wild and captive small and large carnivores (cheetah, lion, jackal, wild dog, large-spotted genet, and hyena) showed antibodies against BTV that had been fed with infected meat or by bite of *Culicoides* midges. The Eurasian lynx (*Lynx lynx*) from zoo showed seropositivity that had been eaten BTV infected stillborn or aborted ruminant fetuses (Alexander et al. 1994; Brown et al. 1996; Jauniaux et al. 2008; Falconi et al. 2011; Dubovi et al. 2013; Niedbalski 2015).

Though Indian subcontinent has more and very diverse population of wild ruminants, in India, no systematic survey has been conducted to know the exact prevalence of BTV infection in wildlife. Only one elephant serum sample has showed positive for BTV antibodies among tested samples (Mehrotra et al. 1989). Sambar deer (*Cervus unicolor*) showed seroprevalence of BTV from Tiger Reserve in Rajasthan state. Forty-three serum samples of chital deer/spotted deer (*Axis axis*) were negative for BTV-VP7 specific antibodies (10 serum samples were collected from Pench Tiger Reserve, Seoni district, Madhya Pradesh and 28 from State Forest Research Institute, Jabalpur, Madhya Pradesh during the year 2006–07 and 5 from Van Vihar National Park, Bhopal, Madhya Pradesh during the year 2011–12). During 2012–13, 26 serum samples of chital deer (out of 59 samples from Van Vihar National Park, Bhopal, Madhya Pradesh) were found positive for BTV antibodies (Sharma et al. 2012–13). Twenty-nine serum samples of Indian bison/gaur (*Bos gaurus*) were negative for BTV antibodies (14 serum samples during the year 2010–11 and 15 serum samples during the year 2011–12 from Kanha National Park, Madhya Pradesh). Six serum samples were negative for BTV antibodies [one from Indian gazelle/chinkara (*Gazella bennettii*) and 5 from nilgai/blue bull (*Boselaphus tragocamelus*) from Van Vihar National Park, Bhopal, Madhya Pradesh] during the year 2012–13 (Sharma et al. 2012-13). The overall seroprevalence of BTV was 19% (26/137) during the year 2006–13 (Sharma et al. 2012-13).

The BTV-VP7 specific antibodies were detected from 12 out of 20 (60%) Asiatic elephants (*Elephas maximus*) (10 samples from Dudhwa National Park, Lakhimpur Kheri, Uttar Pradesh and 10 from Agra Bear Rescue Facility (Wildlife SOS), Uttar Pradesh). Further, BTV antibodies also detected from 3 out of 5 (60%) blackbuck from Nandanvan Zoo, Raipur, Chhattisgarh state (Singh et al. 2015). Till date, outbreaks of BT with clinical manifestation have not been reported in wild animals from any part of the Indain subcontinent. However in India, there is high possibility of two-way transmission of BTV between domestic and wild ruminants in and around the Wildlife Reserves. Hence, extensive serological studies need to be conducted in wild ruminants to know the reservoir status of BTV infection in India. Still, there are too many gaps in the scientific knowledge on the relationships between BTV, BTV vectors and wild ruminants.

## 11. Pathogenesis of BTV

### 11.1. Replication of BTV in Culicoides

**Figure 3.**
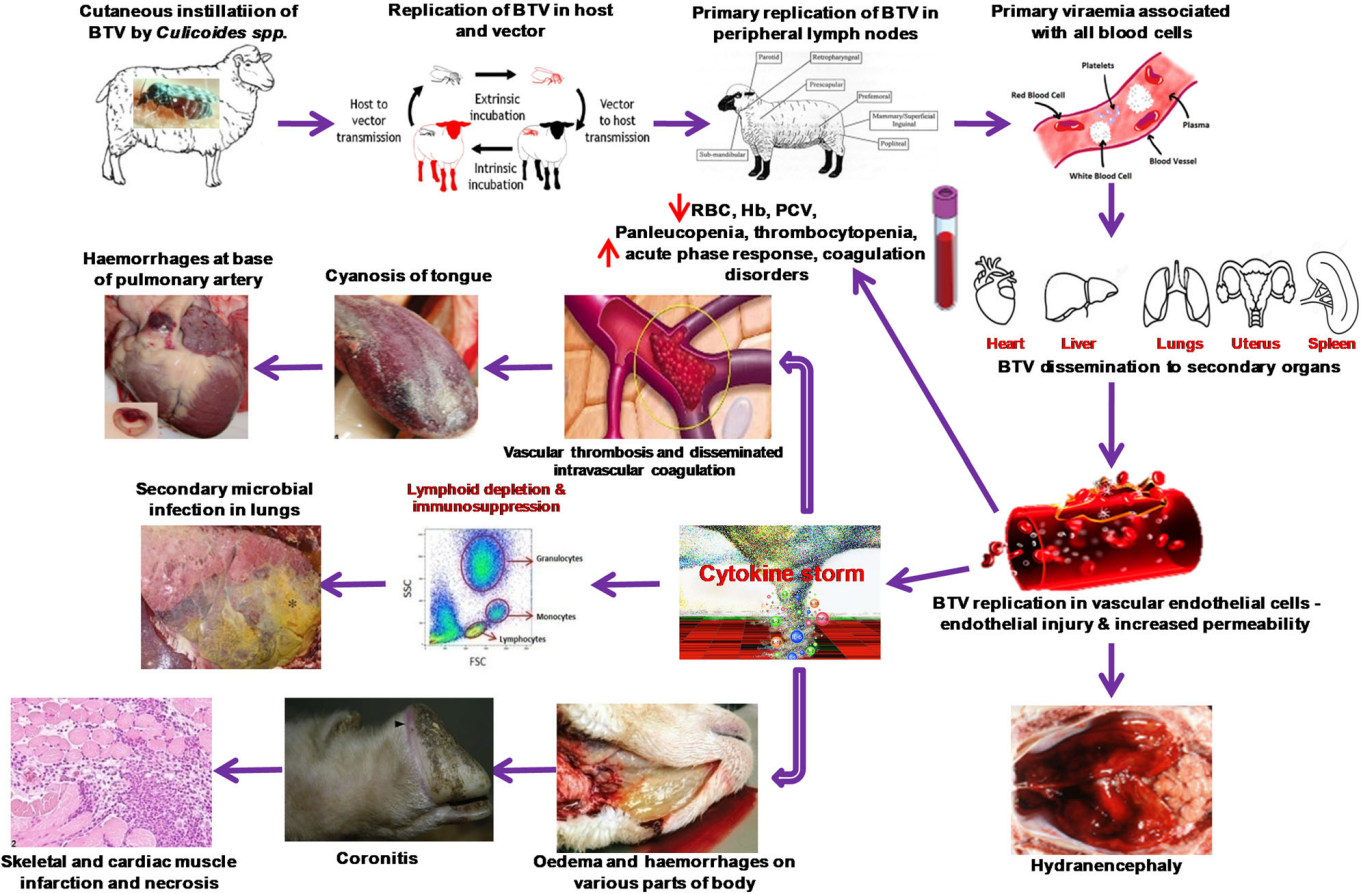
Pathogenesis of bluetongue virus in ruminants. After bite of an infected *Culicoides* spp. in ruminants, virus is transported by dendritic cells of skin to regional lymph nodes where primary replication occurs. Then virus reaches blood circulation, then disseminated to secondary organs where virus replicates in endothelium, and mononuclear phagocytes (dendritic cells, macrophages, etc). As a result of endothelial damage, excessive levels of various cytokines and vasoactive mediators are released (cytokine storm), which are responsible for pathogenesis of BT like increased vascular permeability, severe haemorrhages, oedema and effusions, thrombosis, infarction and disseminated intravascular coagulation. BTV infection in pregnant ruminants results in cerebral malformations in offspring.

*Culicoides* are biological vectors for BTV and *Culicoides* are infected by the bite of infected mammalian host through blood meal (Figure 3). Initially, BTV replicate in the midgut cells and escapes into the body cavity of vectors (haemocoel). Then, BTV infects and replicates in the salivary glands every 6–8 days, and now vector is ready to transmit BTV to other susceptible vertebrate host through bite (Wilson and Mellor 2009). These vectors remain infective for their entire lifespan. The extrinsic incubation period (EIP) is the time needed for the ingested virus from an infected vertebrate host to disseminate in midges (Figure 3) i.e. from midgut cells to salivary glands of midges (Wilson and Mellor 2009).

The duration of EIP is mainly determined by environmental temperature, which influences the activity of RNA-dependent RNA polymerase of VP1 protein of BTV. Foster and Jones (1979) reported that BTV replication was maximum after 12–14 days of incubation at 23 °C in *Culicoides variipennis* vector infected orally. At 20–25 °C, the duration of EIP is 10–16 days in BTV infected *C. variipennis* flies per os. BTV-11 infected sheep blood was fed to female *C. variipennis sonorensis* flies and kept at 15, 21, 27, and 32 °C. Virogenesis was significantly faster at higher temperatures and flies were infected on 4, 2 and 1 dpi when kept at 21, 27 and 32 °C, respectively. Peak levels of viral antigen was reported on 18–22, 7–13, and 5–7 dpi when flies kept at 21, 27 and 32 °C, respectively. Flies kept at 15 °C or below for 22 days showed no significant virus replication because at this temperatures the activity of *Culicoides* vectors reduces and activity of RNA polymerase of BTV also ceases, but BTV may persist in latent stage in infected vectors for entire lifespan, which was confirmed by keeping same flies after 27 °C and virus resume its replication, which was detected at 4–10 days. Hence, at below 15 °C, several weeks are required to complete the EIP. In summary, if *Culicoides* survives in an environmental temperature of 30 °C it resulted in completion of EIP in few days than *Culicoides* survives at 15 °C (Wittmann et al. 2002). These temperature-mediated mechanism favours the overwintering of BTV in *Culicoides* midges and role in BTV epizootiology (Mullens et al. 1995).

At higher temperature, less time is required for the digestion of blood meal in vectors, which increases the frequency of bite and feeding of blood. The most important effect of increased temperature is shortening of generation interval of BTV due to rapid replication of BTV resulted in increased chances of transmission of BTV into vertebrate host (Van Dijk and Huismans 1988; Wilson and Mellor 2009). However, abnormally high temperatures are detrimental to the vector survival (Wilson et al. 2008).

### 11.2. Replication and pathogenesis of BTV in vertebrate host

After cutaneous instillation by the bite of infected midges, BTV is transported from skin to the local lymph nodes (LNs) by dendritic cells (DCs) and initial virus replication occurs in LNs (Figure 3) (MacLachlan 2004; Hemati et al. 2009). Subsequently, BTV reaches the circulation and induces primary viraemia, which disseminates BTV to secondary organs such as LNs, spleen and lungs (Sanchez-Cordon et al. 2010). BTV replicates mainly in vascular endothelial cells (ECs), mononuclear phagocytic cells like macrophages, pDCs, conventional dendritic cells (cDCs), and lymphocytes of skin, LNs, lungs, spleen, and other tissues (Brewer and MacLachlan 1994; MacLachlan 2004; Hemati et al. 2009; Drew et al. 2010). During early viraemia, BTV is associated with all blood elements (Figure 3) (primarily with erythrocytes, and to lesser extent with the buffy coat fraction) and at later stages, it is exclusively associated with erythrocytes (MacLachlan 2004). Further, during early stage of infection, BTV was found in low titres in blood plasma (MacLachlan 1994).

The VP2 is a cell attachment protein and responsible for transmission of virus by RBCs. Usually, BTV is sequestered in the invaginations of RBC membranes (Brewer and MacLachlan 1994; MacLachlan 2004), which allows prolonged viraemia even in the presence of neutralizing antibodies but not persistent viraemia (Brewer and MacLachlan 1994). In sheep, viraemia last for 14 to 54 days and in goats for 19 to 54 days (Koumbati et al. 1999). Cattle with subclinical infections, viraemia develops as early as 4 dpi and viraemia may last as long as 60 and/or even 100 days, which makes cattle as an important host from the epidemiological point of view for BTV transmission (MacLachlan 1994; Darpel et al. 2007; MacLachlan et al. 2009).

The BTV has very strong affinity to endothelial cells and pericytes of capillaries, precapillary arterioles and venules. The interaction between ECs and host inflammatory mediators during BTV infection was studied *in vitro* using EC cultures (DeMaula et al. 2001). BTV replication causes apoptosis and necrosis of ECs of small blood vessels in target tissues characterized by cytoplasmic vesiculation, nuclear and cytoplasmic enlargement, pyknosis, and karyorrhexis by activating the p38MAP kinase (Barratt-Boyes and Maclachlan 1994; DeMaula et al. 2001; Mortola et al. 2004; Chiang et al. 2006; MacLachlan et al. 2009; Drew et al. 2010). The pathogenesis of BT attributed mainly to BTV induced damage/dysfunction of microvascular ECs and production of host inflammatory [type I IFNs, interferon-gamma (IFN-γ), tumor necrosis factor-alpha (TNF-α), interleukin-1 (IL-1), IL-2, IL-6, IL-8, cyclooxygenase-2, and increased plasma concentration of prostacyclin and thromboxane) and vasoactive (inducible nitric oxide synthase) mediators by virus infected ECs, platelets/thrombocytes, DCs, macrophages, and other cell types of bovine and ovine origin (Figure 3) (Stott et al. 1985; Howerth and Tyler 1988; DeMaula et al. 2001; Schwartz-Cornil et al. 2008; Hemati et al. 2009; Drew et al. 2010; Umeshappa, Singh, Ahmed, et al. 2011; Umeshappa et al. 2012; Channappanavar et al. 2012). These BTV-induced cytokine and chemokine mediators at optimal levels limit/controls the infection during early stages by activating innate immune responses and subsequently, stimulate the acquired immune responses. However, abnormally excessive levels of these mediators (also known as ‘cytokine storm’) are responsible for the pathogenesis of BT by stimulating abnormal inflammatory responses in ruminants characterized by increased vascular permeability (capillary leakage syndrome), severe haemorrhages (hemorrhagic fever), extensive oedema and effusions in various parts of body including lungs, vascular occlusion and tissue infarction resulting in consumptive coagulopathy/disseminated intravascular coagulation (DIC) leading to damage of cells and tissues of infected animals (Figure 3), which are characteristic of fulminant BTV infection of ruminants especially in white tailed deer (Stott et al. 1985; Howerth and Tyler 1988; DeMaula et al. 2001; Schwartz-Cornil et al. 2008; Hemati et al. 2009; MacLachlan et al. 2008, 2009; Drew et al. 2010; Umeshappa, Singh, Ahmed, et al. 2011; Umeshappa et al. 2012; Channappanavar et al. 2012). These changes are prominent in lungs, because lungs are rich in ECs.

Infected EC cultures produced IFN-γ at 20 and 40 hours post infection (hpi) (DeMaula et al. 2001). Coen et al. (1991) demonstrated the synergistic action of IFN-γ and TNF-α during early stages of infection and these molecules reduced the viral antigen expression on ECs. BTV infection causes the distruption of DNA synthesis and mitochondrial dysfunction in the target cells and enhances the major histocompatibility complex (MHC) class I antigen expression. The IFN-γ enhanced the expression of MHC class I and II antigens in the BTV infected ECs. The IL-1 cytokine alters the antigen expression on EC surfaces and inhibits the ECs growth.

Studies demonstrated that BTV infection disrupts the follicular DCs in sheep during early stages of infection thereby inhibiting B-cell division in germinal centers of lymph nodes resulting in delayed production of virus neutralizing antibodies and acute immunosuppression leading to predisposition to secondary microbial infections especially *Pasteurella pneumonia* (Umeshappa, Singh, Nanjundappa, et al. 2010). The delayed humoral immune response of host resulted in systemic dissemination of virus and clinical outcome of disease (Melzi et al. 2016).

Notably, the differences in susceptibility of ovines, bovines and caprines to BTV infection were reported. The following proposed mechanisms explain the differences in pathogenesis of BTV in cattle and sheep. In bovines, activation of ECs after BTV infection causes increased transcription of vasoactive and inflammatory mediators, and cell surface adhesion molecules. In contrast, in sheep, BTV infection causes rapid cytolysis of ECs with minimal activation of ECs and resultant mediators. Further, the ratio of thromboxane to prostacyclin is less in cattle when compared to sheep, which is responsible for the less susceptibility of ECs and resistance of cattle to BTV infection. In contrast, a significantly increased ratio of thromboxane to prostacyclin in sheep causes enhanced coagulation along with the occurrence of clinical manifestations and susceptibility of sheep to BTV-induced microvascular injury (DeMaula et al. 2001).

### 11.3. Role of apoptosis in pathogenesis of BTV

Apoptosis or programmed cell death is the mechanism of negative selection of cells that are deleterious and of no use to the body. The CD8 T lymphocytes (CTLs) are known to cause apoptosis in virus-infected cells (Stott et al. 1990; Janardhana et al. 1999). In addition, it is well documented that these cells, after their effector phase, themselves undergo rapid apoptosis (Umeshappa, Singh, Nanjundappa, et al. 2010; Saminathan et al. 2020). Apoptosis is responsible for the pathogenesis of BTV infection in ruminants (DeMaula et al. 2001; Mortola et al. 2004; Nagaleekar et al. 2007). BTV may trigger apoptosis in mammalian cells through various mechanisms (Umeshappa, Singh, Nanjundappa, et al. 2010). Mortola et al. (2004) reported apoptosis in mammalian cell lines induced by outer capsid proteins VP2 and VP5. Further, uncoating of BTV using VP2 and VP5 proteins (replication of BTV is not necessary) are responsible for the triggering of apoptosis through NF-κB pathway.

Studies have suggested that BTV induces apoptosis through intrinsic (caspase-9 activation) or extrinsic (caspase-8 activation) pathways (Mortola et al. 2004; Nagaleekar et al. 2007; Mortola and Larsen 2009; Umeshappa, Singh, Nanjundappa, et al. 2010). After BTV-10 and BTV-23 infections, caspase 8 was activated in the target cells and resulted in activation of extrinsic apoptotic pathway (Nagaleekar et al. 2007; Mortola and Larsen 2009). Then, sequential apoptotic pathways were activated including the activation of Bcl-2 family members, and its translocation into the mitochondria resulting in release of cytochrome c, Smac and Diablo. These findings suggest that activation of caspases, Bax, cytochrome c, Smac/DIABLO, and NF-κB are involved in the apoptotic mechanism during BTV infection (Mortola and Larsen 2009). However, later on, it was proved that both NF-κB and IRF responses induced by BTV infection at the early stage of infection are not responsible for the induction of apoptosis. Role of caspase-3 mediated apoptosis in BTV infected cells has been demonstrated in different cell lines *in vitro* (Mortola et al. 2004).

Interaction of BTV with lymphocytes and monocytes *in vitro* resulted in CPEs with apoptosis of these cells (Whetter et al. 1989). In addition, BTV induced inflammatory cytokine mediators also play a role in stimulation of apoptosis (DeMaula et al. 2001). Release of high levels of IFN-α from infected lymphocytes is responsible for the stimulation of apoptosis and subsequent depletion of CD8^+^ T-ymphocytes in early viral infection (Stott et al. 1990; Janardhana et al. 1999). Umeshappa, Singh, Nanjundappa, et al. (2010) reported the higher levels of apoptosis during BTV-23 infection in sheep, which was associated with decreased total leukocyte count, CD8^+^ T cells and Bcl-2 expression, and elevation of caspase-3 and IFN-α genes expression in MNCs. The IFN-α induced by BTV (Jameson et al. 1978; MacLachlan and Thompson 1985; Foster et al. 1991) mediates the apoptosis through various mechanisms by triggering release of pro-inflammatory cytokines (DeMaula et al. 2001). The IFN-α also stimulates the apoptosis by controlling nitric oxide (NO) production from infected ECs or activated macrophages (Umeshappa, Singh, Nanjundappa, et al. 2010). The IFN-α enhances the TRAIL expression on immune cells and also activates the TLR3-TRIF-RIP-FADD-caspase-8 dependent pathways resulting in clearance of virally infected cells through apoptosis. In IFNAR1-blocked mice, role of apoptosis in the pathogenesis of BTV during early and mid gestation was reported by Saminathan et al. (2020). Further, apoptosis in the foetuses and dams correlated with peak viraemia.

## 12. Immunopathology of BTV infection

### 12.1. Innate immune responses

The innate immune responses are the first line of defense against viruses by the production of IFN-α/β, other pro-inflammatory cytokines and chemokine mediators that control or limit the infection during the initial stages of infection. Innate immune responses are essential for the development of effective adaptive immune (humoral and cellular) responses (Vitour et al. 2014; Saminathan et al. 2019). Pattern recognition receptors (PRRs; located in the subcellular compartments, namely, endosomal and cytoplasmic) of the host recognizes the pathogen-associated molecular patterns (PAMPs) of pathogen (BTV dsRNA). The main sub-families of PRRs are retinoic acid-inducible gene 1 (RIG-1)-like family receptors (RLR) and toll-like receptors (TLRs) (Ortego et al. 2014; Vitour et al. 2014; Saminathan et al. 2019), resulted in activation of different signalling [Janus kinase (JAK)/signal transducer and activator of transcription (STAT)] pathways leading to the production of type I and type III IFNs, and other pro-inflammatory cytokines (Ortego et al. 2014; Vitour et al. 2014). The signalling complex enters the host cells resulting in stimulation of Interferon Stimulated Genes (ISGs) and subsequent expression of ISG-encoded proteins (Vitour et al. 2014), which contribute to the stimulation of an antiviral state in host cells by different mechanisms, such as blocking of viral translation, degradation of viral mRNAs, and arrest of cell growth. Further, RLR pathways control the sensing and antiviral responses against BTV in non-hematopoietic target cells (Chauveau et al. 2012).

### 12.2. Role of type I IFNs in BTV infection

The type I IFNs are IFN-α and IFN-β, which plays an essential role in anti-viral innate immune responses. Although BTV replicated substantially in cDCs and pDCs, only pDCs produces significant amount of IFN-α/β. BTV is a potent inducer of IFN-Is in many cell types from various tissues and host species, which was confirmed by many *in vivo* and *in vitro* models (Huismans 1969; Jameson et al. 1978; Fulton and Pearson 1982; MacLachlan et al. 1984; MacLachlan and Thompson 1985; Foster et al. 1991; Hemati et al. 2009; Vitour et al. 2014; Saminathan et al. 2018, 2019). Although, more than 40 years ago, induction of IFN-Is by BTV infection was reported, the mechanism of IFN-Is induction has remained unknown for several years. BTV was reported to induce IFNs from splenocytes, adult and foetal leukocytes of sheep, ECs of ovine and bovine origin (Coen et al. 1991; Chauveau et al. 2012), and cells of the kidney from pig, cat, monkey, hamster, and rabbit (Jameson and Grossberg 1981; Fulton and Pearson 1982). Interestingly, BTV has the ability to stimulate IFNs production more efficiently from various cell types of man especially from tumour-derived cell lines of human origin (Jameson and Grossberg 1978, 1981; Chauveau et al. 2012). The *in vitro* IFN induction from primary embryonic cells of murine origin infected with an attenuated American vaccine strain of BTV-10 was first demonstrated by Huismans (1969). Lyons et al. (1982) demonstrated that BTV-10 vaccine strain stimulated low IFN levels when compared with BTV-2, -4, and -6 field strains. Fulton and Pearson (1982) reported that BTV-10, -11, -13, and -17 strains have the ability to induce high levels of IFNs production. Recently, Chauveau et al. (2012) reported that European BTV-4 and -8 field strains have the ability to induce high levels of IFN-β in human and bovine cells.

The IFN levels were detectable as soon as 4 hpi and maximum levels reached between 8 and 12 hpi and then decreased to undetectable levels at 24 hpi (Vitour et al. 2014; Saminathan et al. 2018, 2019). The IFN-α/β was detected in the serum of sheep at 2 and 6 dpi inoculated with BTV-8. These findings suggested that pDCs and/or cDCs from blood and lymph can produce IFN-α/β after activation by BTV (Pascale et al. 2008). Interferons were detected from tissues and serum of experimentally infected bovine foetuses with BTV-10 at 125 days of gestation (MacLachlan et al. 1984). Calves experimentally infected with BTV-10 strain, produced peak circulating IFN levels at 1 and 3 dpi (MacLachlan and Thompson 1985). The BTV titres were higher in the blood when IFN levels were low in serum. This very early transient synthesis of IFNs suggested a dominant role of IFNs during initial antiviral responses rather than elimination of subsequent infection. In BTV infected sheep, a temporal relationship between IFN activity and viremia was reported and peak IFN levels decreased BTV titres by 90% (Foster et al. 1991). The IFN antiviral response detected in sheep was higher and lasted longer when compared to cattle.

### 12.3. Modulation or inhibition of type I IFNsby BTV

The NS3 and NS4 proteins of BTV play a vital role in counteracting the antiviral innate immune response of the host by modulating/inhibiting the IFN-Is synthesis signalling pathways (Belhouchet et al. 2011; Ratinier et al. 2011; Chauveau et al. 2013). The NS3 protein modulates the RIG-1 signalling and TBK1/IKKε activation pathways in epithelial cells (Chauveau et al. 2013). It modulates the host IFN responses by inhibiting the cellular transcription (Ratinier et al. 2011). Initially, BTV infection stimulated ISGs expression in A549 cells. However, activation of IFN-stimulated response element (ISRE) promoter and expression of ISGs were inhibited, when BTV-infected cells were treated with external type I IFNs. This modulation involves various mechanisms that are depending on the time of infection. BTV induced the redistribution of STAT1 in perinuclear areas at 8 hpi and interferes the activation of JAK/STAT signaling pathways after 12 hpi resulting in down-regulation of JAK1 and TYK2 proteins expression, which modulate/inhibit IFN-I responses (Doceul et al. 2014).

### 12.4. Humoral immune response

BTV is a highly cell-associated virus and the levels of neutralizing antibodies play an important role in protecting animals against BTV. The BTV infection in ruminants resulted in stimulation of antibodies against structural and non-structural proteins of BTV (MacLachlan et al. 1987). The outer capsid proteins VP2 and VP5 have the ability to induce neutralizing antibodies (Lobato et al. 1997). Significantly increased numbers of B-cells were found in superficial cervical LNs of calves infected with BTV-10 at 7 dpi (Barratt-Boyes et al. 1995). The BTV antibodies are usually detected on 7 to 28 dpi depending on the type of detection assay and route of animal inoculation. The BTV specific antibodies were detected in efferent lymph plasma collected from the challenged LN of calf on 9 dpi; however, BTV-specific antibodies were not detected in serum from the same animal upto 13 dpi (Barratt-Boyes et al. 1995). The BTV infected sheep and cattle showed evidence for seroconversion on 14 dpi (Ellis et al. 1990).

Prior to mid gestation, BTV infected ruminant foetuses develop immune competence (Osburn et al. 1981; MacLachlan et al. 1984). The IFN-Is and pDCs play a significant role in stimulating the B-cell responses and BTV-specific antibody production. Antibodies against VP7 core protein are serogroup specific and VP7 protein is conserved among the BTV strains and serotypes. Hence, detection of antibodies against BTV-VP7 by c-ELISA assay is commonly used for serological diagnosis of BTV infection in ruminants (Afshar 1994; Maclachlan and Mayo 2013). The BTV-specific neutralizing antibodies provide long-lasting protection to re-infection with homologous serotype of BTV (Huismans et al. 1987; Foster et al. 1991; Stewart et al. 2012). The basis of vaccination strategies to prevent BT is production of neutralizing antibodies (Savini et al. 2004, 2008).

The neutralizing epitopes of BTV are located in the specific interactive regions of VP2 protein. Sheep inoculated with BTV-VP2 antigen protein produces neutralizing antibodies that provides protection against challenge with virulent homologous BTV serotype (Huismans et al. 1987; Roy et al. 1994; Stewart et al. 2012). Epitheliochorial placenta in sheep did not allow the transfer of antibodies to the foetus. Hence, the presence of BTV antibodies in neonates born from infected dam during the period of vector inactivity strongly indicated the *in utero* infection by BTV (De Clercq et al. 2008). Saminathan et al. (2020) first time demonstrated the humoral immune responses in BTV-1 infected IFNAR1-blocked mice during early and mid gestation.

### 12.5. Cell-mediated immunity

The cell-mediated immunity (CMI) plays a critical role in controlling the spread of virus during early stages of BTV infection of ruminants by killing virus-infected cells (virus factories). However, CMI responses alone do not eliminate the viruses rapidly (MacLachlan 1994; MacLachlan et al. 2014). The CMI response is governed by host CD4^+^ and CD8^+^ T-lymphocytes. The development of immune response against BTV involves antigen presenting cells (APCs), such as cDCs and pDCs of skin (Hemati et al. 2009). Studies revealed that cDCs play a critical role in induction of BTV-specific CD4^+^ and CD8^+^ T-lymphocytes proliferation by synthesis of various cytokines (IL-12, IL-1β, IFN-γ and IL-6), which plays an important role in stimulating CMI responses and protection from BT (Hemati et al. 2009; Drew et al. 2010).

Studies in sheep showed that serotype-specific outer coat protein (VP2) and NS1 protein of BTV are major immunogens for CTLs and play a significant role in inducing CMI responses; whereas, VP5 and NS3 being minor immunogens (Janardhana et al. 1999). The NS1 and NS2 proteins of BTV specifically induce CTLs in sheep and mice that are cross reactive among different serotypes of BTV. Similarly, Calvo-Pinilla et al. (2012) reported the stimulation of CD8^+^ T-cells specific for NS1 provide heterotypic immunity in IFNAR^(−/−)^ mice immunized with recombinant BTV proteins. These findings suggest that polyvalent vaccine is a potentially feasible strategy to control and eradicate the BTV infection, because NS1 and NS2 proteins are relatively conserved among the different serotypes of BTV. Similarly, vaccines containing BTV core-like particles (CLPs) namely, VP3 and VP7 induced partial protection against either heterologous or homologous challenge in sheep. The vaccine containing VP2 protein provides the most complete protection against the respective serotype of BTV (Roy et al. 1994; Stewart et al. 2012).

Infection of cDCs from skin of sheep with BTV *in vitro* resulted in secretion of pro-inflammatory cytokines namely IL-1β, IL-6, IL-12, iNOS, and expression of co-stimulatory molecules, which favours the stimulation of CD4^+^ and CD8^+^ T-cells specific for BTV (Hemati et al. 2009; Umeshappa, Singh, Pandey, et al. 2010; Umeshappa, Singh, Ahmed, et al. 2011; Umeshappa et al. 2012; Channappanavar et al. 2012). BTV replication in the regional LNs resulted in stimulation of CMI responses characterized by increased number of B-cells and CD8^+^ T-cells in draining lymph nodes, and production of BTV-specific neutralizing antibodies (Barratt-Boyes et al. 1995).

BTV infection in ruminants resulted in significant alterations in population and dynamics of lymphocytes both systemically and locally, adjacent to the site of infection (Barratt-Boyes et al. 1995; Hemati et al. 2009; Umeshappa, Singh, Nanjundappa, et al. 2010; Umeshappa, Singh, Pandey, et al. 2010; Umeshappa, Singh, Ahmed, et al. 2011; Channappanavar et al. 2012; Saminathan et al. 2020). The BTV-17 infected sheep showed increased CD4/CD8 T-cells ratio (more than 3) in peripheral blood mononuclear cells (PBMCs) due to significantly decreased number of CD8^+^/cytotoxic/suppressor/MHC class I-restricted T-lymphocytes than CD4^+^/helper/MHC class II-restricted T-lymphocytes on 7 dpi with panlymphocytopenia. However, CD4/CD8 T-cells ratio was decreased (average 0.6) on 14 dpi due to increased number of CD8^+^ T-lymphocytes in sheep (Ellis et al. 1990). Similar temporal changes of T-lymphocyte subsets were reported in sheep infected with BTV-10 (Ellis et al. 1990). The T-lymphocyte subset alterations in BTV-17 infected cattle were minimal in PBMCs. Although CD4/CD8 ratio was normal (value of 1–2) with leukopenia and pan T lymphocytopenia on 7 dpi, the CD4/CD8 ratio was decreased (average 0.8) due to increased CD8^+^ T-lymphocytes on 14 dpi (Ellis et al. 1990). These findings suggested that differential immune responses were noticed in sheep and cattle following BTV infection, which might also be responsible for differential disease expression in ruminants (Ellis et al. 1990). Steadily increased proportion of CD8^+^ T-cells was found in superficial cervical and inguinal LNs of calves especially at 7 dpi and number of CD8^+^ T-cells were normal in lymph at 14 dpi. However, CD4^+^ T-cells in BTV infected group were normal as that of control animals (Barratt-Boyes et al. 1995). Umeshappa, Singh, Nanjundappa, et al. (2010) reported high CD4/CD8 ratio with panlymphocytopenia during early stages of infection on 3 dpi and decreased CD4/CD8 ratio on 15 dpi in experimentally infected sheep with BTV-23. Similarly, during experimental BTV-1 infection in sheep, CD4/CD8 ratio was high during early stages of infection on 4 dpi (∼3.4), and decreased at later stages of infection on 8 and 15 dpi (∼0.5 and ∼2.0, respectively). In spleen and PBMCs, CD4/CD8 ratio was high on 2, 4 and 8 dpi; however, CD4/CD8 ratio was decreased on 15 dpi and correlated with pro-inflammatory cytokines expression (IL-12, IFN-γ and TNF-α). These findings suggested the role of LNs during early immune responses against BTV infection (Umeshappa, Singh, Nanjundappa, et al. 2010; Channappanavar et al. 2012).

Saminathan et al. (2020) for the first time demonstrated the role of CMI responses in BTV-1 infected dam during early and mid gestation in IFNAR1-blocked mice. The CD8^+^ CTLs are activated for apparently transient period following BTV infection in mice and sheep, and their role in mediating the viral clearance is poorly studied (Jeggo, Wardley, and Brownlie 1984; Jeggo, Wardley, and Taylor 1984; Calvo-Pinilla et al. 2012). Administration of sensitized CTLs collected from BTV infected sheep provided partial protection in recipient sheep against challenge with either homologous or heterologous serotypes of BTV (Jeggo, Wardley, and Brownlie 1984; Jeggo, Wardley, and Taylor 1984; MacLachlan 1994).

Transfer of antibodies and T-cells in sheep can be induced via humoral and CMI responses, respectively, that are able to protect sheep against BTV infection (Jeggo, Wardley, and Brownlie 1984; Jeggo, Wardley, and Taylor 1984). Passive serum transfer containing specific neutralizing antibodies against BTV provides serotype-specific protection, indicating the role of neutralizing antibodies as *in vivo* antibody-mediated viral neutralization (Jeggo, Wardley, and Brownlie 1984; Jeggo, Wardley, and Taylor 1984). Several studies in mice and sheep models demonstrated the existence of cross-protective CTLs (Jeggo, Wardley, and Brownlie 1984; Jeggo, Wardley, and Taylor 1984) and cross-protective CD4^+^ and/or CD8^+^ T-cells to structural VP2 and non-structural (NS1 and NS2) proteins of BTV (MacLachlan et al. 2014).

## 13. Pathology of BTV infection

### 13.1. Clinical signs of BT

The clinical manifestations of BT in ruminants may vary from asymptomatic to lethal disease and acute or chronic (subclinical) conditions. The incubation period, clinical signs, morbidity and mortality rates of BT are highly variable based on the serotype/strain/topotype of BTV, route of infection, amount of virus, various host factors such as species, age, breed and immunological status of infected host, and environmental factors such as atmospheric temperature, solar irradiation exposure and vector population (Schwartz-Cornil et al. 2008; MacLachlan et al. 2009; Caporale et al. 2011; Umeshappa, Singh, Channappanavar, et al. 2011; Saminathan et al. 2018). Studies have reported that intradermal route of inoculation of BTV-23 is more potent in reproducing many aspects of natural infection such as prominent clinical pathology (pyrexia from 3 to 8 dpi, coronitis and lameness) and viral immune responses (including viremia, dissemination and localization to different organs) when compared to intravenous route (prolonged pyrexia was absent) of inoculation (Hemati et al. 2009; Umeshappa, Singh, Channappanavar, et al. 2011). The incubation period of BT in ruminants is highly variable, ranging from 4 to 20 days (average 4–10 days) (Tweedle and Mellor 2002). However, it is reported that during BTV-8 outbreaks in cattle and sheep in Europe both species had similar incubation period, which was consistent with normal BTV incubation period (Wilson and Mellor 2009).

**Figure 4.**
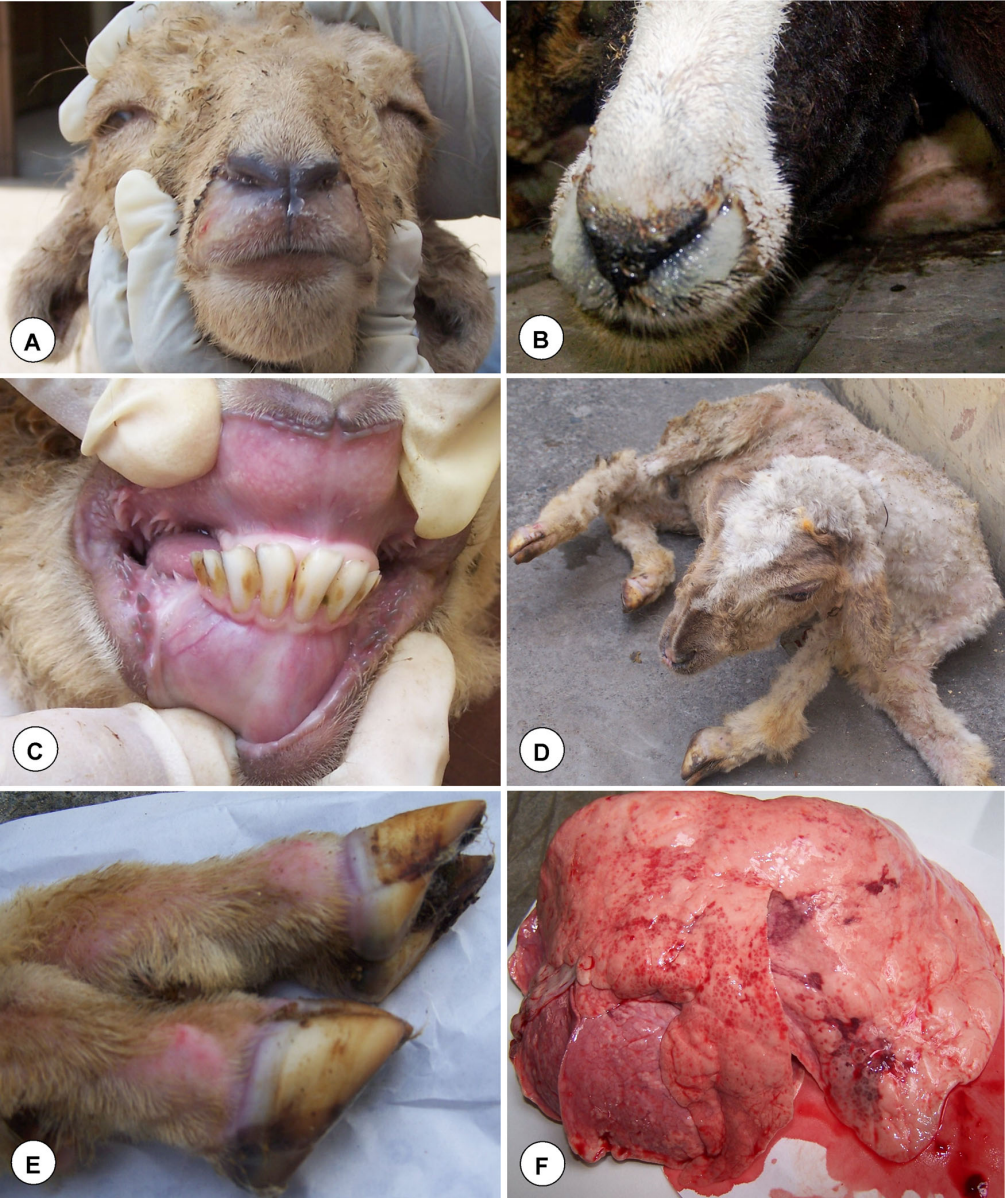
Clinical signs and gross lesions in bluetongue virus infected sheep. a. Hyperaemia and oedema of lips and nostrils with serous to mucoid nasal discharge. Lips are swollen with greyish brown necrotic deposition. b. Nasal area showed rhinitis and occluded by mucopurulent nasal discharge and excoriations. c. Oral mucosa showed congestion and oedema. d. BTV affected sheep showing weight loss, severe lethargy, dermatitis and break in the wool. Weakness, torticollis and reluctancy to move (knee-walking) as a result of necrosis of skeletal muscles and coronitis. e. Hyperaemia of coronary band (coronitis) in the feet. f. Lungs were heavy, oedematous, congested, haemorrhagic, and not collapsed. Areas of consolidation and emphysematous changes were noticed in apical and diaphragmatic lobes.

The BT affected sheep usually manifest fever, congestion and petechiae on conjunctiva, hyperaemia of lips and nostrils with serous (Figure 4(a)) to bloody nasal discharge, later on mucopurulent nasal discharge (Figure 4(b)), oedema of lips, tongue, face, ears and sub-maxillary region (‘monkey-face’ appearance), oral erosions (Figure 4(c)) and ulcers, weight loss, apathy, dermatitis, alopecia, and break in the wool (Figure 4(d)). Further, cyanosis of tongue (rare), excessive salivation (hyperptyalism), dysphagia, sloughing of the epithelium of tongue, gums and buccal commissures were observed (Brewer and MacLachlan 1994; Tweedle and Mellor 2002; Darpel et al. 2007; Kirschvink et al. 2009).

At the end of pyrexia, in later stages of disease (after 2 weeks of infection), affected sheep show hyperaemia of coronary band (coronitis) (Figure 4(e)), lameness due to lesions in interdigital space, and necrosis of striated/skeletal muscles manifested as paresis, weakness, torticollis (wry neck), arched back and reluctancy to move which can lead to knee-walking (Figure 4(d)) (Erasmus 1990; Worwa et al. 2009; Nusinovici et al. 2012a, 2012b; Susmitha et al. 2012). In severe cases, animals show respiratory distress (dyspnoea and tachypnea), bleeding from nostrils, profuse haemorrhagic diarrhea and vomiting that can cause aspiration pneumonia (Susmitha et al. 2012). Chronically affected sheep may succumb to other diseases such as bacterial pneumonia (Erasmus 1990; Pini 1976; Parsonson 1990; Backx et al. 2007; Darpel et al. 2007; Worwa et al. 2009; Umeshappa, Singh, Channappanavar, et al. 2011).

In cattle, clinical manifestations are less severe than sheep (Tweedle and Mellor 2002). However, clinical signs of fever, ocular and nasal discharges, conjunctivitis, ‘cracked’ appearance of muzzle, congestion and ulcers in oral mucosa, oedema and necrotic lesions in lips and tongue, coronitis, necrosis and ulceration in interdigital skin, reduced milk yield, superficial necrosis and exudation in mammary gland and teats, and severe neurological signs (especially in calves) were commonly reported in cattle during BTV-8 epidemic in Europe (Darpel et al. 2007; Williamson et al. 2008). In cattle, constant changing of position of the feet gave the nickname to BT as ‘dancing disease’.

Goats usually show mild clinical disease. However, BTV-8 epidemic in Europe caused severe clinical signs in goats like fever with high temperature, nasal discharge, oedema of lips and head, scabs on nose and lips, acute drop in milk production, erythema of the skin of udder, and subcutaneous haemorrhages (Dercksen et al. 2007).

### 13.2. Morbidity and mortality rates

Morbidity and mortality rates of BT in ruminants are highly variable, usually much lower in cattle and goats than in sheep. Morbidity in sheep ranges from less than 5% to 50%–75% or even higher up to 90%–100%. Although mortality in BT is often low, the mortality rate in susceptible breeds of sheep can be as high as 50%–70% and can reach up to 100%, as a result of secondary bacterial infections. The average mortality rate in sheep is usually up to 30%. In Africa, BTV is much more virulent and the mortality rate ranges from 2% to 30% (Gerdes 2004). In sheep flocks, BTV-8 caused average morbidity of 30% and mortality of 5%–8% and can reach over 70% in the Netherlands. In Germany, BTV caused case-fatality rates (CFR) of 37.5% and 41.5% during 2006 and 2007, respectively in sheep (Conraths et al. 2009). The BTV-8 outbreak in cattle in Europe caused average morbidity of 5% (in some herds caused higher morbidity) and mortality of less than 1% in affected herds (Wilson and Mellor 2009). The BTV-8 outbreak in Germany caused CFR of 6.4% and 13.1% in cattle during 2006 and 2007, respectively (Conraths et al. 2009). In goat flocks, CFR due to BTV-8 was up to 26% in Germany (Gethmann et al. 2020).

In India, first time occurrence of BTV in naive sheep flocks resulted in 50%–80% morbidity and 20%–50% mortality (average 2%–30%), which can reach up to 80%–100% in highly susceptible native sheep breeds (Sreenivasulu et al. 2004; Ilango 2006; Ranjan et al. 2015). The mortality rate of BT ranges from 2% to 50% in Karnataka, Maharashtra, and Andhra Pradesh (Gambles 1949; Rao et al. 2016). The BTV outbreaks in Andhra Pradesh caused a CFR of 21.9% and 2.4%–38.1% in 1983 and 1985, respectively (Sreenivasulu et al. 2004). The BTV outbreaks resulted in 9.3% of morbidity, 2.7% of mortality and 28.8% of CFR in rural sheep flocks of Andhra Pradesh. The BTV outbreaks resulted in 6.2% of morbidity, 0.5% of mortality and 7.6% of CFR in organised sheep flocks of Andhra Pradesh (Sreenivasulu et al. 2004). The BTV outbreaks in Maharashtra state caused 7.7% of morbidity, 1.1% of mortality and 11.8% CFR (Harbola et al. 1982). Later, severe form of BTV infection resulted in 32% of morbidity, 8% of mortality and 25% of CFR in rural areas of Maharashtra. In Tamil Nadu, BTV caused 3.3% to 22.8% of morbidity and 0% to 6.1% mortality (Sreenivasulu et al. 2004). Later, mortality rate was reported up to 17.2% in sheep in Erode district of Tamil Nadu during 2004–2008.

### 13.3. Gross pathological lesions

The lesions of BT are owing to BTV induced endothelial damage resulting in consumptive coagulopathy with secondary effects like widespread congestion, haemorrhages, oedema, vascular thrombi, infarction, and muscular degeneration and necrosis (Pini 1976; Mahrt and Osburn 1986). Gross lesions of BT include congestion, oedema, haemorrhages, erosion and ulceration of the mucosa of upper gastro-intestinal (oral cavity, esophagus and fore-stomachs) and upper respiratory tracts (Stott et al. 1985; Tweedle and Mellor 2002; Darpel et al. 2007; Mauroy et al. 2008; MacLachlan et al. 2009). The nasal areas of sheep and cattle showed rhinitis with ulceration and frequently occluded by greyish brown scab composed of desquamated epithelium, exudates and inspissated serum. In addition, nasal septum may be congested and excoriations are usually present on the muzzle (Erasmus 1990). Trachea may show oedema, congestion and extensive haemorrhages (Tweedle and Mellor 2002; Darpel et al. 2007; Mauroy et al. 2008; MacLachlan et al. 2009). The BTV affected animals frequently show hyperaemia, petechial to ecchymotic haemorrhages, oedema, erosion and ulcera with coats of grey necrotic tissues in lips, gingiva, dental pad, hard palate, sublingual, lateral surfaces, tip and anterior dorsum of tongue (occasionally cyanosis), and on interior surface of cheek opposite to molar teeth. The skin of infected animal often shows intense hyperaemia (erythema), particularly in the areas devoid of wool (ear and muzzle). The vascular networks in the skin are markedly congested and even the smallest blood vessels become very conspicuous. Subcutaneous tissues may show petechial haemorrhages (MacLachlan 2004).

The draining LNs of head, especially retropharyngeal, submandibular and pre-scapular LNs are more affected and show marked enlargement, oedema, congestion, and severe haemorrhages. Other LNs of head, neck and thorax are less severely affected. Thymus may show petechial haemorrhages. Spleen may show enlargement, congestion and subcapsular petechial haemorrhages. Tonsil, retropharyngeal and paranasal sinuses are often congested. Congestion and haemorrhages are also observed in thyroid gland. Animals that die after 14 dpi often show dramatic degeneration and necrosis of skeletal musculature resulting in severe wasting of body. Individual muscle fibres or entire muscles lose their pigmentation and intermuscular fasciae are infiltrated with clear fluid, resulting in gelatinous appearance of subcutaneous tissues. Trapezius muscles may be pale, slightly swollen, oedematous and loose (Tweedle and Mellor 2002; Darpel et al. 2007; Mauroy et al. 2008; MacLachlan et al. 2009).

Necrosis of cardiac muscles especially papillary muscles of left ventricle and petechial to ecchymotic haemorrhages at the base of the tunica media of pulmonary artery and subserosal haemorrhage at the base of the aorta are almost considered as pathognomonic lesions of BT. Multiple haemorrhages are often observed particularly in the pericardium, apex, endocardium and uni/bilateral papillary muscles of the heart (Spreull 1905; Stott et al. 1985; Parsonson 1990; DeMaula et al. 2001; Darpel et al. 2007; MacLachlan et al. 2009; Batten et al. 2013). Lungs were congested, haemorrhagic, oedematous, enlarged, heavy, and not collapsed with rib impressions (Figure 4(f)). Pleuritis, pulmonary oedema and effusions, and broncho-pneumonic changes along with areas of consolidation, emphysema and atelectasis involving apical and diaphragmatic lobes were noticed (Figure 4(f)). Trachea, bronchus and smaller bronchioles were filled with froth in BTV-8 infected sheep. Muscles of abdominal wall also showed oedema and the major body cavities like thorax, pericardium and abdomen were filled with fluid (Worwa et al. 2009).

There may be hyperaemia, erosion and multifocal hemorrhages on ruminal pillars and reticular folds (Worwa et al. 2009). Petechial haemorrhages are frequently found on the abomasal mucosa. Subserosal suffusion-type haemorrhages are consistently noticed in pyloric musculature, ileocaecal junction, and at the junction between the abomasum and duodenum, and on the reticulum. Enteritis may be present. Kidneys may show petechiae and mild congestion. Petechial haemorrhages in the gall bladder, mucosa of urinary bladder, urethra, vulva, and penis sheath may be prominent (Erasmus 1990).

### 13.4. Histopathological lesions

Histopathological lesions are hypertrophy of endothelial cells of capillaries, perivascular oedema and mononuclear cells (MNCs) infiltration around blood vessels (Stott et al. 1985; MacLachlan 2004). Vascular congestion with subsequent tissue infarction resulted in tissue hypoxia and epithelial cell desquamation. During acute BT infection, microvascular thrombosis, oedema, haemorrhages, degeneration and necrosis with large acidophilic intra-cytoplasmic masses and infiltration of neutrophils and MNCs (macrophages and lymphocytes) in target organs were reported (MacLachlan 2004; MacLachlan et al. 2008, 2009). Bekker et al. (1934) first described the microscopic lesions in the stratified squamous epithelium of oral mucosa and skin of sheep. The lesions include vacuolisation, degeneration, and necrosis of squamous epithelial cells of skin and buccal mucosal epithelium, mononuclear and polymorphonuclear cell infiltration in the underlying perivascular areas of dermis (Bekker et al. 1934; Channappanavar et al. 2012). Vascular congestion, MNCs infiltration in lamina propria and degeneration of muscle fibres of tongue (erosive and ulcerative glossitis) and trapezius muscles were reported (Parsonson 1990; MacLachlan 2004). Ulcerative necrosuppurative cheilitis and gingivitis were reported (Worwa et al. 2009). Skin showed degenerated and contracted hair follicles along with MNCs infiltration in dermal connective tissues, which are consistent with breakage and roughening of wool at later stages of disease. Erasmus (1990) reported intense hyperaemia of hoof corium mostly confined to tips of dermal papillae and associated with oedema and neutrophilic infiltration.

**Figure 5.**
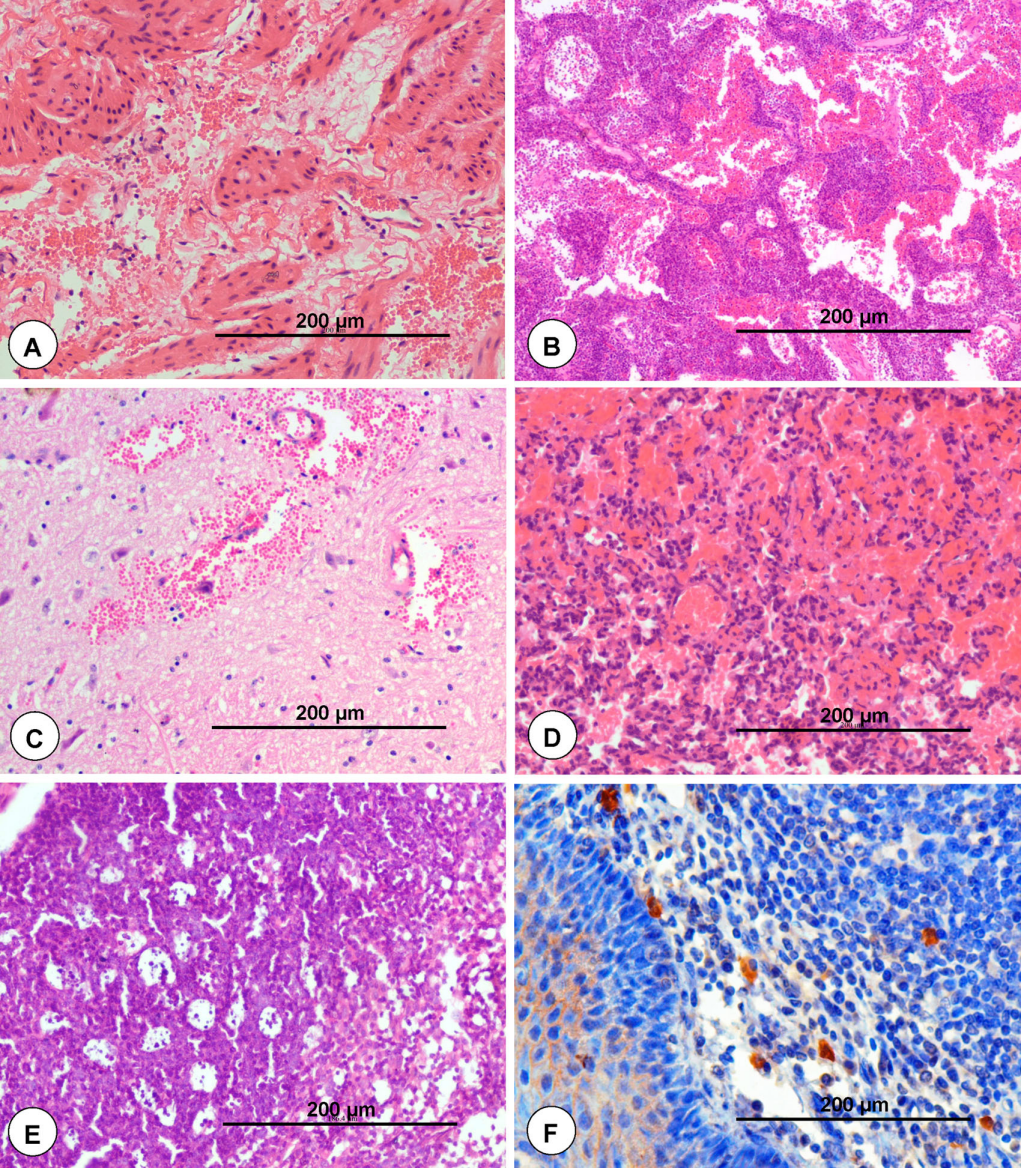
Histopathological lesions and immunohistochemical localization of bluetongue virus antigen in sheep and IFNAR1-blocked mice. a. Pulmonary artery of sheep showing severe hemorrhage, degeneration and hyalinization of tunica media. H&E, scale bar 200 µm. b. Lymph node of sheep showing severe hemorrhages and oedema in the medulla. H&E, scale bar 200 µm. c. Brain of sheep showd endothelial cell damage, severe hemorrhages and oedema in cerebrum. H&E, scale bar 200 µm. d. Lungs of IFNAR1-blocked mice showing severe hemorrhages and oedema due to endothelial damage. H&E, scale bar 200 µm. e. Lymph node of IFNAR1-blocked mice showing lymphoid depletion and starry-sky pattern due to apoptosis of lymphocytes. H&E, scale bar 200 µm. f. Positive immunolabelling of BTV antigen in the cytoplasm of mononuclear cells of skin of sheep. IP-DAB-MH, scale bar 200 µm.

In acute cases, affected skeletal and cardiac muscles invariably showed oedema, haemorrhages, hyaline degeneration and necrosis. In chronic cases, fibrosis and infiltration of mononuclear cells, especially macrophages and lymphocytes were noticed (Spreull 1905; Parsonson 1990; Tweedle and Mellor 2002; MacLachlan 2004; MacLachlan et al. 2009). Lesions in myocardium were more or less similar to the lesions of skeletal muscle. Acute focal to multifocal extensive hyperaemia and haemorrhages in tunica media of pulmonary artery (Figure 5(a)) and left papillary cardiac muscles are almost pathognomonic lesions (Worwa et al. 2009). Degeneration, hyalinization and fragmentation of elastic and smooth muscle fibres in the tunica media of pulmonary artery were noticed (Figure 5(a)). Lymphoid organs like lymph nodes, thymus and spleen showed apoptosis, severe lymphoid depletion and neutrophilic infiltration during early stages of infection and lymphocytolysis/reactive hyperplasia with increased DCs activity in medullary sinuses and proliferative changes in T- and B-cell areas during later stages of infection (Channappanavar et al. 2012; Saminathan et al. 2020). Lymph nodes also showed congested blood capillaries, hemorrhagic lymphadenitis (Figure 5(b)) and abundant apoptotic bodies/cells (Figure 5(e)).

Hyperaemia of oesophageal groove, haemorrhages with acute muscular degeneration of omasal folds, and multifocal erosive and necro-ulcerative rumenitis with thrombus formation were characteristic findings. Endothelial cell damage, focal haemorrhages and oedema were noticed in cerebrum (Figure 5(c)) and renal medulla (Worwa et al. 2009). BT affected lungs invariably showed sero-sanguineous oedematous fluid in alveolar lumen with or without neutrophilic infiltration, haemorrhages (leakage of RBCs into alveolar spaces) (Figure 5(d)), emphysematous, and atelectatic changes (Umeshappa et al. 2011). Diffuse interstitial pneumonia characterized by congested alveolar capillaries, thickening of inter-alveolar septa, and infiltration of MNCs and scarce neutrophils in alveolar lumen and septa.

## 14. Mouse models to study BTV infection

### 14.1. BTV infection in sucking mice

Newborn or suckling mice have been used as a model for studying the pathogenesis of BTV-induced cerebral malformations of ovine and bovine foetuses (Narayan and Johnson 1972; Waldvogel et al. 1987). Furthermore, newborn mice have also been used to assess the level of attenuation of live attenuated BTV vaccine virus by inoculating intracerebral (I/C) route. However, immunologically immature newborn mice are not suitable model to evaluate the BTV vaccines (Saminathan et al. 2019). Earlier studies have demonstrated that BTV-induced lesions in sheep and mice vary with the age of the host suggesting that the development of lesions appears to be influenced by stage of immunological maturity of the host. However, Narayan et al. (1972) studied the effects of cyclophosphamide and antithymocyte serum on BTV infection in mice of various ages. They reported that age dependency of lesions is determined by the availability of susceptible immature cells populations and not by the immunological immaturity of young mouse. Suppression of immune responses by these agents did not significantly increase the susceptibility of mice to BTV infection of central nervous system (CNS). However, pathologic sequelae were seen more in immunosuppressed animals and young animals than adults.

Narayan and Johnson (1972) reported that inoculation of foetal mice with BTV resulted in more extensive lesions in subventricular zone, hippocampal pyramidal cells and lesions were confined to the telencephalon. Postnatally, the subventricular zone is rapidly depleted. By 2 weeks of age, mitotic activity and migration is limited largely to a cluster of cells at the rostral extent of the lateral ventricles, which extend into olfactory bulbs forming the internal granular layer. This remnant of subventricular zone proved to be the sole susceptible cell population in 2-week-old mouse. In addition, previous studies demonstrated that BTV grew faster in 1-day-old suckling mice when inoculated I/C than at 2 weeks of age (Narayan and Johnson 1972; Waldvogel et al. 1987). Four-week-old mice with a very limited subventricular zone showed no evidence of infection. Likewise, adult mice are not susceptible to BTV infection and viremia was not observed in mice when inoculated either intravenously (I/V) or subcutaneously (S/C) (Calvo-Pinilla, Rodriguez-Calvo, Anguita, et al. 2009; Saminathan et al. 2018). Caporale et al. (2011) determined the virulence of high passage or low passage strains of BTV by I/C inoculation into 3-day-old mice. All these studies suggested that the possible constraint for the establishment of BTV infection in adult mice could be the innate immune responses against BTV generated by the host i.e. excessive production of type I IFNs, which establishes strong and brief antiviral state.

Brewer and Osburn (1998) demonstrated the neurotropism of BTV-11 strains namely, UC8 and UC2 in newborn mice following S/C inoculation. Both BTV strains were distributed selectively to brain and spleen as early as 3 hpi, but BTV was not detected in blood or other tissues up to 15 dpi. UC2 was detected in spleen and brain up to 9 hpi without CNS lesions. In contrast, UC8 was detected in brain throughout the experiment with severe necrotizing lesions in cerebrum and cerebellum at 11 and 13 dpi, and in spleen up to 24 hpi. The differences in neurovirulence between UC2 and UC8 strains of BTV-11 in mice were due to differences in replicative potential in the target cells (Waldvogel et al. 1987). Anjaneya et al. (2018) studied the comparative neuropathology of major Indian BTV serotypes (BTV-1, -2, -10, -16, and -23) by inoculating I/C in 3-day-old BALB/c mice. The BTV-1, -2, and -23 caused 65%–70% mortality with severe necrotizing encephalitis at 7–9 dpi. In contrast, BTV-10 and -16 caused 25%–30% mortality with mild neuropathological lesions at 9–11 dpi.

### 14.2. IFN-α/β receptor knockout mouse model

In wild-type mice, BTV is a potent IFN inducer (Jameson et al. 1978; Vitour et al. 2014; Saminathan et al. 2018, 2019). For many years, researchers tried to develop an adult mouse model to study the immune responses, pathogenesis, and vaccines against BTV. Testing of new vaccines in natural host like ruminants is a major obstacle due to stringent animal ethical issues, more animal purchase cost, difficult in purchasing seronegative animals, and requires specialized housing facilities (Coetzee et al. 2014). The genetically targeted knockout [IFNAR^(−/−)^] mice that are lacking the subunit of IFN-α/β receptors were unable to establish an antiviral state. The IFNAR^(−/−)^ mice have been used as a laboratory animal model to study the vaccine testing, determinants of virulence, and pathogenicity of many viruses including BTV (Calvo-Pinilla, Rodriguez-Calvo, Anguita, et al. 2009; Ortego et al. 2014; Saminathan et al. 2019). Infection of IFNAR^(−/−)^ mice with BTV strongly induces the production of IFN-α/β. However, the absence of these receptors does not allow the IFN-1 signal transduction and antiviral defense, resulted in increased susceptibility of IFNAR^(−/−)^ mice to viral infections (Calvo-Pinilla, Rodriguez-Calvo, Anguita, et al. 2009; Ortego et al. 2014).

The IFNAR^(−/−)^ mice was susceptible to many serotypes of BTV like BTV-1, -4, and -8, and differential virulence of serotypes were reported (Caporale et al. 2011). The clinical signs in BTV infected IFNAR^(−/−)^ mice are ocular discharges, apathy and disease progression led to mortality, and BTV was isolated from blood and various organs (Calvo-Pinilla, Rodriguez-Calvo, Sevilla, et al. 2009; Calvo-Pinilla et al. 2012; Ortego et al. 2014; Vitour et al. 2014). The BTV-8 has the capacity to infect IFNAR^(−/−)^ mice by oral route and this mouse model can be used to study the routes of transmission of BTV (Calvo-Pinilla et al. 2010). Advantage of IFNAR^(−/−)^ mouse model are easy availability of wide variety of antibodies and reagents that can be used in mice to study the many aspects of pathogenesis. The effect of pro-inflammatory cytokines were studied in BTV infected IFNAR^(−/−)^ mice, even though their innate immune responses are blocked (Ortego et al. 2014). The peak concentration of cytokines in serum was correlated with the gross pathological findings (Calvo-Pinilla, Rodriguez-Calvo, Anguita, et al. 2009; Calvo-Pinilla et al. 2010). The gross and histopathological lesions of BTV infected IFNAR^(−/−)^ mice were similar to that of BTV infected ruminants (Barratt-Boyes and MacLachlan 1994; MacLachlan et al. 2008, 2009; Schwartz-Cornil et al. 2008; Worwa et al. 2009). Even though, lack of IFN-1 signals may have adverse effect in the stimulation of acquired immune responses, IFNAR^(−/−)^ mice are also used for the study of efficacy of new BTV vaccines (Calvo-Pinilla, Rodriguez-Calvo, Sevilla, et al. 2009; Legisa et al. 2015; Mayo et al. 2017).

### 14.3. Immunocompetent wild-type mouse model after blockade of IFN-Is

An immunocompetent mouse model of BTV infection could help to understand the pathogenesis, host immune responses, and vaccine efficacy against BTV (Sheehan et al. 2006, 2015; Smith et al. 2017). Recently, a study was conducted to establish a wild-type adult mouse model for BTV infection after selective blockade of IFN-α/β receptors temporarily at the time of infection (Saminathan et al. 2019, 2020). The advantages of this mouse model are readily available, temporarily induced immune deficiency, more virus replication, and reversion of host to wild-type with normal immune responses. Adult mice with type 1 IFN gene blockade have been used as a model to study the BTV, Zika virus, vesicular stomatitis virus, and West Nile virus infections (Sheehan et al. 2006, 2015; Smith et al. 2017; Saminathan et al. 2019, 2020).

Recently, sequential pathology, apoptosis, virological and immunological responses were investigated in IFNAR1-blocked mice infected with BTV-1 during early and mid gestation (Saminathan et al. 2020). Administration of IFN-α/β receptor monoclonal antibody (MAb) (clone: MAR1-5A3) intraperitoneally, resulted in selective blockade of IFN-1 signal transduction and establishment of BTV infection in adult mouse. The MAR1-5A3 antibody blocks IFNAR1 signaling both *in vitro* and *in vivo* without depleting IFNAR1 bearing cells. A loading dose of 2.5 mg/mouse is required for *in* vivo blocking functional studies, to saturate all the binding sites. The half-life of 2.5 mg of MAR1-5A3 antibody is about 5.2 days and 250 μg is 1.5 days (Sheehan et al. 2006, 2015; Smith et al. 2017; Saminathan et al. 2019, 2020). The IFNAR1-blocked mice showed increased susceptibility to BTV infection, clinical signs and BTV RNA was detected in various tissues (Saminathan et al. 2020). The lower rate of mortality was observed at prolonged intervals when compared to early mortality and abnormally severe clinical disease in IFNAR^(−/−)^ mice (Calvo-Pinilla, Rodriguez-Calvo, Anguita, et al. 2009; Calvo-Pinilla, Rodriguez-Calvo, Sevilla, et al. 2009; Calvo-Pinilla et al. 2010; Ortego et al. 2014; Saminathan et al. 2019, 2020).

Saminathan et al. (2020) for the first time investigated TPT potential of wild-type or field strain of Indian BTV-1 in IFNAR1-blocked mice during early and mid gestation. Higher rate of TPT was reported during mid stage (71.43%) of gestation than early (57.14%) stage. The BTV-1 antigen was first time demonstrated in the cells of mesometrium, decidua of embryos, placenta, uterus, ovary, and brain of foetuses by immunohistochemistry and quantified by real-time qRT-PCR. This mouse model is highly suitable for further research into molecular mechanisms of TPT, overwintering, and vaccination strategies.

## 15. Laboratory diagnosis of BT

As of now, several diagnostic techniques are being developed for diagnosis of BT and practised widely throughout the world. BT was first diagnosed based on the clinical signs and appearance of cyanosed tongue as ‘epizootic catarrh’ and ‘bloutong’ (Spreull 1905). The field diagnosis of BT is usually made based on epizootiology, vector distribution, clinical signs, and pathological lesions. The clinical symptoms are pyrexia, congested conjunctiva, hyperaemia of lips and nostrils with serous to bloody nasal discharge and later on mucopurulent, swelling and oedema of muzzle, tongue, face and ears (‘monkey-face’ appearance), oral erosions and ulcers, excessive salivation (hyperptyalism), cyanosis of tongue (rare), coronitis, lameness, abortion, still birth, and congenital deformities/cerebral malformations (Pini 1976; Parsonson 1990; Darpel et al. 2007; Umeshappa, Singh, Channappanavar, et al. 2011). However, field diagnoses of sub-clinical and inapparent infections of BTV are difficult in cattle and goat. Therefore, different diagnostic techniques are developed and are broadly divided into two categories namely, BTV antigen (virus) detection and BTV antibody detection techniques (Anderson et al. 1989; Gould et al. 1989; Wechsler et al. 1990; Afshar 1994; Hamblin 2004; Rojas et al. 2019).

### 15.1. Detection of BTV antigen

#### 15.1.1. Virus isolation

Most commonly, blood is used for isolation of BTV, because BTV was found to be associated with RBCs. In early viraemia, BTV is associated with all blood elements and at later stages, it is exclusively associated with erythrocytes (MacLachlan 2004). The *in vitro* (Brewer and MacLachlan 1992) and *in vivo* (Nunamaker et al. 1992) studies revealed that BTV particles are either sequestered or engulfed in invaginations of the RBC membranes. Researchers have also reported that BTV can be transmitted through semen (Pearson et al. 1985) and that BTV can be isolated from raw or extended semen. Further, homogenised and properly stored tissue samples like lungs, spleen, lymph nodes, bone marrow, liver and brain can be used for BTV isolation (Jochim 1985; Parsonson 1990; Afshar 1994). Aborted foetal spleen is also used for the isolation of BTV. Bone marrow biopsy from adult sheep or liver and spleen biopsy from lambs can also be used as a source of BTV from live animals.

##### 15.1.1.1. Embryonated chicken eggs inoculation

Mason et al. (1940) first propagated the BTV in ECEs using yolk sac route of inoculation. Later, McKercher et al. (1957) propagated the BTV in ECEs using chorio-allantoic route. Now-a-days, BTV is isolated from field samples using 9 to 12 days old ECEs by inoculating I/V route at 33 °C. Intravenous route is widely used and 100–1000 times more sensitive than yolk sac route (Pearson et al. 1985; Wechsler and Luedke 1991; Clavijo et al. 2000; Dadhich 2004).

##### 15.1.1.2. Cell culture inoculation

BTV can grow in a wide range of cell lines derived from both insect and mammalian host. Most commonly used insect cell lines are KC cell line derived from *C. sonorensis* or *C. variipennis* midges and C6/36 cell line from *Aedes albopictus* mosquito used for isolation and propagation of BTV (Wechsler and McHolland 1988; McHolland and Mecham 2003). The CPEs are absent in cell lines of insect origin infected with BTV. Baby hamster kidney-21 (BHK-21), African green monkey (Vero) and mouse L cell lines are commonly used for growth and maintenance of BTV. Wechsler and Luedke (1991) had successfully propagated the BTV in calf pulmonary artery endothelial (CPAE) cell lines. They also reported that CPAE was more sensitive than 13 other cell lines. However, CPAE was less sensitive than ECEs, because CPAE was unable to detect BTV from the blood samples having lower concentrations of virus.

BTV induced characteristic CPEs are observed only in cell lines of mammalian origin (BHK-21 cell line) at 3 to 5 dpi and appears as foci of round, retractile and aggregation of floating dead cells. If CPEs do not appear, a second passage should be made in cell cultures. BTV can be confirmed from culture medium by sandwich ELISA and VNT (Wechsler et al. 1990; Wechsler and Luedke 1991; Sperlova and Zendulkova 2011).

#### 15.1.2. Animal inoculation

As an alternative, the primary host sheep is rarely used for the amplification and identification of BTV using washed RBCs or triturated infected tissues as inoculum. This method was first described by Spreull in 1902 as a sensitive and reliable method for BTV isolation (Spreull 1902). Disadvantages are large volume of inoculum required to be injected I/V (200–300 ml of blood, tissues or insect homogenate suspensions) and easily tolerated by sheep followed by development of anti-BTV antibodies (Howell et al. 1970; St George et al. 1978; Sundin and Mecham 1989; Wechsler and Luedke 1991). Sometimes, BTV can also be isolated by inoculating laboratory animals, such as mice or hamsters. Suckling mice of 2–3 days old are still used to study for pathogenesis of BTV isolates after I/C inoculation (Narayan and Johnson 1972; Narayan et al. 1972; Anjaneya et al. 2018).

#### 15.1.3. Sandwich ELISA

The antigen-capture ELISA (Ag-ELISA) or sandwich ELISA (s-ELISA) is very sensitive and most commonly used for detection of antigen. The s-ELISA detects the antigen between two layers of antibodies specific for different epitopes of the same antigen (capture and detection antibodies, referred as matched antibody pairs). Capture antibody is usually coated on the surface of the multi-well plates, which captures and immobilizes the antigen. The detection antibody binds to antigen and enzyme-linked or conjugated secondary antibody binds to detecting antibody. Finally, the substrate can facilitate the detection of antigen. The s-ELISA is highly efficient in diagnosis of BTV antigen directly from blood or clinical samples. The s-ELISA has number of advantages like economical, specific, rapid and an efficient tool for screening of large number of clinical samples within a short time and minimal technical expertise and equipment. This assay can be useful for conducting the epidemiological studies.

Either MAb or polyclonal antibody based s-ELISA are available for diagnosis of BTV antigen directly in blood samples, *Culicoides* midges or infected cell cultures. Polyclonal antibody based s-ELISA was less sensitive (Mecham 1993; Hawkes et al. 2000) than MAb based s-ELISA, which is highly sensitive and specific for detection of BTV in blood (Stanislawek et al. 1996). Chand et al. (2009) developed a polyclonal antibody-based s-ELISA for detection of BTV. The antiserum against BTV was used as capture antibody and antiserum against core protein (rVP7) of BTV was used as detection antibody. The assay was used to detect BTV-1, -2, -9, -15, -18, and -23 in cell culture supernatants. Gandhale et al. (2010) developed a MAb-based s-ELISA for the detection of group specific antigen (VP7) of BTV. Here, VP7 specific MAb was used as capture antibody and BTV polyclonal antiserum raised in rabbits was used as detection antibody. The assay was evaluated for diagnosis of group-specific antigen in BTV-1, -2, -15, -17, -18, and -23 serotypes in cell culture supernatants and could detect BTV antigen in clinical samples of blood (as early as 8 dpi), washed RBCs, buffy coat, and plasma. Ten Haaf et al. (2017) developed a MAb-based s-ELISA for direct detection of BTV in serum of infected animals. This s-ELISA has a limit of detection (LOD) of 10^4^ TCID_50_/ml.

#### 15.1.4. RNA-polyacrylamide gel electrophoresis

The RNA-polyacrylamide gel electrophoresis (RNA-PAGE) has been used as a diagnostic tool for detection of 10 genome segments of BTV, to identify the different serotypes and different genotypes of the same serotype of BTV (Prasad and Minakshi 1999). The RNA-PAGE has been used to compare the different electrophoretic migration patterns of various strains and serotypes of BTV (Sugiyama et al. 1982; Squire et al. 1983). Different serotypes of BTV have been reported to have different electropherotypes (Sugiyama et al. 1982). Prasad and Minakshi (1999) compared the sensitivity of RNA-PAGE and DIA for diagnosis of BTV antigen in cell cultures. The LOD of RNA-PAGE for detection of BTV-1 was 10^5^ TCID_50_/ml (Prasad and Minakshi 1999). The RNA-PAGE is a widely used technique for identification of BTV because of low cost, easy to use and high sensitivity (Prasad and Minakshi 1999; Minakshi et al. 2013). Minakshi et al. (2013) developed a novel staining method for the identification of BTV-RNA using RNA–PAGE. The ultrasensitive eriochrome black t–silver staining (EBT–SS) has been found to be 8 times more sensitive as compared to routine silver staining of BTV-RNA in non–denaturing RNA-PAGE. Using EBT–SS method all 10 bands of BTV genome were visualized up to 0.078 ng of BTV-RNA per lane when compared to routine silver staining that visualized up to 0.625 ng RNA per lane.

#### 15.1.5. Dot immunoperoxidase assay

The DIA was used for detection and identification of group specific antigen of BTV from infected cell culture fluids (Afshar et al. 1991; Afshar 1994). The DIA is an alternative to ELISA, but currently, DIA is not widely used (Afshar 1994; Rojas et al. 2019). Samples are absorbed as dots in the nitrocellulose membrane and immune reacted with MAbs directed against major group-specific antigen of BTV. Then incubated with HRP-conjugated IgG and positive color development was observed with DAB chromogen and H_2_O_2_ substrate. However, detection of BTV directly from infected ECEs tissue suspensions and sheep blood cells by DIA was unsuccessful (Afshar et al. 1991; Clavijo et al. 2000). The minimum LOD of BTV-1 by DIA was 10^5^ TCID_50_/ml (Prasad and Minakshi 1999). In addition, DIA was also used for diagnosis of BTV specific antibodies from field sera, because it is simple, rapid, coloured dots are stable and could be stored for long time for retrospective epidemiological studies (Prasad and Minakshi 1999). The BTV from blood samples or insect vectors was not detected by DIA at early passage levels. The DIA has certain advantages like economical, simple and easy to perform at remote laboratories, and does not require electrophoresis system when compared to RNA-PAGE.

#### 15.1.6. Virus neutralization test

The virus neutralization test (VNT) is most widely used in cell cultures for identification of BTV serotypes (serotyping of BTV isolate). Neutralization-based testing are the gold standard for serotyping of BTV isolates (OIE 2008). The basis of VNT is standard concentration of known serum (containing neutralizing antibodies against BTV) reacted *in vitro* and compared with serial dilutions of unknown/test sample containing BTV antigen resulting in neutralization of specific serotype of BTV (Afshar 1994; OIE 2008; Rojas et al. 2019). The disadvantages of this technique are requirement of reference sera to all known serotypes of BTV and handling of viruses simultaneously (OIE 2008). Infectivity of neutralized virus particles can be checked by subsequent inoculation of antigen–antibody mixture into susceptible *in vitro* systems namely, ECEs, cell lines, etc. Complete virus neutralization resulted in no virus replication, which is reflected by absence of CPEs or plaque inhibition on *in vitro* inoculated cell cultures (OIE 2008). The inhibition of virus growth and absence of pathological effects are indicative of specific BTV serotype (Haig et al. 1956).

#### 15.1.7. Electron microscopy

BTV particles can be diagnosed by transmission electron microscopy with a diameter of 80 to 90 nm (Gould et al. 1989; Eaton et al. 1990). This technique is a gold standard test, rapid and specific procedure; however, not widely used for diagnosis and not easily available in all the laboratories (Ranjan et al. 2015; Rojas et al. 2019). The immunoelectron microscopy requires specific MAbs to BTV and protein-A is labelled with gold to identify virus particles in sheep RBCs (Nunamaker et al. 1992), different serogroups of BTV, and to differentiate the BTV from epizootic haemorrhagic disease virus (EHDV) (Campbell et al. 1975; Eaton et al. 1990).

#### 15.1.8. Polymerase chain reaction

Since 1989, PCR is being used to detect BTV genome segments from the blood of infected animals (Gould et al. 1989; Prasad et al. 1999). The RT-PCR and real-time PCR are highly sensitive and rapid diagnostic technique for detection of BTV genome in various samples when compared to VI or neutralization assays (Dangler et al. 1990; Maan, Maan, Belaganahalli, et al. 2012; Saminathan et al. 2018, 2020). Conventional RT-PCR is advantageous over serological methods to identify the different serotypes of BTV within a single isolate containing ‘mixed’ serotypes (Prasad et al. 1999; Maan, Maan, Belaganahalli, et al. 2012). The RT-PCR is used for serotyping of BTV and can detect BTV-RNA in samples as late as 6 months after infection (Vanbinst et al. 2010: De Leeuw et al. 2015). The VP7 is a highly conserved protein and VP7 based primers amplified by PCR can be used to detect very low levels of BTV infection in tissues, blood, etc. The Seg-2/VP2 (primary determinant of BTV serotype) based primers are highly useful for identification of serotype specific BTV. Bandyopadhyay et al. (1998) developed a RT-PCR for detection of BTV in infected cell culture and clinical samples by amplifying 101 basepair (bp) nucleotide sequence of BTV genome Seg-6/VP5. Prasad et al. (1999) standardized the RT-PCR by amplifying 274 bp of Seg-5/NS1 gene for detection of BTV-RNA in cell cultures with sensitivity limit of 10 infectious particles of BTV.

Tiwari et al. (2000) standardized RT-PCR by amplifying VP7 gene of an Indian BTV-23. However, RT-PCR using VP7 primers works well with purified BTV-RNA from cell cultures, but insufficiently detect the BTV-RNA from clinical samples, and second step of nested-PCR is required to detect from clinical samples using VP7 internal primer/sequence (Tiwari et al. 2000). Nested PCR is about 100 times more sensitive than simple PCR, because two sets of primers are used; hence, it is useful for epidemiological studies of BTV. Nested PCR had detected a very small fraction of BTV genome (0.1 femtogram; 5 BTV particles) in cell culture and tissue samples using VP7 and NS1 based primers (Tiwari et al. 2000; Ayanur et al. 2016).

Duplex RT-PCR method was developed for simultaneous detection of BTV and host β-actin RNAs to minimize the false-negative results. Duplex RT-PCR is more specific and sensitive method than any other accepted BTV detection methods (Billinis et al. 2001). Maan et al. (2012) developed a rapid (within 24 h), sensitive and reliable RT-PCR-based typing assay for each BTV serotype by amplifying outer-capsid protein VP2 after nucleotide sequencing and phylogenetic analyses of 26 BTV serotypes (9 from Europe and 15 from the United States). The serotype-specific primers are used for identification and differentiation of 26 BTV serotypes and showed no cross-amplification with remaining 25 serotypes. The RT-PCR has limited use for detection of BTV from raw or extended semen samples (Akita et al. 1993).

Various real-time quantitative PCR (qRT-PCR) assays were developed for reliable and rapid detection of BTV genome by targeting Seg-1/VP1 (Shaw et al. 2007; Toussaint et al. 2007), Seg-5/NS1 (Toussaint et al. 2007), Seg-2/VP2 and Seg-10/NS3 (van Rijn et al. 2012). However, Seg-5 has been recognized as most conserved genome of BTV, hence recommended to identify all available serotypes of BTV. The qRT-PCR showed higher sensitivity, detect and quantify very low levels of BTV-RNA from target tissues of viraemic animals, cell cultures and semen than conventional RT-PCR (Umeshappa et al. 2011; Saminathan et al. 2020). The qRT-PCR act as a useful tool for screening of semen from BTV affected rams and bulls (MacLachlan 2004; Kirschvink et al. 2009). TaqMan fluorescence-probe based qRT-PCR is a highly sensitive diagnostic tool for first-line diagnosis of BTV (MacLachlan 2004; Mertens et al. 2007; Hoffmann et al. 2009; Maan, Maan, Belaganahalli, et al. 2012).

#### 15.1.9. Sequencing techniques

BTV genome sequencing enables classification into serogroup, serotype and even topotype (geographical origin) of BTV (Maan et al. 2010; Maan, Maan, Nomikou, Guimera, et al. 2012; Maan, Maan, Pullinger, et al. 2012). If full genome sequences of different BTV isolates are available, serotype-specific primers are readily designed for rapid diagnosis and identification of new strains/serotypes of BTV. However, disadvantage of sequencing technique is that it is expensive. If prices of sequencing become reasonable, then sequencing can be used as a routine diagnostic test.

#### 15.1.10. Blotting and hybridization

The Northern (detection of RNA molecules) and Southern (detection of specific DNA molecules) blot techniques are used to detect the BTV genome through hybridization with complementary DNA (cDNA) or RNA probes from different segments of BTV genome and serotypes. The advantages of nucleic acid hybridization techniques are high sensitivity, specificity and less laborious than virus isolation. Hybridization techniques are difficult to perform as a routine diagnostic test in the laboratories because of laborious separation of nucleic acids by gel electrophoresis and difficulty in optimizing the test protocols (Rojas et al. 2019). *In situ* hybridization (ISH) technique is used for the exact localization of specific portion of viral nucleic acids within a histological section. The viral nucleic acids in tissues are detected by using complementary strand of nucleic acid to which a reporter molecule is attached. Visualization of the reporter molecule allows the localization of viral nucleic acids from the tissues.

The ISH technique has been used to detect BTV nucleic acids in tissues and cell cultures blotted onto solid membranes (Dangler et al. 1988; Schoepp et al. 1991; Brown et al. 1996). The sensitivity of ISH for detection of BTV-17 was equivalent to that of virus isolation and antigen detection techniques namely, indirect fluorescent antibody or enzyme immunocytoassay. The sensitivity of ISH for detection of BTV-2, -10, -11, and -13 were not sensitive as that of VI and antigen detection techniques. This indicates detection limit of ISH was depending on the BTV serotypes in cell cultures (Schoepp et al. 1991). However, ISH failed to detect BTV in blood mononuclear cells, although blood was collected during peak viremia (Dangler et al. 1990). The BTV nucleic acid was detected predominantly in endothelial cells of lungs, uterus, MNCs of periarteriolar lymphoid sheath of spleen, kidneys, and placenta of dog by ISH using digoxigenin-labeled probe of NS-1 gene of BTV-17.

#### 15.1.11. Haemagglutination and hemagglutination inhibition tests

The RBCs from sheep, cattle, pig, guinea pig, mouse, rabbit, chicken and goose can agglutinate BTV with variable intensity (van der Walt 1980). Studies also reported that some BTV serotypes can agglutinate limited range of RBCs, while some other serotypes can agglutinate a wide range. The haemagglutination (HA) property of BTV was independent of variations in the pH, temperature, buffer system and host species of erythrocytes. For this non-uniformity and lowered HA activity of BTV, HA test is not been widely used (Cowley and Gorman 1987). The HA property was inhibited by serotype specific serum and hemagglutination inhibition (HI) test was developed to identify various BTV serotypes and to determine BTV antibodies. The HI test was found to be serotype specific (van der Walt 1980).

#### 15.1.12. Complement fixation test

The CFT is less commonly used for identification of BTV or to detect an antibody titer in infected animals. From history point of view, it is important to note that detection of the first Australian BTV isolate was done by using CFT (St George et al. 1978). Using CFT, tissues or fluids from infected ECEs can be detected by reacting with hyperimmune sera raised in laboratory animals and infected tissue culture fluid act as antigen (McKercher et al. 1953; Shone et al. 1956). Carrier and Boulanger (1975) developed a modified direct CFT to detect BTV antibodies from cattle and sheep serum. Serotyping of BTV isolates is restricted in this method. The CFT is abandoned and used only in few laboratories due to non-availability of anti-complementary serum and complexity of the procedure (Afshar 1994).

#### 15.1.13. Immunoperoxidase method

The immunoperoxidase or immunohistochemistry (IHC) technique enables detection of BTV antigen from infected formalin fixed or frozen tissue sections at the site of tissue damage (Figure 5(f)). In addition, it allows identification and distribution of BTV antigen in different tissues and specific cell types (Mahrt and Osburn 1986; Anderson et al. 1989; Sanchez-Cordon et al. 2010). Primary antibodies against BTV rose in rabbit or sheep are allowed to react with peroxidase conjugated anti-species secondary antibody resulting in formation of characteristic positive colour development (Anderson et al. 1989; Wechsler et al. 1990; Saminathan et al. 2018, 2020). Sanchez-Cordon et al. (2010) demonstrated the VP7 of BTV in arteriolar and capillary endothelial cells, macrophages and lymphocytes of spleen, lymph nodes and lungs using IHC in Bouin’s and formalin-fixed tissues from sheep and goat naturally infected with BTV.

#### 15.1.14. Immunofluorescence assay

Several researchers have developed MAbs, mainly against VP7 protein that can be used for the detection of BTV serotypes. The immunofluorescence assay is a conventional technique, MAbs or antisera (primary antibodies) are allowed to bind with specific secondary antibodies tagged with different fluorochromes such as fluorescein isothiocyanate (FITC) (Pini et al 1966; Afshar 1994; Umeshappa, Singh, Channappanavar, et al. 2011; Rojas et al. 2019).

### 15.2. Detection of antibodies against BTV

#### 15.2.1. Indirect ELISA

The i-ELISA is a simple and rapid technique for detecting and quantifying the antibodies in samples (Kramps et al. 2008; Barros et al. 2009; Mars et al. 2010; Chand et al. 2019; Rojas et al. 2019). The i-ELISA has two-step process, which requires primary and labeled secondary antibodies. The basis of i-ELISA includes r-VP7 antigen of BTV was coated onto 96-well microtiter plates and test serum (containing BTV antibodies), peroxidase conjugated secondary antibody, and substrate (OPD) were added. The serogroup-specific i-ELISA using rVP7 protein as antigen and peroxidase-conjugated secondary antibody for detection of group-specific BTV antibodies in serum samples from different species have been developed (Kramps et al. 2008; Chand et al. 2019). The diagnostic sensitivity (DSn) and diagnostic specificity (DSp) were 96.1% and 98.5%, respectively (Chand et al. 2019). The milk ELISA (m-ELISA) in the form of i-ELISA was developed for the detection of BTV specific antibodies in bovine milk samples (Kramps et al. 2008; Mars et al. 2010). The DSp of i-ELISA was 96.5% and DSn was 98.9% for milk samples (Kramps et al. 2008). The i-ELISA was found to be reliable and very useful diagnostic tool for surveillance purposes (OIE 2008). The i-ELISA can also be used as a DIVA tool for detection of BTV-NS3 antibodies (Barros et al. 2009). Recombinant NS3 antigen based i-ELISA is used for the differentiation of serum from BTV infected and vaccinated animals. Higher levels of antibodies against NS3 were detected in the infected animals than vaccinated animals. The i-ELISA might be useful tool for DIVA strategy than c-ELISA that does not have DIVA (Rojas et al. 2019). The disadvantages of i-ELISA are that species-specific secondary antibody conjugates are needed, which is a practical limitation for routine sero-diagnosis of a multi-species disease like BT (Chand et al. 2017).

#### 15.2.2. Competitive ELISA

The c-ELISA is also known as inhibition ELISA or blocking ELISA. The c-ELISA is predominantly used to measure the concentration of BTV antibodies in ruminant sera (Reddington et al. 1991; Afshar et al. 1992; Afshar 1994; Hamblin 2004; Kramps et al. 2008; OIE 2008; Chand et al. 2017; Rojas et al. 2019). The basis of c-ELISA is that BTV antibodies in test serum inhibit the binding of HRP-conjugated BTV antibodies to BTV antigen coated on the wells (competitive binding between test serum antibodies and HRP-labeled antibodies to antigen). Binding of HRP-conjugated antibodies are detected by the addition of substrate. The BTV antibodies are quantified by color development and strong color development indicates absence of BTV antibodies in test sera resulting in little or no inhibition of binding of HRP-conjugated antibodies. Weak color development indicates presence of BTV antibodies in test sera, which inhibits the binding of HRP-conjugated antibodies to the antigen coated on the plates.

The c-ELISA is more sensitive, specific and rapid (detects as early as 6 dpi) method than AGID, CFT and plaque neutralization assays (Kramps et al. 2008). The c-ELISA is considered as less expensive and an ideal technique to study the BTV distribution, monitoring the vaccination status as well as planning of control and eradicate policies (Hamblin 2004; OIE 2008). The c-ELISA is being used widely for serological diagnosis of BTV infection and replaced the AGID test (Reddington et al. 1991; Afshar et al. 1992). Unlike AGID, no cross-reactions between BTV antigen and sera containing antibodies to EHDV of deer were reported in c-ELISA (Afshar et al. 1989; Afshar 1994). The c-ELISA is widely used for national and international monitoring of BTV in ruminants during trade (Afshar 1994; Rojas et al. 2019).

BTV structural proteins namely, VP2 and VP5 produce neutralizing antibodies (Lobato et al. 1997). Most of the c-ELISAs for BTV use antibodies to VP7, which is highly conserved among different BTV serotypes and used for detection of group specific antibodies to BTV (Reddington et al. 1991; Afshar et al. 1992; Chand et al. 2017). The DSn of c-ELISA was 97.6% and DSp was 98.0% (Chand et al. 2017). The NS3-based c-ELISA displayed high sensitivity and specificity similar to commercially available VP7-based c-ELISA as serological DIVA test (Tacken et al. 2015). Antigen capture c-ELISA was developed using baculovirus-expressed VP7 antigens for diagnosis of antibodies against BTV and EHDV (Mecham and Wilson 2004). Major drawbacks of this method are false negative results and cross-reactivity between serotypes (Hamblin 2004).

#### 15.2.3. Agar gel immunodiffusion assay

The AGID assay has been commonly used for diagnosis of major group-specific antibodies against VP7 of BTV as a precipitin line (Pearson et al. 1985; Patton et al. 1994; Chandel et al. 2003). The AGID is a classical serological test in which a soluble antigen of BTV is precipitated by specific antibody (known hyperimmune sera) in a clear medium containing 0.9% agarose gel (Pearson and Jochim 1979). The central well contains BTV antigen and peripheral wells contain positive control and test serum alternatively. Following incubation at 37 °C for 24 hours, appearance of a distinct precipitation line in the middle of antigen and serum wells is considered as positive (Pearson et al. 1985; Pearson and Jochim 1979). Soluble antigens and antibodies diffuse passively through the gel towards each other and forming an insoluble immune complex, which is visible as precipitin line.

However, routine use of AGID was decreased due to the development of rapid, sensitive, and specific antibody-detection ELISAs. The AGID is a simple, quick to perform, relatively easy to produce antigens, and qualitative test; however, it lacks sensitivity, specificity, and is not a quantitative test. Further limitations of AGID test are difficult to interpret, cross-reactions with other orbiviruses like EHDV and availability of purified soluble antigens and positive control serum (Pearson and Jochim 1979; Osburn et al. 1981; Sperlova and Zendulkova 2011). The antibodies against host cell proteins react with components of the AGID antigen preparation, which is avoided by including the uninfected cell-antigens as negative controls. Hence, AGID is considered as an alternative test for c-ELISA declared by the OIE (OIE 2008).

The AGID test was compared with c-ELISA for diagnosis of BTV group-specific antibodies in serum from clinically healthy and diseased camels in Gujarat state, India. The seropositivity was 12.5% by AGID and 19.3% by c-ELISA, which difference was non-significant (Chandel et al. 2003). Patton et al. (1994) compared the SNT, AGID and c-ELISA assays for serological detection of BTV-10 or -17 infections in black-tailed deer and fawns. Both SNT and AGID tests gave false positive or false negative erroneous and misleading results. The c-ELISA gave quantitative and accurate results, and is regarded as most useful rapid test for diagnosis of BTV antibodies (Patton et al. 1994).

#### 15.2.4. Serum neutralization test

Neutralization tests like serum neutralization test (SNT), microtiter serum neutralization (MTSN) and plaque reduction neutralization test (PRNT) are commonly used for identification and quantification of the titer of serotype-specific neutralizing antibodies against BTV (Howell et al. 1967; Thomas and Trainer 1970; Afshar 1994; OIE 2008; Rojas et al. 2019). The principle of SNT is that standard concentrations of each known serotype of BTV antigen reacted *in vitro* (BHK-21 or Vero cells) with serial dilutions of unknown/test serum (containing neutralizing antibodies against BTV) resulting in neutralization of virus, which provides evidence for the status of *in vivo* protection against homologous BTV serotype and is used to evaluate the immune status after vaccination (Afshar 1994; OIE 2008; Rojas et al. 2019). The end-point of antibody titration is determined by the highest dilution capable of neutralizing/inhibiting >75% of CPEs. Different BTV serotypes are co-circulating around the world and SNT plays a role in the surveillance program by identifying the antibodies against different serotypes of BTV.

Worwa et al. (2013) optimized plasma neutralization test for diagnosis of BTV-8 neutralizing antibodies using MTSN assay with CPEs detection in Vero cells. The main advantages of SNT are highly sensitive and specific assay, identification of BTV serotype responsible for infection, to evaluate the immune status of individual animals or populations after immunization, absence of cross-reaction with other Orbivirus serogroups while differentiating between the BTV serotypes. The SNT can also be used as a screening test during control and eradication planning for identification of infected animals (Afshar 1994; Worwa et al. 2013; Rojas et al. 2019). The disadvantages of SNT are laborious, time-consuming, and require specific reference viruses for each serotype.

The advent of 96-wells cell culture microtiter plates and multichannel pipetting systems, etc. with accurate diluting and delivering of reagents and cell suspension resulted in the development of MTSN assay, which has replaced the disadvantages of conventional methods like ECEs and cell culture monolayers grown in test tubes or petri dishes (Pearson et al. 1985). The MTSN test is performed using BHK-21 or Vero cells and most commonly used for diagnosis and monitoring of serotype-specific antibodies from infected animal serum and to serotype virus isolates (Afshar 1994; Jochim 1985; Pearson et al. 1985). A serum sample is concluded as positive if there is 75% reduction of CPEs.

The disadvantage of MTSN assay is difficulty in availability of serotype-specific reference antisera to all the recognized BTV serotypes. The factors complicating the establishment of a standardized protocol for the MTSN assay are incubation parameters, cell culture type, quality of cell culture medium, virus strain, virus titer, and quality control of laboratory practices thereby making the comparison of results between the laboratories difficult. Cross-reactions between the serotypes are avoided by performing a second quantitative MTSN assay to determine the relative endpoint of neutralization titer of serum sample. Further, multiple exposures of animals to BTV or related viruses or genetic reassortment of BTVs may also cause misinterpretation of results (Ismail et al. 1987).

BTV has the ability to form plaques in the monolayers of cell cultures maintained under agar medium. This property of BTV was utilized for the development of ‘plaque neutralization assay’ and ‘plaque reduction assay’ for serotyping of BTV and detecting the anti-BTV antibodies (Howell et al. 1967; Thomas and Trainer 1970). Although, plaque neutralization assay was found to be sensitive and specific, it has not been commonly used in diagnostic laboratories.

### 15.3. Other diagnostic techniques

Numerous serogroup-specific diagnostic assays have been developed and evaluated over the years as a complementary or alternative to each other. The IgM-capture ELISA was useful for diagnosis of antibodies against BTV in recently infected animals (Zhou et al. 2001). Immunochromatographic strips (ICS) used for diagnosis of serogroup specific antibodies using rVP7 protein of BTV immobilized on nitrocellulose membranes. The ICS is a rapid test and suitable for the routine serological surveillance of BTV infection in the field. The ICS has high specificity (97.6%) and sensitivity (100%) compared with c-ELISA (Yang, Hua, Chen, Lv, Qin, et al. 2010). The Latex agglutination test is used for diagnosis of anti-BTV antibodies in ruminant serum using latex beads combined with rVP7 protein. Specificity and sensitivity was 99.0% and 93.0%, respectively compared with c-ELISA. Latex agglutination test is easy, rapid and useful for serosurveillance (Yang, Hua, Chen, Lv, Chen, et al. 2010). Double-antigen microsphere immunoassay (MIA) is useful for detection of both serogroup (anti-VP7) and serotype (anti-VP2) specific BTV antibodies simultaneously. The principle of MIA is that each antigen is labeled with different fluorescent beads. The VP7-MIA revealed higher specificity than c-ELISA and VP2-MIA and showed also higher specificity compared with VNT (Breard et al. 2017).

### 15.4. Differential diagnosis

Initial signs of BT are very similar to foot-and-mouth disease (FMD), hence the name pseudo-FMD was given. However, BT lesions are hemorrhagic, edematous and erosive; whereas, FMD lesions are vesicular and erosive (Williamson et al. 2008). The lesion distribution in the tongue differs between BT and FMD. The FMD lesions are typically located at the tip and dorsum of tongue; whereas, BT lesions are located at the back and lateral borders of tongue and are cyanotic. FMD rarely causes eye lesions and goes along with absence of nasal discharge; whereas, these lesions are frequent in BTV infection (Watson 2004). Disease transmission by *Culicoides* vector is also an indicator for differential diagnosis. The BTV outbreaks are sporadic due to seasonal incidence of biting midges and non-contagious; whereas, FMD is non-seasonal, highly contagious and causing higher morbidity than BT. Vesicular stomatitis (VS) is another similar disease of bovines and ovines caused by the genus *Vesiculovirus* and family Rhabdoviridae that could be mistaken for BT (Williamson et al. 2008). The characteristic lesions of VS are vesicles, erosions, ulcers, and crusts limited to the epithelial surfaces of mouth, muzzle, lips, nostrils, feet and teats.

PPR is another infectious and highly contagious disease with similar clinical manifestations as that of BT. In PPR, nasal discharge is common and can be mucopurulent with erosive and necrotic lesions in eyes, oral and nasal tracts. Alimentary tract lesions of PPR are hemorrhagic, erosive and necrotic with diarrhea, and more characteristic respiratory lesions. These kind of lesion distributions are absent in BT, but characteristic coronitis and lameness are present. Like FMD, PPR also causes high morbidity and mortality especially in goats; whereas, BT causes high morbidity and mortality in sheep. Sheep and goat pox, contagious ecthyma and lumpy skin disease in sheep, goat and cattle that can be differentiated by characteristic pock, pustular and crusty lesions and skin nodules with characteristic intracytoplasmic inclusions and proliferative lesions on histopathology (Rao and Bandyopadhyay 2000; Williamson et al. 2008; Tuppurainen and Oura 2012). These kind of wide distribution of skin lesions are absent in BT.

Other diseases or conditions for differential diagnosis are bovine virus diarrhea, malignant catarrhal fever, parainfluenza-3 infection, infectious bovine rhinotracheitis, foot lesions (polyarthritis, footrot and foot abscesses), plant photosensitization, and *Oestrus ovis* (coenurosis) infestation. In cattle and deer, EHDV (caused by similar genus *Orbivirus* and family Reoviridae as that of BTV) can also cause similar symptoms and haemorrhagic lesions (Williamson et al. 2008).

## 16. Control and prevention of BTV

Control and prevention of BTV infection in ruminants have become more important due to the economic impacts of disease and animal movement/trade restrictions (Tabachnick 2004; Ilango 2006; MacLachlan and Osburn 2006; Gunn et al. 2008; Rushton and Lyons 2015; Gethmann et al. 2020). The BTV is easily inactivated at 50 °C for 3 hours and 60 °C for 15 min. Further, BTV is sensitive to pH when treated with acids at less than pH 6 or treated with alkalies at more than pH 8. Common disinfectants, namely phenolic and iodophors agents can deactivate BTV. However, the virus can survive in blood stored at −20 °C for years (OIE 2008). The following most important strategies need to be implemented for rapid control and prevention of BT and related other Orbiviral diseases.

### 16.1. Culicoides vector control

Control of arthropod-borne diseases represents one of the major challenges to veterinary and medical fields (Stuart et al. 2000; Venter et al. 2014; Benelli et al. 2017). Rapid dissemination of arthropod-borne diseases are favoured by co-occurrence of various factors like environmental changes, increased vector activity, more aggressive pathogens, wide host range, and rapid adaption into new hosts (Bethan et al. 2005). The critical strategy for control of vector borne diseases are reducing the population of vectors or reducing the number of potentially infecting bites to the levels where the maintenance of an epizootic becomes not possible. It is rarely or not possible to completely eliminate the *Culicoides* vector populations in India. Vector bites can be prevented by stabling the susceptible animals overnight since midges have nocturnal feeding habits. An amalgamation of several approaches for vector control can yield the best results. The vector control measures must be applied on all premises including horse farms and stables, because *Culicoides* actively feed on horses and manure piles are ideal breeding sites. Ruminants can be protected from *Culicoides* bites by systemic administration of ivermectin S/C. The effectiveness of systemically injectable compounds such as macrocyclic lactones (avermectins) against *Culicoides* has not been studied adequately. However, few studies suggested that these compounds are unlikely useful in reducing the number of potentially infecting *Culicoides* bites (Holbrook and Mullens 1994). The potential strategies to reduce the biting rates on ruminants are trapping (light traps) of adult *Culicoides* midges on farms or use of decoy animal hosts.

#### 16.1.1. Husbandry measures

Animal husbandry modifications can reduce the vector access to susceptible animals. Affected animals and those immediate contacts with affected animals should be housed separately. Housing of other BTV non-susceptible hosts along with susceptible ruminants during the time of maximum vector activity, from dusk until dawn can significantly reduce the biting rates and possibility of transmission of BTV to other animals (Venter et al. 2014). In addition, protection of ruminants in stables can be improved by covering the portals of entry, such as windows and doors with either fine hole mesh fly netting or synthetic pyrethroid insecticides impregnated netting.

#### 16.1.2. Destruction of vector breeding sites

Breeding sites of adult *Culicoides* flies and larval stages should be identified and destroyed or reduced by the drainage of waste-water lagoons, marshlands and standing pools of water, mending leaks and turning off taps can be possible when the sites are few and small (Narladkar et al. 2006). The susceptible animals should be removed from insect resting and breeding sites. The *C. obsoletus*, *C. imicola*, and *C. pulicaris* breed in organic matter rich wet soils and such areas should be drained and removed. Previously, a similar approach was used to control the salt marsh midge populations in the areas of Caribbean and Florida. Further, stable straws and dung heaps should be removed regularly at weekly or shorter intervals that should be less than the developmental period of immature stages.

#### 16.1.3. Adult vector insecticides

Insecticides are regularly used to control the adult *Culicoides* midge populations and thereby reducing the risk of BTV transmission. Insecticides impregnated nets are used to protect the stabled animals from adult midges. Different products, namely insecticides impregnated ear tags, topical administration of ‘pour-on’ products, and washing the animals with synthetic pyrethroids or organophosphate (OP) compounds are used to control both ecto- and endo-parasites of ruminants. Locally applied insecticides on the belly and legs of animals usually have reduced insecticidal efficacy (Mullens et al. 2001). The control of *Culicoides* currently relies on the targeted application of synthetic pyrethroids, namely deltamethrin, cyfluthrin, permethrin and fenvalerate (weekly) or fenvalerate (every second day) with known low mammalian toxicity levels against adult flies in and around animal housing, stable walls, and directly on the target animals. These agents provide protection for 3–5 weeks (Narladkar et al. 2006). Mechanical destruction of *Culicoides* breeding sites like cowpats can be done. The OP insecticidal feed additives such as tetrachlorvinphos are eliminated in the faeces, which are toxic to immature stages of *Culicoides* and should be deposited on vector breeding sites. Saturation spraying strategies are regularly used to control the populations of mosquitoes during human arboviral disease outbreaks. The same strategy can be applied to control *Culicoides* populations; even though, BT is a non-zoonotic arboviral disease of animals.

#### 16.1.4. Insecticides impregnated eartags

Eartags impregnated with synthetic pyrethroid insecticides are effective in reducing the number of bites or strikes of insects on cattle and sheep (Holbrook 1986). These eartags are used for the control of *Culicoides*, but only little information is available.

#### 16.1.5. Vector repellents and attractants (decoy host)

Various compounds, namely para-menthane-3, 8-diol (PMD), N,N-diethyl-m-methylbenzamide or N,N-Diethyl-meta-toluamide (DEET) and KBR:2023 are used as vector repellents and to reduce *Culicoides* biting rates on humans. However, disadvantages of these compounds in animals are complicated and tedious daily application regimens. Further, in animals only limited information is available regarding the withdrawal period of active ingredients that are rapidly absorbed through the skin. Stuart et al. (2000) evaluated the antifeedant and repellent effect of salicylic acid derivatives on the biting midge *Culicoides impunctatus* Goetghebeur. They found that salicyluric acid strongly inhibited the feeding of *Culicoides*. Alkyl substitution of salicylic acid (o-thymotic and o-cresotic acids) has also been found to be effective. Salicyluric acid showed marked protective effects in clinical trials (Stuart et al. 2000).

The DEET is the only commercially available repellent with significant deterrent effects against *Culicoides* but temporary effect for upto 4 hours period for reducing the bites. The DEET is directly applied on target animals at dusk (first 4 hours of night) before the peak attack period (Tweedle and Mellor 2002). Other repellent products that can be used directly on animals are PMD and citronella (*Cymbopogon citratus* L.) oil (Venter et al. 2014). However, continuous use of repellents in ruminants is a difficult challenge and causes uncertain effects in animals and their products as well as the environment (Narladkar et al. 2006). In field, 15% DEET, 0.6% citronella oil, and 0.3% alpha-cyano-cypermethrin were applied on polyester meshes fitted to down-draught suction 220 V UV light traps operating overnight and tested against *Culicoides* species. Interestingly, alpha-cyano-cypermethrin and citronella oil showed no significant repellent effects against *Culicoides*. However, DEET showed significant repellent effect against *Culicoides* species (Venter et al. 2014).

Vector attractants are used as ‘decoys’ to artificially lure the *Culicoides* midges and thereby reducing the biting rates among ruminants. The use of insect attractants, namely semiochemical cues (3-n-propyl-phenol, 1-octen-3-ol and 4-methylphenol) in animals were unexplored. The studies in Scotland and Florida showed insect trapping using attractants reducing *Culicoides* populations. However, no data are available regarding the usefulness of this strategy in animals or if it significantly reduces BTV transmission. In the context of attractants, it has been reported that cattle are generally resistant to BTV, should be allowed to graze on the pastures of sheep to act as ‘decoy hosts’ to reduce the bites in sheep. This strategy may be useful in reducing the BTV transmission to sheep by *Culicoides*, because many species of *Culicoides* are opportunistic feeders. However, more animal density on the farm or grazing pastures might increase the vector population, which in turn increase the risk of disease to entire animal population (Tabachnick 2004). Furthermore, cattle serve as reservoir or amplifying host for BTV resulting in infection to vector midges and subsequent transmission to other animals.

#### 16.1.6. Larviciding

Application of larvicide insecticides namely 5% temephos granulated with gypsum (trademark ABATE, manufactured by American Cyanamid, New Jersey, USA) in *Culicoides* breeding sites provides a slow but sustained release of insecticides for the period up to 30 days (Tweedle and Mellor 2002). Other compounds that target the larval stages of midge vectors are organochlorine, OP compounds and pyrethroid insecticides (Holbrook 1986); however, they have potential environmental ramifications. These compounds can be used on the farms, but only few data are available regarding the effect of this approach on the transmission of BTV to ruminants.

#### 16.1.7. Biorational pesticides or biocontrol of larvae

Alternative approaches for vector control are using ‘biorational’ pesticides that utilize microbiological or hormonal agents for the control of *Culicoides* midges. The microbiological agents are mermithids (*Heleidomermis magnapapula*), bacteria (*Bacillus thuringiensis*), fungi (*Lagenidium giganteum*), and iridescent viruses (Mullens et al. 1995, 2001). *Heleidomermis magnapapula* is a mermithid nematode that parasitizes the larvae of *Culicoides*, but its role as a biocontrol agent needs further investigation (Mullens et al. 1995, 2001; Maheshwari 2012). These agents have been proven to be effective at the laboratory level, but their utility in the field need to be studied.

#### 16.1.8. Eco-friendly control of vectors using neem

The disadvantages of synthetic insecticides are rapid development of resistance, non-target effects on environment and hazardous to animal and human health (Benelli et al. 2017). Currently, various botanicals and phytochemicals have been tested against various arthropod vectors (Benelli et al. 2017). The extracts of neem, namely neem cake and neem kernel oil have been used most widely in organic farming for the control of wide array of arthropod vectors and pests of veterinary and medical importance, because of its highly effective and environmental care properties (Benelli et al. 2017). Neem cake is an eco-friendly and cheap by-product obtained during extraction of neem oil (*Azadirachta indica*). These neem extracts are effective against mosquitoes (Benelli et al. 2017), *Rhipicephalus* spp. and *Ixodes* spp. ticks, cockroaches (genus *Gromphadorhina*, *Blattella* and *Blatta*), house dust mites, raptor bugs (genus *Triatoma*), bed bugs and cat fleas, sandflies, biting and blood sucking lice, poultry mites, beetle larvae feed on poultry plumages, and *Sarcoptes scabiei* mites infesting dogs (Al-Quraishy et al. 2012).

Neem is the only herbal-based biocide accepted by the EU normative (Benelli et al. 2017). Applications of aqueous formulations of neem extraction by-products close to the animal grazing areas in muddy sites effectively reduce the vector populations. Neem extraction by products contain more than 200 bioactive chemicals, of which limonoids (nortriterpenes, e.g. nimbin, azadirachtins, nimbolides and nimbidin) are used for vector control. The neem-based products can also be used to control larval populations of *Culicoides* midges. Neem products are used for disinfection of animal housing, surrounding environment, and as a repellent with application on the animals (Benelli et al. 2017). The mechanism of action of neem includes blocking of post-embryonic development by activating juvenoid substances that blocks the metamorphosis and synthesis of ecdysone. Further, neem has phago-repellent action and causes reduction of oviposition rates in females and egg fertility (Benelli et al. 2017).

### 16.2. Therapeutic measures

The most important initial requirement during BTV outbreaks are to reduce the morbidity and mortality among susceptible ruminants because BT produces serious economic losses to livestock producers (EFSA Panel on Animal Health and Welfare 2017). There are no effective anti-viral drugs and specific therapeutic protocols available for clinically affected animals with BT. Symptomatic therapy include administration of antipyretic, antihistamine, antiphlogistic or non-steroidal anti-inflammatory (NSAID) drugs for reducing the inflammation and pain, which could help for recovery in sheep (Tweedle and Mellor 2002). Affected animals should be handled gently and humanely, isolated properly and stabled in shed, and provided with soft easily digestible feed, shade and water. In clinically affected sheep, BTV induces immuno-suppression, which predisposes the animal to secondary microbial infections, especially development of Pasteurella pneumonia (Umeshappa, Singh, Nanjundappa, et al. 2010). To tackle this complication, suitable antibiotic drugs should be administered systemically.

### 16.3. Monitoring and surveillance strategies

To avoid interference for trade of ruminants and their products, sustainable surveillance and monitoring strategies are needed. Serological screening in the vaccinated animals is impeded by absence of specific assays with DIVA strategies to differentiate the vaccinated and infected animals (Caporale and Giovannini 2010; Anderson et al. 2014; Feenstra et al. 2014; Tacken et al. 2015). All live animals exported/imported for international trade are subject to pre-export and post-import surveillance not more than 24 hours prior to the proposed date of departure and are accompanied by a health certificate. The imported/exported semen for artificial insemination should be screened thoroughly for BT. Main goal of any control and prevention program is limiting the spread of virus to previously unaffected BTV-free regions. Further, Veterinary Officers, other authorities and livestock farmers should act promptly with multi-disciplinary mode to prevent the virus to become enzootic following incursions (Caporale and Giovannini 2010; EFSA Panel on Animal Health and Welfare 2017).

### 16.4. Genetic control tools

The recent advancements in genomic and transcriptomic techniques have allowed us to better understand the gene expressions and biological processes involved in vector competence. The study of transcriptomes of *Culicoides* resulted in better understanding of the functionality of genome, identifying the mid-gut transcripts associated with antihaemostatic and immunomodulatory functions in adult vectors (Campbell et al. 2005), and the genes responsible for vitellogenesis and blood/sugar feeding (Nayduch et al. 2014). Unfortunately, currently no control techniques are available for controlling the biting midges using genetic methods. Analysis of transcriptomes of *Culicoides* may provide new insights for vector control (Nayduch et al. 2014). The most important genetic control tool is use of post-transcriptional gene silencing (PTGS) or RNA interference (RNAi) to control the orbiviral replication or by inhibiting the metabolic function of *Culicoides* vectors by silencing the expression of essential genes. The RNAi technique has been applied in *C. sonorensis*-derived KC cells *in vitro* to target the arbovirus replication (Schnettler et al. 2013) and also studied *in vivo* by injecting the dsRNA intrathoracically in *C. sonorensis* vectors (Mills et al. 2015). Other genetic control tools are release of insects carrying a dominant lethal genetic system (RIDL), sterile insect technique (SIT) and incompatible insect technique (IIT). However, these techniques are evaluated at the laboratory level and their utility in the field against *Culicoides* vectors control need to be investigated in detail (Alphey 2014).

### 16.5. Mathematical modelling

Control of BTV infection in domestic and wild ruminants hypothetically can be achieved by controlling the *Culicoides* vectors or by protecting the susceptible animal host from vector bites. Currently, mathematical modelling is used for the understanding of the relative role of different transmission routes and complex BTV epidemiology in the emergence of diseases. Mathematical modelling is also used to assess the efficiency of control and surveillance programs and supports the evidence-based decision making in animal health. Mathematical modelling computes the various indicators of disease spread using systems of equations to describe the transitions between these states by mathematical expressions (Courtejoie et al. 2018). This is based on the validation of reliable data from each specific region (BTV episystem) and can guide to predict the disease outbreaks and logical mitigation strategies (MacLachlan and Mayo 2013). Courtejoie et al. (2018) have published the systematic review of compartmental mathematical models for BTV transmission and control in Europe.

### 16.6. Lessons learned from past

Experience gained during controlling of infectious diseases from past can help to control and eradication of presently existing diseases. BT and related orbiviral diseases are critical to control; however, possible to follow the control strategies of any similar arboviral diseases of humans or animals like dengue, Eastern equine encephalitis, yellow fever, Zika and West Nile fever for the control of BT (Caporale and Giovannini 2010; Saminathan et al. 2016).

The stamping out or test and slaughter policy has previously been used to control and eradicate emerging and transboundary diseases, notably rinderpest, glanders, etc (Caporale and Giovannini 2010). Recently, these strategies have been used in Europe to control BT, but there was increased societal resistance to large-scale slaughter of healthy domestic and wild ruminants. Stamping out is an inappropriate policy for control and eradication of arboviral diseases like BT. BTV cannot be readily controlled by culling the infected animals, because of involvement of vector and more incidences of subclinical infections in domestic and wild ruminants. The vector midges are widely prevalent and harbour the virus for prolonged period and spread the virus widely (Caporale and Giovannini 2010; Maheshwari 2012). In India, major obstacles for ‘test and slaughter policy’ are inadequate compensation to the owner for culling of infected animals resulted in strong public and political resistance to slaughter of apparently healthy animals. This encourages the owners to hide the affected animals (Saminathan et al. 2016). The test and slaughter or stamping out policies for BT control and eradication are difficult to follow in India due to various economic and social constraints and existence of more populations of vectors and reservoir host (Saminathan et al. 2016).

The European Union Council Directive implemented control strategies in BT affected areas by forming three zones of restrictions, namely a zone of 20 km radius around the infected premises, in which all infected animals should be examined for clinical, pathology and laboratory test to confirm the disease; protection zone including at least 100 km radius of infected zone, in which epidemiological surveillance programme needs to be implemented by serological monitoring of sentinel ruminants and entomological monitoring of vector populations. Animals in this zone were banned for movement until BTV is negative. Vaccination can be done; and surveillance zone extended at least 50 km radius beyond the limits of protection zone. In this zone, activities are similar to those in the protection zone, except vaccination should be prohibited (Caporale and Giovannini 2010). During BTV outbreaks, restriction zones should be created to prevent the disease dissemination. The movement of susceptible animals and their products (meat, semen, ovum and/or embryos) out of the restricted zones were banned to limit the risk of further spread (Caporale and Giovannini 2010; EFSA Panel on Animal Health and Welfare 2017).

During outbreak situations, intra- and inter-country trade restrictions should be followed for effective control of BT. Thus, any interventional program should include the strategies to prevent the movement of susceptible animal species (Caporale and Giovannini 2010; EFSA Panel on Animal Health and Welfare 2017). The priority measure, in BT affected countries are an immediate ban on animal trade within or to other countries, followed by screening of domestic ruminants by observing clinical examination, serological and virological testing, and monitoring of insect vectors (Caporale and Giovannini 2010; Sperlova and Zendulkova 2011; Pandrangi 2013).

### 16.7. Vaccination strategies

Vaccination is an economical and sustainable approach for control of vector borne diseases like BT (Savini et al. 2008; Roy et al. 2009; Caporale and Giovannini 2010; Reddy et al. 2010; Zientara et al. 2010; MacLachlan and Mayo 2013; Pandrangi 2013; Calvo-Pinilla et al. 2014; Feenstra and van Rijn 2017; Ranjan et al. 2019; van Rijn 2019). Prophylactic vaccination has contributed to BT control and significantly reduced the economic losses caused by morbidity, mortality, reproductive problems, and reduced milk production (Kutzler and Weiner 2008; Pioz et al. 2014; McVey and MacLachlan 2015; Mayo et al. 2017). There are 28 existing BTV serotypes, which are showing little cross protection. Currently, live attenuated and inactivated BTV vaccines are available for a limited number of serotypes, but these vaccines have their own pros and cons, including the inability to do DIVA. Intensified and continuous monitoring of BTV serotypes are required to know the exact prevalence of BTV serotypes in different states and inclusion in vaccine development. Even though studies have confirmed that recombinant vaccination technologies are practically applicable in field for prevention of BTV infection in ruminants, expression of immunogenic proteins are challenging due to different conformational structure of each epitope/immunogen, poor stability during storage and serotype-specific immune responses (Savini et al. 2008; Caporale and Giovannini 2010; Zientara et al. 2010).

#### 16.7.1. Live attenuated vaccines

The first live attenuated monovalent BTV vaccine was developed by serial passaging in sheep and this vaccine was used from 1907 to 1943. But this vaccine failed to provide immunity against many other serotypes of BTV that circulate in South Africa. Hence, this vaccine was discontinued. Presently used live attenuated BTV vaccine in the United States (only vaccination in sheep), South Africa, Italy, Israel, Bulgaria, India, France, Spain, and Turkey contains five different combinations of serotypes of BTV attenuated by continuous passaging in ECEs and BHK-21 cells (Taylor and Mellor 1994; Caporale and Giovannini 2010; McVey and MacLachlan 2015; Ranjan et al. 2019; van Rijn 2019). The modified live virus (MLV) vaccines of BTV have many advantages like cost-effective, low dose of attenuated virus (antigenic mass) are required to trigger protective immune responses and single dose is sufficient to stimulate neutralizing antibodies (Monaco et al. 2004; Savini et al. 2004; Venter et al. 2004). The advent of reverse genetics technologies resulted in generation of immunogenic BTV epitopes, which have vaccine potential (Nunes et al. 2014).

The disadvantages of using MLV vaccines of BTV in endemic areas are reoccurrence of clinical disease in vaccinated animals like viremia, reduced milk production in lactating animals, and teratogenic effects and abortion in pregnant animals (MacLachlan and Osburn 2006, 2008, 2017; Savini et al. 2004). Further, genetic reassortment of vaccine viruses resulted in emergence of virulent BTV, contamination of vaccine viruses with other viruses, and trade constrains due to inability to differentiate the naturally infected animals with field strains of BTV from vaccinated animals (Osburnet al. 1981; Monaco et al. 2004). It is well established that vaccination with MLV vaccines resulted in transmission of BTV by *Culicoides* midges to other susceptible host. Hence, MLV vaccines should be injected during the vector inactive period to reduce genetic reassortment of field strains of BTV with vaccine viruses (Venter et al. 2004; Caporale and Giovannini 2010; McVey and MacLachlan 2015).

#### 16.7.2. Killed or inactivated vaccines

Killed vaccines against BTV are first developed by inactivating whole BTV with ultraviolet radiation, heat or chemicals using binary ethylenimine (BEI) or hydroxylamine in 1975 (Parker et al. 1975; Savini et al. 2007). In Europe, killed BTV-2 vaccine was commercially introduced in 2005 (Zientara et al. 2010). Subsequently, inactivated polyvalent vaccines were developed by including BTV-1, -4, -8, and -9. The advantages of killed vaccines are safe, reduced economic losses due to infection and safe trading of animals, and absence of reoccurrence of disease, viremia in vaccinated animals, disease transmission by vectors, reversion of viral virulence and reassortment with field strains of BTV (Savini et al. 2008; Caporale and Giovannini 2010; Zientara et al. 2010). The disadvantages of killed vaccines are costly production, require complete inactivation to avoid any residual infectious virus, inactivation process reduces immunogenicity, multiple doses and large amounts of vaccine required to trigger protective immunity, in vaccinated animals relatively short duration of protective immunity, and short shelf life due to reduced stability (Mayo et al. 2017). Killed BTV-8 vaccine was used during the outbreak of highly pathogenic BTV-8 in Europe in 2006, that significantly reduced the economic losses (Caporale and Giovannini 2010; Breard et al. 2011; Mayo et al. 2017).

Ramakrishnan et al. (2006) developed BEI-inactivated BTV vaccine for sheep. BEI-inactivant and saponin-adjuvant combination of BTV vaccines are safe and most suitable for inducing protective immune responses. Umeshappa, Singh, Pandey, et al. (2010) evaluated the cross-protective efficacy of BEI-inactivated BTV-1 vaccine against virulent BTV-23 serotype challenge in Indian native sheep. This inactivated BTV-1 vaccine-induced significant CMI responses (increased CD4 and CD8 T cells, IFN-α, IL-2, IL-12 and IFN-γ) and reduced the severity of heterologous BTV-23 infection. Reddy et al. (2010) developed inactivated adjuvanted pentavalent vaccine containing BTV-1, -2, -10, -16, and -23. This vaccine was found to be sterile, safe, potent and currently used in India for control and prevention of BT. This vaccine technology was transferred to Indian Immunologicals Ltd., Hyderabad; Biovet Pvt. Ltd., Bangalore; and M/s Sanvita Biotechnologies Pvt. Ltd., Hyderabad for commercial production. Further research is needed in order to evaluate the safety and efficacy of this pentavalent inactivated vaccine in goats and cattle, because these animals act as subclinical carriers and subsequent transmission of the virus. The BEI inactivated pentavalent (BTV-1, -2, -10, -16, and -23) vaccine adjuvanted with montanide was protective in sheep after challenge with homologous serotypes (Bitew et al. 2017).

#### 16.7.3. Subunit vaccines

The advantages of subunit vaccines are the DIVA capabilities (Feenstra and van Rijn 2017). Previous studies showed that vaccines developed using purified VP2 protein stimulate the protective immune responses against infection with homologous BTV serotypes in sheep, because VP2 protein is important to induce the neutralizing antibodies (Huismans et al. 1987). However, the disadvantages of this vaccine are that purification of large scale of VP2 protein is expensive (Huismans et al. 1987). The advent of reverse genetics technologies have resulted in cloning of VP2 gene using different vectors for large-scale production of VP2 protein (Huismans et al. 1987). Various VP2 based subunit vaccines against BT were developed by expressing in baculovirus (Zientara et al. 2010; MacLachlan and Mayo 2013; Pandrangi 2013; Calvo-Pinilla et al. 2014; Feenstra and van Rijn 2017; Ranjan et al. 2019; van Rijn 2019).

Recently, a subunit vaccine containing purified VP2, NS1 and NS2 proteins of BTV expressed in baculovirus and *E. coli,* and adjuvant with immunostimulating complex AbISCO-300 was developed and evaluated in cattle. This vaccine induced sufficient humoral and CMI responses after booster vaccination and provided protection against different serotypes of BTV (Anderson et al. 2013). Further, this vaccine was evaluated in calves and provided protection against BTV-8 challenge, 3 weeks after booster vaccination. The CMI response (T-lymphocytes) directed against NS1 and NS2 proteins of BTV provided cross-protection to varying VP2 serotypes (Anderson et al. 2014). The subunit vaccine containing structural proteins VP2 (two domains), VP5 (VP5Δ1-100) and full-length VP7 of BTV-4 was produced by expressing in bacteria as soluble glutathione S-transferase fusion-proteins. This vaccine was evaluated by immunizing in Balb/c and IFNAR^(−/−)^ mice, and challenged with homologous live BTV-4. The immunised mice were survived, because VP2 domain induced serotype-specific neutralizing antibodies, and failed to develop clinical signs of infection. However, mice immunised with two VP2 domains with or without VP5Δ1-100 and VP7, and challenged with heterologous BTV-8 serotype showed mortality by 7 dpi (Mohd Jaafar et al. 2014).

Another subunit vaccine of BT was developed by incorporating VP2, VP7 and NS1 proteins of BTV, which are expressed in avian reovirus muNS-Mi microspheres. This vaccine elicited strong protective immune responses in IFNAR^(−/−)^ mice (Marín-López et al. 2014). Experimental subunit vaccine against BTV-4 was developed by fusion of VP2 protein with antigen presenting cell homing (APCH) molecule in the baculovirus insect cell expression system. The APCH-VP2 protein induced the CMI responses and neutralizing antibodies in guinea pig, cattle and IFNAR^(−/−)^ mice (Legisa et al. 2015).

#### 16.7.4. Virus-like particles

Virus-like particles (VLPs) are non-infectious molecules that closely resemble viruses. The VLPs are self-assembling viral structural subunits, and virus replication and gene expression cannot occur due to lack of nucleic acids (Liu et al. 2016). Vaccines containing VLPs provide strong immunogenicity as that of wild-type viruses without producing any clinical disease. Further, VLPs are highly suitable for presentation of foreign antigens on their surface and efficient delivery to antigen-presenting cells (Liu et al. 2016). The VLPs revealed an icosahedral structure as that of wild-type BTV particles with 86 nm in diameter (Hewat et al. 1994). The advantage of VLP based vaccines are DIVA strategy due to the absence of non-structural or structural minor enzymatic proteins namely, VP1, VP4 and VP6. Intracellular location and conformation of VLPs are similar to that of BTV; therefore, VLPs provide better protection against BTV than subunit vaccines (MacLachlan and Mayo 2013; Pandrangi 2013; Calvo-Pinilla et al. 2014; Feenstra and van Rijn 2017; Ranjan et al. 2019; van Rijn 2019).

Minute amount of VP2 with immunostimulating adjuvant (ISA-50) elicited neutralizing antibodies and protected the sheep against challenge with virulent homologous BTV. The VP2 plays main role in inducing neutralizing antibodies and protection, and VP2 presented in the form of VLPs as vaccine, it provides 25–50 fold more protection than VP2 or VP5 based subunit vaccines (Roy et al. 1992). The VLPs for VP2 protein of BTV have been generated for multiple serotypes like BTV-1, -2, -10, -13, and -17 by expressing in recombinant baculovirus (French et al. 1990). Combinations of five serotypes of VLPs were used as vaccine in sheep and challenged with selected homologous or heterologous serotypes. The virus-neutralizing antibody responses were measured. Two doses of VLPs elicited long-lasting protective immune responses in sheep against homologous virulent virus and partial protection against heterologous BTV serotypes (Roy et al. 1994). The efficacy of VLPs has been tested in large animal experiments with 50–200 sheep per trial. Each time, VLP vaccination afforded protection against challenge with homologous BTV serotype (Roy 2004). Merino sheep were vaccinated with either monovalent BTV-1 VLP or bivalent VLPs containing BTV-1 and -4, and challenged with virulent BTV-1 or BTV-4. The BTV-1 VLP delivered as monovalent or bivalent immunogen protected the sheep from challenge with virulent BTV-1. There is some interference in the protective response by BTV-4 in bivalent VLP vaccine (Pérez de Diego et al. 2011).

The major structural core-like particles (CLPs) of VP3 and VP7 of BTV were developed by expressing in recombinant baculovirus (French and Roy 1990). The double-shelled VLPs containing four major structural proteins of BTV namely, L2 and M5 genes encodes the outer capsid proteins VP2 and VP5, respectively, and two core proteins VP3 and VP7 were expressed in recombinant baculovirus system. High titers of neutralizing antibodies were induced against homologous BTV serotype (French and Roy 1990; French et al. 1990). Stewart et al. (2012) developed BT-VLPs (containing VP2, VP3, VP5, and VP7), sub-viral and inner CLPs (VP3 and VP7) using recombinant baculovirus expression system. The protective efficacy of VLPs and CLPs were investigated in sheep. All VLP-vaccinated animals developed neutralising antibodies to western and eastern lineage of virulent BTV-1. Further, post-challenged animals had no clinical disease and viraemia. In contrast, CLP-vaccinated animals did not induce any neutralising antibodies and failed to prevent the clinical disease and viraemia. The lack of protection by CLPs was due to the absence of outer capsid protein VP2. Thuenemann et al. (2013) developed BT-VLPs in plant based expression vector system namely, *Nicotiana benthamiana* using cowpea mosaic virus-based HyperTrans (CPMV-HT), which showed strong antibody responses in sheep. Further, this vaccine provided protective immunity against challenge with South African BTV-8 field isolate.

#### 16.7.5. DNA vaccines

The DNA vaccines utilize novel technology by which injection of genetically engineered DNA is aimed to produce efficient humoral and CMI responses. The strategies involved in DNA vaccines are to use DNA plasmids expressing one or more antigens to induce protective immune responses against viruses (Kutzler and Weiner 2008; Ranjan et al. 2019; van Rijn 2019). The advantages of DNA vaccines are safety, high biological stability at ambient temperatures, cost-effective, rapid manufacturing, and potent induction of Th1 responses. Development of DNA vaccines expressing conserved protective antigens resulted in enhanced immune responses and reduced number of multi-serotype vaccinations required (Zientara et al. 2010; MacLachlan and Mayo 2013; Pandrangi 2013; Calvo-Pinilla et al. 2014; Feenstra and van Rijn 2017). DNA vaccine strategies are used for the development of safe marker (DIVA) vaccines. The disadvantages of homologous DNA vaccines are low immunogenicity; however, it can be used as heterologous vaccines in combination with recombinant viruses to prime the immune system as immunoboosting agents. This heterologous vaccination strategy was tested in various studies (Calvo-Pinilla, Rodriguez-Calvo, Sevilla, et al. 2009; Calvo-Pinilla et al. 2012; Jabbar et al. 2013). Ineffective delivery of DNA vaccines resulted in reduced effectiveness. Use of DNA vaccines on large scale are limited.

The IFNAR^(−/−)^ mice inoculated with heterologous prime boost vaccination comprising of naked DNAs and recombinant modified vaccinia virus Ankara (rMVA) expressing VP2, VP5 and VP7 proteins protected the mice completely against BTV-4 challenge by inducing significant levels of neutralizing antibodies. Further, DNA/rMVA-VP2 and -VP7 proteins triggered BTV specific T-cell responses, which might be responsible for protection. This vaccine provides the advantage of DIVA properties (Calvo-Pinilla, Rodriguez-Calvo, Sevilla, et al. 2009). Similarly, IFNAR^(−/−)^ mice inoculated with heterologous prime boost vaccination (expressing VP2, VP7 and NS1 proteins) protected the mice against BTV-4 challenge by stimulating CD8^+^ T cell responses. Interestingly, this promising multi-serotype vaccine against BTV provides cross-protection against lethal doses of heterologous BTV-8 and BTV-1 (Calvo-Pinilla et al. 2012). The BTV-DNA vaccine provided partial protection (no clinical protection and viremia was delayed) when plasmids expressing VP2, VP7 and NS1 proteins of BTV-4 were used as vaccine in IFNAR^(−/−)^ mice. Mice vaccinated twice with each plasmid two weeks apart and challenged with BTV-4 were partially protected (Calvo-Pinilla et al. 2014). Further, protective efficacy of recombinant vaccines encoding VP2, VP5 or VP7 proteins of BTV-8 were tested in mice by administration of either homologous (rMVA/rMVA) or heterologous (DNA/rMVA) prime boost vaccination. All the animals that received VP2 alone generated neutralising antibodies and VP7 alone were not protected. However, mice vaccinated with rMVA/rMVA or DNA/rMVA-VP2, -VP5 and -VP7 or -VP2 alone were protected (Jabbar et al. 2013). Li et al. (2015) evaluated DNA vaccines and recombinant fowlpox virus (rFPV) vaccines expressing VP2 alone or VP2 in combination with VP5 proteins of BTV-1 in mice and sheep. The DNA vaccine prime (co-expressing VP2 and VP5) followed by rFPV vaccine boost (co-expressing VP2 and VP5) induced high titers of neutralizing antibodies in sheep.

#### 16.7.6. Disabled unfectious single animal vaccine

The disabled unfectious single animal (DISA) vaccines have been developed using reverse genetics technology. In this vaccine, one important gene is deleted from the engineered virus resulting in creation of immunogenic vaccine virus (Ranjan et al. 2019; van Rijn 2019). The advantages of DISA vaccines are absence of residual virulence, reversion of virulence, horizontal spread by vectors, reassortment with field virus, and vertical transmission unlike live-attenuated vaccines. The BT-DISA vaccines contain replicating viruses like live-attenuated vaccines, but absence of non-essential NS3/NS3A proteins. The BT-DISA vaccines containing VP2 protein-induced highly protective immune responses rapidly by stimulating serotype-specific neutralizing antibodies (Calvo-Pinilla et al. 2014; Feenstra and van Rijn 2017; Mayo et al. 2017; van Rijn et al. 2017). The DISA vaccine may act as safer alternative to MLV vaccines. Absence of antibodies against NS3/NS3A in BT-DISA vaccination enables DIVA principle. However, DISA vaccines require higher immunizing dose or several doses of engineered viruses like inactivated vaccines (Feenstra et al. 2014; 2015; Tacken et al. 2015).

Feenstra et al. (2014) developed a DISA vaccine by deleting the NS3/NS3A gene in virulent BTV-8 resulted in generation of non-virulent BTV-8 and local replication leads to seroconversion and reduced viraemia in sheep. Further, DISA vaccine containing live-attenuated BTV-6 without NS3/NS3A is a promising vaccine candidate and protected against BTV challenge in sheep. The DISA vaccine in which genome Seg-2 of BTV-8 and other serotypes were exchanged provided serotype-specific protection in sheep. Feenstra et al. (2015) developed a next-generation DISA vaccine with 1/16 chimeric VP2 containing amino acid region 249-398 of BTV-16 inducing neutralizing antibodies and protective in sheep against BTV-1 and BTV-16. The optimal route for BT-DISA vaccination is intramuscular. The protective dose of DISA vaccine has not been determined yet, but it is expected to be significantly lower when compared to currently available BT vaccines (van Rijn et al. 2017).

#### 16.7.7. Disabled infectious single-cycle vaccine

Like DISA vaccines, disabled infectious single-cycle (DISC) vaccine viruses are produced by reverse genetics technology and DISC virus particles infect the target cells of vaccinated animals only once, due to deletion of important genes. The DISC viruses are highly protective, DIVA capability, and act as promising alternative vaccine agent against currently available killed and MLV vaccines. The DISC BTV vaccines produce an aborted infection resulting in expression of viral proteins in the vaccinated animals as that of MLVs and good safety as that of inactivated vaccines. The disadvantages of DISC vaccines are that complementary cell lines are required for large scale vaccine production and high protective dose, and the minimal protective dose not been determined yet (Feenstra and van Rijn 2017; Mayo et al. 2017).

Matsuo et al. (2011) generated a DISC vaccine virus strain by deleting the important gene that encodes VP6 protein (Seg-9) of BTV-1. The VP6-deficient BTV-1 mutant virus (no viral helicase) acted as potential vaccine candidate. Further, defective BTV-8 strain was generated by reassorting the two RNA segments that encode two outer capsid proteins (VP2 and VP5) from highly pathogenic BTV-8 and eight RNA segments of BTV-1 DISC virus. The BTV-1 and BTV-8 DISC viruses were highly protective in sheep after challenge with virulent BTV strains. The VP6-deficient DISC vaccines have been tested in sheep and cattle (Celma et al. 2013). Cattle and sheep were vaccinated with DISC viruses either singly or in cocktail form as a multivalent vaccine (BTV-2, -4, -8, -10, -13, -21, and -24 serotypes) developed neutralizing antibodies for specific serotypes, and no clinical signs and viremia were noticed (Ranjan et al. 2019; van Rijn 2019).

## 17. Conclusion and future perspectives

BT is endemic in India, because of favourable climatic conditions and density of natural host population, which are essential for survival of *Culicoides* vector and BTV. Further, 23 serotypes have been reported from India as of now, out of 28 serotypes of BTV exist globally due to its ability to reassort genome segments. However, data on temporal or spatial distribution of particular serotype of BTV are not available in India. South Indian states are frequently and more severely affected when compared to north Indian states and most of the BTV serotypes were isolated from former. Continuous sero-surveillance programs are needed to monitor the endemicity, emerging and re-emerging status of BTV serotypes in India. This will help to identify the most commonly circulating serotypes in endemic areas, which will be utilized for development of multivalent vaccines leading to control and eradication of BT. Regular monitoring of flocks/herd immunity against different BTV serotypes in endemic areas are needed. Dramatic changes in climate, deforestation, anthropogenic factors and global warming resulted in emergence of novel BTV serotypes outbreaks with variable pathology, which warns the livestock rearing community and arboviral researchers. Economically important orbiviral diseases viz. BT, African horse sickness, and epizootic haemorrhagic disease are not zoonotic like other orbiviruses (Changuinola virus, Corriparta virus, Kemerovo virus, Yunnan orbivirus and Orungo virus); hence, these diseases do not cause any risk to the public and biomedical research community.

Vaccination is the ideal method for the control of BTV infection. Even though recombinant vaccination technologies are giving promising results, most of them are ended at laboratory levels. Hence, vaccination strategies to curtail BTV infection are supposed to be dependent on conventional/traditional vaccine strategies like killed and MLV vaccines with all of its inherent pros and cons. Significant research is needed in the development of safe and potent new generation BTV vaccines with DIVA capabilities, applicable to all serotypes due to frequent emergence of novel BTV serotypes. The DIVA vaccines would be a useful tool for the serosurvillence, prophylactic protection, control and eradication of BT in endemic areas. Still, neither of these vaccines have been licensed and/or commercialised nor is ready for large scale production, and many of them are ended at laboratory level. Even though, new vaccine candidates show improvement, their final vaccine profile have not been definitely determined yet, likely more cost per protected animal than current vaccines, limited serotypes combinations, and limited market potential have prevented their commercial use. In endemic areas, judicious vector control programs need to be implemented. In those areas, where BT is absent or outbreak happened in last decade, it is necessary to establish sentinel herds of cattle to detect new outbreaks and to identify whether BTV is established in that areas or not.

The role of wild animals and vectors in wildlife habitats in the epidemiology of BT are in need to be studied in detail. Wild ruminants act as reservoir or maintenance host for BTV infection due to long-lasting viraemia, long-term carrier state and vector maintenance, and play an important role in its transmission. The spectrum of wild animal species as BTV sentinels like deer are needed for control of BTV. The existence of interconnected domestic and wildlife cycles could be responsible for the maintenance of BTV. Many questions still need to be answered through research like molecular determinants for reassortant of BTV and its virulence, genetic determinants of transplacental transmission of each BTV strain, molecular pathogenesis of BTV in pregnant animals and foetuses, kinetics of BTV in dams and fetuses, molecular pathogenesis of BTV induced thrombo-haemorrhagic disease, and the genetic basis for susceptibility of species/breeds to BTV. During BTV outbreaks associated with reproductive disorders (as in the case of BTV-8 in Europe), it is necessary to quickly collaborate between the specialists in the field of virology, pathology and reproduction.
